# Design, Synthesis, and Acaricidal Activity of 2,5-Diphenyl-1,3-oxazoline Compounds

**DOI:** 10.3390/molecules29174149

**Published:** 2024-08-31

**Authors:** Yuming Chen, Jiarui Tian, Yuhao Tan, Yuxiu Liu, Qingmin Wang

**Affiliations:** State Key Laboratory of Elemento-Organic Chemistry, Research Institute of Elemento-Organic Chemistry, College of Chemistry, Frontiers Science Center for New Organic Matter, Nankai University, Tianjin 300071, China; mchen19@163.com (Y.C.); 2120231288@mail.nankai.edu.cn (J.T.); 2120231296@mail.nankai.edu.cn (Y.T.)

**Keywords:** scaffold hopping, ring equivalent, intermediate derivatization, oxazoline, acaricidal activity, chitin synthase

## Abstract

By using a scaffold hopping/ring equivalent and intermediate derivatization strategies, a series of compounds of 2,5-diphenyl-1,3-oxazoline with substituent changes at the 5-phenyl position were prepared, and their acaricidal activity was studied. However, the synthesized 2,5-diphenyl-1,3-oxazolines showed lower activity against mite eggs and larvae compared to the 2,4-diphenyl-1,3-oxazolines with the same substituents. We speculate that there is a significant difference in the spatial extension direction of the substituents between the two skeletons of compounds, resulting in differences in their ability to bind to the potential target chitin synthase 1. This work is helpful in inferring the internal structure of chitin synthase binding pockets.

## 1. Introduction

**Figure 1:**
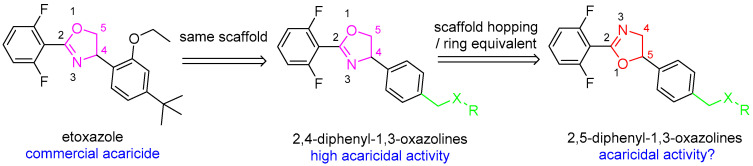
Design of 2,5-diphenyl-1,3-oxazolines.

Mites are characterized by their small size, fast reproduction, and drug resistance, posing a serious threat to agricultural production [1]. Continuously researching and developing acaricides with novel structures and mechanisms of action is an important measure for pesticide management worldwide. 2,4-Diphenyl-1,3-oxazoline compounds are a class of compounds with high acaricidal activity [2], and etoxazole (Figure 1) is the only commercially available variety with this skeleton [3]. Recently, there have been literature reports that, as a chitin synthesis inhibitor, the target of etoxazole is likely chitin synthase 1 [4,5,6]. However, due to the lack of single crystals of chitin synthase 1 in mites and the low homology between mites and insects or fungi [5,7,8], it is still difficult to directly design chitin synthase inhibitors by modeling the precise three-dimensional structure of chitin synthase in mites through homology. 

Pharmacophore assembly and intermediate derivatization [9] are the most used design methods for drugs and pesticides. Connecting different functional groups to the same intermediate can quickly result in a large number of structurally similar molecules, which is helpful for studying molecular structure–activity relationships and further optimizing structures. Taking our work as an example, we have recently conducted extensive work on the effect of the 4-phenyl para substituent of 2,4-diphenyl-1,3-oxazolines on acaricidal activity. By reacting with the same benzyl halogen intermediate, we have introduced ethers, thioethers, oxime ethers, and nitrogen-containing heterocycles to the molecules and optimized a batch of compounds with better activity than etoxazole [10,11,12,13,14] (Figure 1).

Bioisosterism and scaffold hopping are other commonly used methods in traditional drug and pesticide optimization [15,16,17,18]. New molecules derived from commercialized drugs or highly active compounds through bioisosterism and scaffold hopping may still bind to the same biological target in their original binding pocket. Nevertheless, due to slight differences in hydrophobicity, electronic properties, hydrogen bonding, and substituent volume or orientation, the binding site or binding abilities between molecules and the targets may change, leading to changes in the selectivity, metabolic properties, transport characteristics, or the activity level. Ring equivalent is one of the categories of scaffold hopping. The application of ring equivalent has led to the discovery of many innovative agrochemicals. For example, when changing the benzene ring to a pyrimidine ring, pyrimidinyldioxy strobilurin azoxystrobin exhibited enhanced fungicidal activity [19]. Furthermore, highly nematicidal trifluoromethanesulfonamides were developed by changing benzene rings to pyridine or thiophine rings [20], and when introducing a 1,3,4-thiadiazole thione moiety instead of a 1,3,4-oxadiazole thione moiety of a phenylthiazole derivative, the compound showed remarkable antibacterial activity [21]. In addition, by adopting both ring equivalent and amide bond reversal strategies, large amounts of trifluorobutene nematicides have been designed, and quite a lot of them have been found to have high nematicidal activity [22,23,24]. 

The molecules 2,5-diphenyl-1,3-oxazoline and 2,4-diphenyl-1,3-oxazoline are very similar in three-dimensional structure, and we speculate that their binding mode to the target may also be similar. In order to compare the activity differences between these two isomers, this study applied both ring equivalent and intermediate derivatization strategies to design and synthesize a series of 2,5-diphenyloxazoline compounds in which the 2-phenyl ring still maintains 2,6-difluoro substituents while the substituent on the 5-phenyl para position specifically refers to the type of substituents in highly active 2,4-diphenyloxazolines for a better comparison and analysis of the acaricidal activity (Figure 1).

## 2. Result and Discussion

### 2.1. Synthesis

**Scheme 1:**
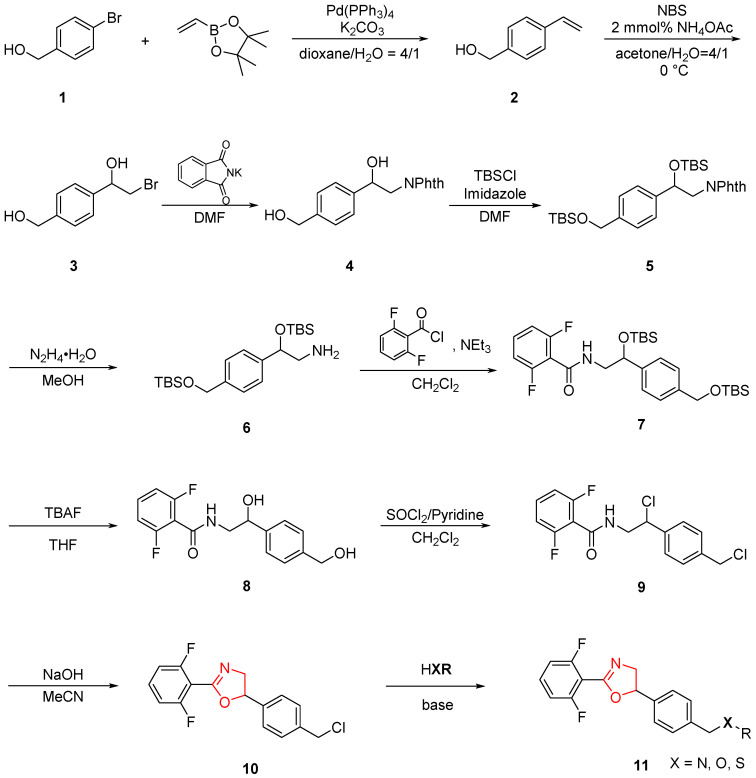
Synthesis route for target 2,5-diphenyl-1,3-oxazolines.

The route for synthesizing key intermediate **10** and target compounds **11** is shown in Scheme 1. Firstly, *p*-bromobenzyl alcohol (**1**) and vinylboronic acid pinacol ester were used to prepare olefin **2** by Suzuki coupling. Next, the obtained olefin **2** underwent bifunctionalization with *N*-bromo-succinimide (NBS) to form bromoethanol derivative **3**, which was then nucleophilically substituted by potassium phthalimide to obtain intermediate **4**. Due to the difficulty in separating and purifying the highly polar and water-soluble polyhydroxylamines generated by direct hydrazinolysis of **4**, it was necessary to protect hydroxyl groups before hydrazinolysis. Therefore, through hydroxyl protection with *t*-butyldimethylsilyl chloride (TBSCl), hydrazinolysis with hydrazine hydrate, acylation with 2,6-difluorobenzoyl chloride, and deprotection with tetrabutyl ammonium fluoride (TBAF), intermediate **4** was successfully converted to dihydroxy compound **8**. The intermediate **8** chlorinated with SOCl_2_ and underwent cyclization under alkaline conditions to obtain key intermediate **10** with 2,5-diphenyloxazoline skeleton and a benzyl chloride moiety, which was then reacted with different nucleophilic reagents (HXR, X = N, O, S), such as nitrogen-containing heterocycles, substituted alcohols, phenols, and thiophenols, to obtain target 2,5-diphenyloxazoline compound **11**. Most of the selected nucleophilic reagents were used to synthesize 2,4-diphenyloxazoline analogues with high acaricidal activity so that the substituents of the newly synthesized 2,5-diphenyloxazolines were the same as those of the 2,4-diphenyloxazolines, facilitating activity comparison. The synthesis refers to the synthesis conditions of 2,4-diphenyloxazoline derivatives; that is to say, different solvents and bases needed to be selected for different nucleophilic reagents to ensure yield. Specifically, when synthesizing **11a**, dimethylformamide (DMF) was used as the solvent and potassium phthalimide was used as the nucleophilic reagent. When synthesizing **11b**–**11g** and **11t**–**11x** using nitrogen heterocycles or thiophenols, DMF was used as the solvent, and NaH was used as the base. When preparing **11h**–**11m**, alcohols were used as the nucleophiles; at this time, acetonitrile was used as the solvent, NaOH was used as the base, and NaI was added to promote the reaction. When preparing **11n**–**11s**, phenol or oxime was used as the nucleophiles, and NaOH could be replaced with K_2_CO_3_. When synthesizing **11y**, sodium *p*-toluenesulfinate in acetonitrile was used, and KI was also added to promote the reaction. In addition, during the synthesis of **11s**, due to the raw material being (*E*)-2-trifluorobenzaldehyde oxime, the resulting product was also in the *E* configuration.

### 2.2. Acaricidal Activity

**Figure 2:**
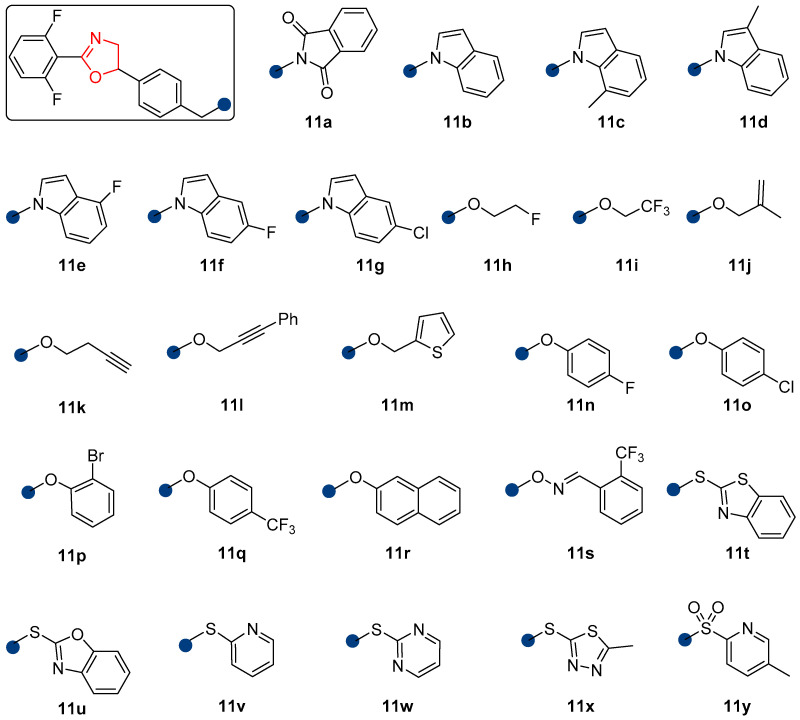
Structure of synthesized 2,5-diphenyl-1,3-oxazolines.

**Figure 3:**
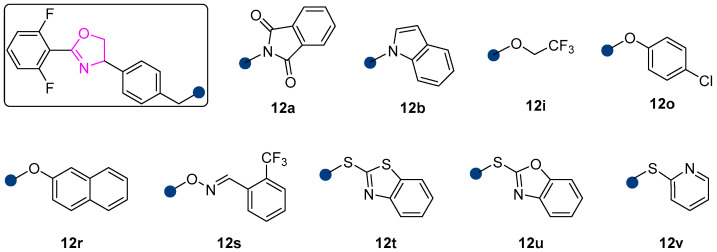
Previously synthesized 2,4-diphenyl-1,3-oxazolines **12** with high acaricidal activity.

The synthesized 2,5-diphenyloxazolines **11a**–**11y** (Figure 2) were evaluated for their acaricidal activity against mite eggs and larvae together with intermediates **8**–**10** and several 2,4-diphenyloxazolines **12** (Figure 3), with etoxazole as a control. The data are listed in Table 1.

The table shows that most of the 2,5-diphenyloxazolines and intermediates **8**–**10** exhibited 100% mortality against mite eggs at a concentration of 100 mg/L; six of them, **11l, 11o**, **11q**, **11t**, **11y**, and compound **9**, still maintained 100% mortality at a concentration of 10 mg/L. However, when the concentration was further reduced, none of them showed good activity. In contrast, all 2,4-diphenyloxazolines and etoxazole **12** exhibited 100% mortality against mite eggs even at 5 mg/L. Specifically, **12s** is a candidate acaricidal agent that we previously obtained that has excellent acaricidal activity against mite eggs, while **11s** did not show activity at a concentration of 100 mg/L, and the difference in activity levels between the two was very significant. From this, it can be seen that when the basic scaffold changes from 2,4-diphenyloxazoline to 2,5-diphenyloxthiazoline, there is a significant change in activity, suggesting that although these two structures appear similar, their configurations in the binding pocket may not necessarily be similar. 

For mite larvae, although the mortality rates of 2,5-diphenyloxazolines were lower than those of the corresponding 2,4-diphenyloxazolines, they were higher than their mortality rates against mite eggs. This was definitely different from the pattern of 2,4-diphenyloxazolines, which had a higher acaricidal activity against mite eggs than mite larvae. This indicates that mite eggs and mite larvae have different sensitivities to different acaricidal reagents. Since the 2,5-diphenyloxazolines do not have comparable activity to etoxazole against either mite eggs or larvae, they do not deserve further investigation.

It is worth noting that intermediate **9**, an amide compound, has significantly higher acaricidal activity against mite larvae (according to Table 2, its IC_50_ is around 1 mg/L, and the IC_50_ of etoxazole is about 0.3 mg/L), indicating that its acaricidal mechanism may be different from that of the other compounds. Some compounds containing *N*-phenylethylbenzamide skeleton have been reported to have antiparasitic, nematicidal, and fungicidal activities [25,26,27,28], but no such compounds have been reported to have acaricidal activity. Further optimization and research are needed to investigate the acaricidal activity and mechanism of action of these types of compounds. In addition, due to the presence of chiral atoms in this type of molecule, the activity of the racemic compounds we previously tested cannot reflect the enantiomeric relationship between activity and chiral isomers. The synthesis and biological activity of chiral molecules, including 2,4-diphenyloxazolines and intermediate **9** analogs, will be our future research focus.

## 3. Materials and Methods

### 3.1. Preparation of Test Compounds 

The synthesis route for intermediates **2**–**10** and target compounds **11a**–**11y** is depicted in Scheme 1, the synthetic procedures are described below, and the detailed data and spectra are provided in the Supplementary Materials. Reagents for preparation were purchased from commercial sources including Tianjin Bohua Chemical Reagent Co., Ltd,; Bide Pharmatech Ltd., HEOWNS, and Energy Chemical, and were used as received. Compounds **12a** [12], **12b** [12], **12i** [13], **12o** [13], **12s [10,29]**, **12t** [11,29], **12u [11]**, and **12v** [11], used as controls, were the samples we previously prepared, and their methods can be found in the published literature. 

#### 3.1.1. (4-Vinylphenyl)methanol (**2**) 

In a 500 mL round-bottom flask, p-bromobenzyl alcohol (18.70 g, 100 mmol) and vinylboronic acid pinacol ester (18.48 g, 120 mmol) were added; Pd(PPh_3_)_4_ (5.78 g, 5.0 mmol) and K_2_CO_3_ (69.00 g, 500 mmol) were added as catalyst and base; 200 mL of dioxane and 50 mL of water were added as co-solvent. The reaction mixture was protected with Ar, and heated to reflux for 8 h. After the reaction was complete, the reaction mixture was cooled to room temperature and filtered to remove insoluble matter, concentrated under reduced pressure, and extracted three times with CH_2_Cl_2_. The combined organic phases were washed with brine, dried over anhydrous Na_2_SO_4_, and evaporated at reduced pressure. Column chromatography (PE/EA = 5:1) afforded **2** as a pale yellow oil (10.73 g, 80% yield). The spectral data are in agreement with the published ^1^H-NMR and ^13^C-NMR data [30].

#### 3.1.2. 2-Bromo-1-(4-(hydroxymethyl)phenyl)ethan-1-ol (**3**)

In a 1 L round-bottom flask, freshly prepared olefin **2** (10.72 g, 80 mmol) was dissolved in 400 mL of acetone and 100 mL H_2_O; then, NBS (17.09 g, 96 mmol) was added; and NH_4_OAc (0.12 g, 1.60 mmol) in 5 mL H_2_O was added dropwise. The reaction was stirred at room temperature for 4 h. When the reaction was complete, acetone was removed under vacuum, and the residue was extracted three times with CH_2_Cl_2_. The combined organic phases were washed with brine, dried over anhydrous Na_2_SO_4_, and evaporated at reduced pressure. Column chromatography (PE/EA = 5:1) produced **3** as a white solid (14.79 g, 80% yield, m.p. 70–72 °C). The spectral data are in agreement with the published ^1^H-NMR and ^13^C-NMR data [31].

#### 3.1.3. 2-(2-Hydroxy-2-(4-(hydroxymethyl)phenyl)ethyl)isoindoline-1,3-dione (**4**)

In a 500 mL round-bottom flask, the intermediate **3** (14.78 g, 64 mmol) was dissolved in 300 mL of anhydrous DMF, then potassium phthalimide (13.04 g, 70 mmol) was added. The reaction mixture was heated to 100 °C for 8 h until the reaction was complete. After insoluble matter was filtered, the filtrate was concentrated under vacuum. The residue was added to water and dispersed by ultrasonic, filtered and washed with water and petroleum ether, and dried to afford crude product **4** as a light pink solid (18.27 g, 96% yield, m.p. 158–160 °C).

#### 3.1.4. 2-(2-((tert-Butyldimethylsilyl)oxy)-2-(4-(((tert-butyldimethylsilyl)oxy)methyl)phenyl)ethyl)isoindoline-1,3-dione (**5**)

In a 500 mL round-bottom flask, intermediate **4** (18.14 g, 61 mmol) was dissolved in 300 mL of anhydrous DMF, imidazole (12.55 g, 184 mmol) was added, and then TBSCl (22.23 g, 147 mmol) was added portion-wise with ice bath. The reaction mixture was stirred and recovered to room temperature for 4 h and then quenched with water and extracted three times with ethyl acetate. The combined organic phases were washed with brine, dried over anhydrous Na_2_SO_4_, and evaporated at reduced pressure. Column chromatography (PE/EA = 10:1) afforded **5** as a white solid (22.94 g, 71% yield, m.p. 70–72 °C). 

#### 3.1.5. 2-((tert-Butyldimethylsilyl)oxy)-2-(4-(((tert-butyldimethylsilyl)oxy)methyl)phenyl)ethan-1-amine (**6**)

To a 500 mL round-bottom flask, the intermediate **5** (22.70 g, 43 mmol) was dissolved in 200 mL of methanol, and 5.3 mL of 80% hydrazine hydrate (87 mmol) was added. The mixture was stirred under reflux for 4 h until producing a large amount of white flocculent. Then, the insoluble material was removed by filtration, and the filtrate was condensed under vacuum. The residue was extracted three times with CH_2_Cl_2_; then, the combined organic phases were washed with brine, dried over anhydrous Na_2_SO_4_, and evaporated at reduced pressure. Column chromatography (DCM/MeOH/TEA = 20:1:0.2) afforded **6** as a light yellow oil (15.02 g, 87% yield). 

#### 3.1.6. *N*-(2-((tert-Butyldimethylsilyl)oxy)-2-(4-(((tert-butyldimethylsilyl)oxy)methyl)phenyl)ethyl)-2,6-difluorobenzamide (**7**)

To a 500 mL round-bottom flask, the intermediate **6** (14.90 g, 38 mmol) was dissolved in 200 mL of CH_2_Cl_2_, and 5.8 mL triethylamine (42 mmol) was added. Then, 2,6-difluorobenzoyl chloride (4.8 mL, 38 mmol) was slowly added under ice bath, and the reaction was stirred at room temperature for 4 h. The reaction was quenched with water and extracted with CH_2_Cl_2_, then the combined organic phases were washed with brine, dried over anhydrous Na_2_SO_4_, and evaporated at reduced pressure. Column chromatography (PE/EA = 5:1) afforded **7** as a colorless viscous oil (19.93 g, 98% yield). 

#### 3.1.7. 2,6-Difluoro-*N*-(2-hydroxy-2-(4-(hydroxymethyl)phenyl)ethyl)benzamide (**8**)

To a 500 mL round-bottom flask, the intermediate **7** (19.82 g, 37 mmol) was dissolved in 100 mL of THF, and TBFA solution (1 M in THF, 93 mL, 93 mmol) was added portion-wise with ice bath; then, the reaction was stirred at room temperature for 2 h until the reaction was complete. The mixture was quenched with saturated NH_4_Cl solution and extracted with CH_2_Cl_2_ three times. Then, the combined organic phases were washed with brine and then evaporated under vacuum. A large amount of water was added to the oily residue to precipitate a solid, which was collected and dried to afford crude product **8** as a light brown solid powder (8.23 g, 72% crude yield, m.p. 178–180 °C).

#### 3.1.8. *N*-(2-Chloro-2-(4-(chloromethyl)phenyl)ethyl)-2,6-difluorobenzamide (**9**)

To a 250 mL round-bottom flask, the intermediate **8** (8.20 g, 27 mmol) was dissolved in 150 mL of CH_2_Cl_2_. Pyridine (5.2 mL, 64 mmol) was added, and then SO_2_Cl_2_ (4.7 mL, 64 mmol) was added with a syringe under stirring. The mixture was heated to reflux for 6 h until the reaction was complete. The mixture was cooled, quenched with water, and extracted with CH_2_Cl_2_ three times. Then, the combined organic phases were washed with brine, dried over anhydrous Na_2_SO_4_, and evaporated at reduced pressure. Column chromatography (PE/EA = 3:1) afforded **9** as a light yellow solid (7.28 g, 79% yield, m.p. 119–121 °C).

#### 3.1.9. 5-(4-(Chloromethyl)phenyl)-2-(2,6-difluorophenyl)-4,5-dihydrooxazole (**10**)

To a 250 mL round-bottom flask, the intermediate **9** (7.20 g, 21 mmol) was dissolved in 100 mL of MeCN; then, NaOH (1.01 g, 25 mmol) was added, and the mixture was stirred for 15 min at room temperature until the precipitation of a solid. When the reaction was complete, it was quenched with water and extracted with ethyl acetate three times; then, the combined organic phases were washed with brine, dried over anhydrous Na_2_SO_4_, and evaporated at reduced pressure. Column chromatography (PE/EA = 3:1) afforded **10** as a white solid (5.73 g, 88% yield, m.p. 83–85 °C).

#### 3.1.10. Preparation of Target Compound 2-(4-(2-(2,6-difluorophenyl)-4,5-dihydrooxazol-5-yl)benzyl)isoindoline-1,3-dione (**11a**) 

To a 25 mL round-bottom flask, the intermediate **10** (308 mg, 1.0 mmol) was dissolved in 5.0 mL of DMF, and potassium phthalimide (222 mg, 1.2 mmol) was added under stirring. The reaction mixture was heated to 80 °C for 4 h. After adding 50 mL of water, the mixture was extracted with ethyl acetate three times; then, the combined organic phases were washed with brine, dried over anhydrous Na_2_SO_4_, and evaporated at reduced pressure. Column chromatography (PE/EA = 1:1) afforded **11a** as a colorless oil.

#### 3.1.11. Preparation of Target Compounds **11b**–**11g** and **11t**–**11x**

To a 25 mL glass bottle, the nucleophile reagent (1.1 mmol) was dissolved in 2.0 mL of DMF, and NaH (60 mg, 1.5 mmol) was added under stirring. After stirring for 30 min, the intermediate **10** (308 mg, 1.0 mmol) in 3.0 mL of DMF was added dropwise, and the reaction was stirred for another 4 h at room temperature. Then, the post-treatment of the reaction followed the operation of **11a**.

#### 3.1.12. Preparation of Target Compounds **11h**–**11m**

To an 8 mL glass vial, the intermediate **10** (308 mg, 1.0 mmol) was dissolved in 3.0 mL MeCN, and then the corresponding alcohol (1.2 mmol), NaOH (48 mg, 1.2 mmol), and KI (199 mg, 1.2 mmol) were successfully added. The mixture was protected with Ar and heated to reflux for 4 h. Then, after cooling, the post-treatment followed the operation of **11a**.

#### 3.1.13. Preparation of Target Compounds **11n**–**11s**

The synthesis procedure was the same as that for **11h**–**11m**, except that NaOH was changed to K_2_CO_3_ (166 mg, 1.2 mmol).

#### 3.1.14. Preparation of Target Compound **11y**

To an 8 mL glass vial, the intermediate **10** (308 mg, 1.0 mmol) was dissolved in 3 mL of MeCN, then sodium *p*-toluenesulfinate (214 mg, 1.2 mmol) and KI (199 mg, 1.2 mmol) were added. Then, the mixture was stirred under reflux for 4 h. After cooling, the post-treatment followed the operation of **11a**.

### 3.2. Acaricidal Activity Evaluation

The acaricidal activities against mite (*Tetranychus cinnabarinus*) eggs and larvae of the target compounds **11a**–**11y**, intermediates **8**–**10**, control compounds **12**, and etoxazole were assayed. Detailed procedure is described in the Supplementary Materials. 

## 4. Conclusions

In summary, considering the structural similarity between 2,4-diphenyloxazoline and 2,5-diphenyloxazoline, we designed and synthesized a series of 2,5-diphenyloxazolines through a scaffold hopping/ring equivalent and intermediate derivatization strategy and studied their acaricidal activity. The results showed that most of the 2,5-diphenyloxazolines exhibited 100% acaricidal activity at a concentration of 100 mg/L, and half of the compounds had 100% acaricidal activity against mite larvae. However, the overall activity level was lower than that of the corresponding 2,4-diphenyloxazolines with identical substituents in the 4-phenyl group. We speculate that the main reason for the difference in binding ability is due to the different spatial orientations of substituents when 2,4-diphenyloxazoline and 2,5-diphenyloxazoline bind to biological targets. Although this work is helpful in understanding the internal structure of target binding pockets, the structure of 2,5-diphenyloxazolines as an acaricide is not worth further research, so the *N*-phenylethylbenzamide-type structure and chiral 2,4-diphenyloxazolines will be our future research directions.

## Figures and Tables

**Table 1:** Mortalities of compounds **8**~**12** against mite eggs and larvae *^a^*.

	Mortality (%) against Eggs			Mortality (%) against Larvae		
Compound	100 mg/L	10 mg/L	5 mg/L	100 mg/L	10 mg/L	5 mg/L
**11a**	100	68.7±9.8	0	100	61.7 ± 12.8	33.7 ± 10.2
**11b**	100	20.0 ± 2.2	-	100	54.3 ± 7.2	31.0 ± 6.2
**11c**	100	76.8 ± 4.9	14.3 ± 3.4	100	78.4 ± 6.0	55.4 ± 2.6
**11d**	100	91.5 ± 2.9	6.6 ± 2.5	68.4 ± 7.3	30.4 ± 9.4	-
**11e**	100	86.8 ± 6.5	0	61.7 ± 12.2	23.7 ± 1.8	-
**11f**	100	28.6 ± 13	-	100	54.7 ± 5.0	26.7 ± 5.7
**11g**	100	20.4 ± 0.3	-	100	78.3 ± 18.2	42.5 ± 15.7
**11i**	100	49.5 ± 0.7	-	100	72.0 ± 3.6	41.4 ± 9.7
**11j**	100	0.0	-	100	83.1 ± 9.2	59.7 ± 15.7
**11k**	100	49.6 ± 3.5	-	100	74.2 ± 7.4	52.0 ± 2.0
**11l**	100	100	15.3 ± 5.0	100	52.3 ± 3.4	38.6 ± 9.2
**11n**	100	20 ± 0.5	-	100	63.0 ± 8.3	47.0 ± 1.5
**11o**	100	100	16.3 ± 5.3	100	61.1 ± 12.1	44.1 ± 23.3
**11p**	0	0	-	91.8 ± 6.8	36.8 ± 3.5	-
**11q**	100	100	22.3 ± 11.6	100	56.6 ± 7.7	42.6 ± 6.9
**11r**	100	50 ± 4.2	-	78.5 ± 2.0	36.4 ± 10.2	-
**11s**	0	0	-	73.2 ± 1.8	44.8 ± 10.6	-
**11t**	100	100	0	88.2 ± 6.8	54.7 ± 5.0	20.6 ± 5.4
**11u**	100	90.6 ± 8.3	0	94.2 ± 5.5	52.9 ± 9.0	36.7 ± 2.3
**11v**	100	59.0 ± 0.9	0	91.6 ± 8.4	36.2 ± 2.1	-
**11w**	0	0	-	83.7 ± 1.8	39.4 ± 6.1	-
**11x**	91.4 ± 8.8	74.8 ± 5.6	0	97.0 ± 4.4	45.9 ± 15.9	-
**11y**	100	100	0	100	81.8 ± 10.5	58.9 ± 2.5
**8**	100	79.8 ± 12.1	0	100	79.5 ± 6.4	59.8 ± 5.2
**9**	100	100	0	100	100	100
**10**	100	77.4 ± 5.0	0	100	100	76.2 ± 12.6
**12a**	100	100	100	100	96.6 ± 3.3	65.4 ± 3.9
**12b**	100	100	100	100	75.3 ± 2.1	49.6 ± 2.1
**12i**	100	100	100	100	60.8 ± 1.7	36.9 ± 3.1
**12o**	100	100	100	100	74.4 ± 9.2	50.0 ± 3.4
**12r**	100	100	100	100	94.7 ± 5.2	73.6 ± 11.6
**12s**	100	100	100	100	93.8 ± 5.8	77.8 ± 9.9
**12t**	100	100	100	95.3 ± 4.3	77.6 ± 2.3	47.1 ± 2.4
**12u**	100	100	100	100	73.5 ± 7.9	45.8 ± 8.9
**12v**	100	100	100	100	69.0 ± 13.5	52.6 ± 3.2
**etoxazole**	100	100	100	100	100	92.0 ± 7.7

*^a^* The activity data are presented as mean ± SD of three replicates. “-” means no test.

**Table 2:** Mortalities of **9** and etoxazole against mite larvae *^a^*.

Compound	5 mg/L	2.5 mg/L	1.25 mg/L	0.63 mg/L	0.31 mg/L	0.16 mg/L
**9**	100	100	100	35.6 ± 10.4	17.2 ± 13.5	-
**etoxazole**	92.0	86.3 ± 5.8	85.7 ± 5.0	61.2 ± 12.8	53.9 ± 19.3	37.1 ± 10.2

*^a^* The activity data are presented in mean ± SD of three replicates. “-” means “no test”.

## Data Availability

The authors confirm that the data supporting the findings of this study are available within the article and its supplementary materials.

## Supplementary Materials

The following supporting information can be downloaded at: www.mdpi.com/xxx/s1.The physical–chemical data, NMR spectra, and HRMS spectra for intermediates **4**–**10** and target compounds **11a**–**11y**, detailed bioassay methods for acaricidal activity.
