# Supplementary material for: Design, Synthesis, and Acaricidal Activity of 2,5-Diphenyl-1,3-oxazoline Compounds
Source: Molecules. 2024 Aug 31;29(17):4149. doi: 10.3390/molecules29174149 (PMC11396784; doi:10.3390/molecules29174149)
Supplement: Supplementary file 1 [file molecules-29-04149-s001.zip › molecules-3183300-supplementary.pdf]

Supporting Information

Design, Synthesis, and Acaricidal Activity of 2,5-Diphenyl-1,3-Oxazoline Compounds

Contents

1. Synthesis of key intermediates...S2

2. General procedures for preparing target compounds **11a**–**11y**................................S7

3. Characterization of intermediates target compounds **11a**–**11y**................................S8

4. ^{1}H and ^{13}C NMR spectrums of intermediates **4-10** and target compounds **11a**–**11y**
....S18

5. HRMS spectrums of intermediates **4-10** and target compounds **11a**–**11y**.............S50

6. Bioassay methods…………..………………………………………………….S66

7. References………...……………………………………...………………….S67

1. Synthesis of key intermediates

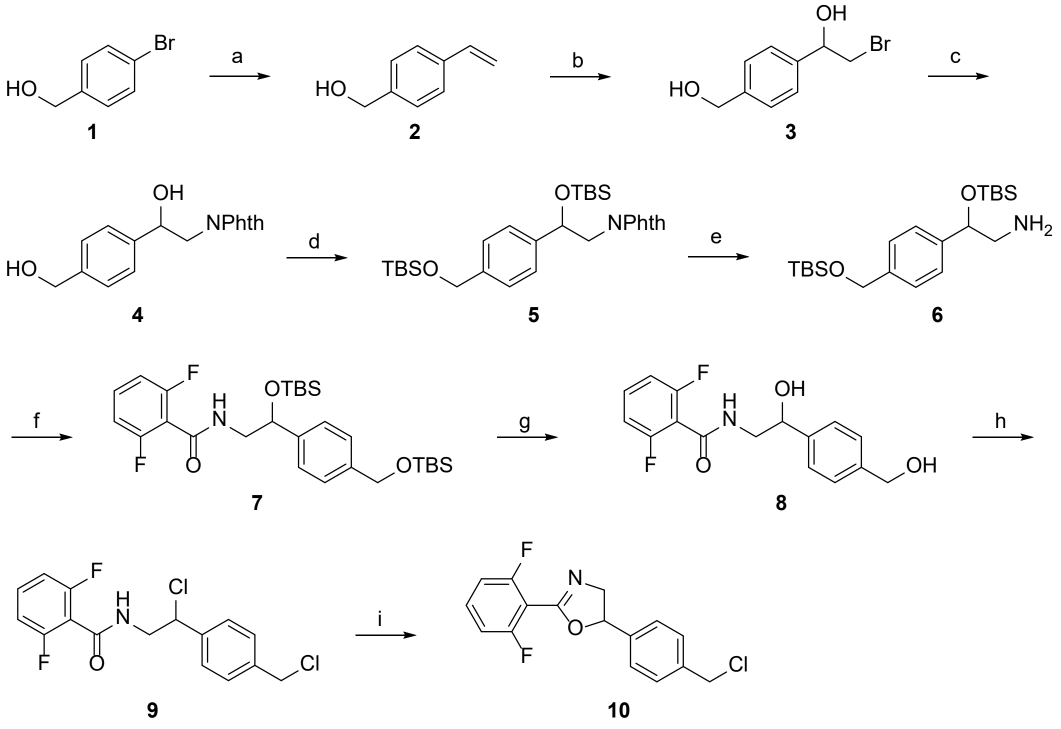

Reagents and conditions: (a) Vinylboronic acid pinacol ester, $Pd(PPh_3)_4$, K_2CO_3, dioxane/H_2O = 4:1, reflux, 80%; (b) NBS, NH_4OAc(2 mmol%), acetone/H_2O = 4:1, 0 °C, 80%; (c) potassium phthalimide, DMF, 100 °C, 96%; (d) TBSCl, imidazole, DMF, 71%; (e) $N_2H_4{\cdot}H_2O$, MeOH, reflux, 87%; (f) 2,6-difluorobenzoyl chloride, triethylamine, CH_2Cl_2, 98%; (g) TBAF(1M THF), THF, 72%; (h) $SOCl_2$, Pyridine, CH_2Cl_2, 40 °C, 79%; (i) NaOH, MeCN, 88%.

Reagents were purchased from commercial sources and were used as received. ^{1}H and ^{13}C Nuclear Magnetic Resonance (NMR) spectra were recorded on Bruker Avance 400 Ultrashield NMR spectrometers. Chemical shifts (δ) were given in parts per million (ppm) and were measured downfield from internal tetramethylsilane. High-resolution mass spectrometry (HRMS) data were obtained on an FTICR-MS instrument (Ionspec 7.0 T). The melting points were determined on an X-4 microscope melting point apparatus and are uncorrected. Conversion was monitored by thin layer chromatography (TLC). Flash column chromatography was performed over silica gel (100-200 mesh).

1.1 Preparation of intermediate **2**

In a 500 mL round bottom flask, *p*-bromobenzyl alcohol (18.70 g, 100 mmol, 1.0

equiv) and vinylboronic acid pinacol ester (18.48 g, 120 mmol, 1.2 equiv) were added, then $Pd(PPh_3)_4$ (5.78 g, 5 mmol, 0.05 equiv) and K_2CO_3 (69 g, 500 mmol, 5 equiv) were added as catalyst and base, 200 mL dioxane and 50 mL water were added as co-solvent, protected with Ar, and heated to reflux for 8 hours. After the reaction was complete, the reaction mixture was cooled to room temperature and filtered to remove insoluble matter, concentrated under reduced pressure, and extracted three times with CH_2Cl_2. The combined organic phases were washed with brine, dried over anhydrous Na_2SO_4, and evaporated at reduced pressure. Column chromatography (PE/EA = 5:1) afforded **2** as a pale yellow oil (10.73 g, 80% yield). ^{1}H NMR (400 MHz, $CDCl_3$) δ 7.40 (d, J = 8.1 Hz, 2H), 7.31 (d, J = 8.2 Hz, 2H), 6.71 (dd, J = 17.6, 10.9 Hz, 1H), 5.74 (d, J = 17.6 Hz, 1H), 5.24 (d, J = 10.9 Hz, 1H), 4.67 (s, 2H), 1.76 (s, 1H). ^{13}C NMR (100 MHz, $CDCl_3$) δ 140.6, 137.2, 136.6, 127.3, 126.5, 114.0, 65.2. The spectral data are in agreement with the published ^{1}H-NMR and ^{13}H-NMR data [S1].

1.2 Preparation of intermediate **3**

In a 1 L round bottom flask, *freshly prepared* olefin **2** was dissolved in 400 mL acetone and 100 mL H_2O, then NBS (17.09 g, 96.00 mmol, 1.2 equiv) was added and NH_4OAc (123 mg, 1.60 mmol, 0.02 equiv) in 5 mL H_2O was added dropwise. The reaction was stirred at room temperature for 4 hours. When reaction was complete, acetone was removed under vacuum and the residue was extracted three times with CH_2Cl_2. The combined organic phases were washed with brine, dried over anhydrous Na_2SO_4, and evaporated at reduced pressure. Column chromatography (PE/EA = 5:1) afforded **3** as a white solid (14.79 g, 80% yield, m.p. 70–72 °C). ^{1}H NMR (400 MHz, $CDCl_3$) δ 7.36 (s, 4H), 4.92 (dd, J = 8.8, 3.4 Hz, 1H), 4.68 (s, 2H), 3.62 (dd, J = 10.4, 3.4 Hz, 1H), 3.52 (dd, J = 10.5, 8.8 Hz, 1H), 2.79 (s, 1H), 1.94 (s, 1H). ^{13}C NMR (100 MHz, $CDCl_3$) δ 141.3, 139.8, 127.4, 126.3, 73.7, 65.0, 40.2. The spectral data are in agreement with the published ^{1}H-NMR and ^{13}H-NMR data [S2].

1.3 Preparation of intermediate **4**

In a 500 mL round bottom flask, the intermediate **3** was dissolved in 300 mL anhydrous DMF, then potassium phthalimide (13.04 g, 70.40 mmol, 1.1 equiv) was added. The reaction mixture was heated to 100 °C for 8 hours until the reaction was complete. After insoluble matter was filtered, the filtrate was concentrated under vacuum. The residue was added water and dispersed by ultrasonic, then filtered and washed with water and petroleum ether, dried to afford crude product **4** as a light pink solid (18.27 g, 96% yield, m.p. 158 – 160 °C). ^{1}H NMR (400 MHz, $CDCl_3$) δ 7.86 –

7.82 (m, 4H), 7.33 (d, *J* = 8.1 Hz, 2H), 7.29 (d, *J* = 8.0 Hz, 2H), 5.60 (s, 1H), 4.92 (dd, *J* = 8.9, 4.7 Hz, 1H), 4.49 (s, 2H), 3.76 (dd, *J* = 13.6, 8.9 Hz, 1H), 3.63 (dd, *J* = 13.6, 4.7 Hz, 1H). ^{13}C NMR (100 MHz, $CDCl_3$) δ 167.8, 141.7, 140.8, 134.4, 131.7, 126.3, 125.7, 123.0, 69.3, 62.7, 45.5. HRMS(ESI): m/z calcd for $C_{17}H_{15}NNaO_4^+$ $[M+Na]^+$ 320.0893, found, 320.0895.

1.4 Preparation of intermediate **5**

In a 500 mL round bottom flask, the intermediate **4** was dissolved in 300 mL anhydrous DMF, then imidazole (12.55 g, 184.32 mmol, 3.0 equiv) was added, TBSCl (22.23 g, 147.46 mmol, 2.4 equiv) was added portionwise with ice bath. The reaction mixture was stirred and recovered to room temperature for 4 hours, and then quenched with water and extracted three times with ethyl acetate. The combined organic phases were washed with brine, dried over anhydrous Na_2SO_4, and evaporated at reduced pressure. Column chromatography (PE/EA = 10:1) afforded **5** as a white solid (22.94 g, 71% yield, m.p. 70–72 °C). ^{1}H NMR (400 MHz, $CDCl_3$) δ 7.85 (dd, *J* = 5.5, 3.1 Hz, 2H), 7.71 (dd, *J* = 5.6, 3.0 Hz, 2H), 7.39 (d, *J* = 7.8 Hz, 2H), 7.29 (d, *J* = 7.9 Hz, 2H), 5.07 (dd, *J* = 9.3, 4.1 Hz, 1H), 4.74 (s, 2H), 3.96 (dd, *J* = 13.6, 9.4 Hz, 1H), 3.65 (dd, *J* = 13.6, 4.0 Hz, 1H), 0.93 (s, 9H), 0.73 (s, 9H), 0.08 (s, 6H), -0.23 (s, 3H), -0.26 (s, 3H). ^{13}C NMR (100 MHz, $CDCl_3$) δ 168.4, 141.2, 140.7, 134.0, 132.3, 126.3, 126.2, 123.0, 72.0, 65.0, 46.4, 26.1, 25.7, 18.6, 17.9, -4.8, -5.1, -5.2. HRMS(ESI): m/z calcd for $C_{29}H_{44}NO_4Si_2^+$ $[M+H]^+$ 526.2803, found, 526.2803.

1.5 Preparation of intermediate **6**

To a 500 mL round bottom flask, the intermediate **5** was dissolved in 200 mL methanol, and 5.25 mL of 80% hydrazine hydrate (87.24 mmol, 2.0 equiv) was added. The mixture was stirred under reflux for 4 hours until producing a large amount of white flocculent. Then the insoluble material was removed by filtration, and the filtrate was condensed under vacuum. The residue was extracted three times with CH_2Cl_2, then the combined organic phases were washed with brine, dried over anhydrous Na_2SO_4, and evaporated at reduced pressure. Column chromatography (DCM/MeOH/TEA = 20:1:0.2) afforded **6** as a light yellow oil (15.02 g, 87% yield). ^{1}H NMR (400 MHz, $CDCl_3$) δ 7.26 (s, 4H), 4.73 (s, 2H), 4.64 (t, *J* = 5.3 Hz, 1H), 2.80 (d, *J* = 5.3 Hz, 2H), 1.32 (s, 2H), 0.94 (s, 9H), 0.91 (s, 9H), 0.10 (s, 6H), 0.05 (s, 3H), -0.11 (s, 3H). ^{13}C NMR (100 MHz, $CDCl_3$) δ 141.88, 140.53, 126.11, 126.03, 76.71, 64.98, 51.26, 26.12, 26.02, 18.58, 18.38, -4.43, -4.78, -5.09. HRMS(ESI): m/z calcd for $C_{21}H_{42}NO_2Si_2^+$ $[M+H]^+$ 396.2749, found, 396.2740.

1.6 Preparation of intermediate **7**

To a 500 mL round bottom flask, the intermediate **6** was dissolved in 200 mL CH_2Cl_2, and 5.80 mL triethylamine (41.75 mmol, 1.1 equiv) was added. Then, 2,6-difluorobenzoyl chloride (4.77 mL, 37.95 mmol, 1.0 equiv) was slowly added under ice bath and the reaction was stirred at room temperature for 4 hours. The reaction was quenched with water and extracted with CH_2Cl_2, then the combined organic phases were washed with brine, dried over anhydrous Na_2SO_4, and evaporated at reduced pressure. Column chromatography (PE/EA = 5:1) afforded **7** as a colorless viscous oil (19.93 g, 98% yield). To a 500 mL round bottom flask, the intermediate **6** was dissolved in 200 mL CH_2Cl_2, and 5.80 mL triethylamine (41.75 mmol, 1.1 equiv) was added. Then, 2,6-difluorobenzoyl chloride (4.77 mL, 37.95 mmol, 1.0 equiv) was slowly added under ice bath and the reaction was stirred at room temperature for 4 hours. The reaction was quenched with water and extracted with CH_2Cl_2, then the combined organic phases were washed with brine, dried over anhydrous Na_2SO_4, and evaporated at reduced pressure. Column chromatography (PE/EA = 5:1) afforded **7** as a colorless viscous oil (19.93 g, 98% yield). ^{1}H NMR (400 MHz, $CDCl_3$) δ 7.38 – 7.28 (m, 5H), 6.94 (t, J = 8.3 Hz, 2H), 6.20 (s, 1H), 4.94 – 4.86 (m, 1H), 4.74 (s, 2H), 3.85 – 3.74 (m, 1H), 3.51 – 3.40 (m, 1H), 0.94 (s, 9H), 0.87 (s, 9H), 0.09 (s, 6H), 0.04 (s, 3H), -0.12 (s, 3H). ^{13}C NMR (100 MHz, $CDCl_3$) δ 160.4, 160.2 (dd, J = 252.6, 7.1 Hz), 141.1, 140.8, 131.8 (t, J = 10.3 Hz), 126.2, 126.2, 114.4 (t, J = 19.5 Hz), 112.4 – 112.0 (m), 73.4, 65.0, 48.3, 26.1, 25.9, 18.6, 18.3, -4.5, -5.0, -5.1. HRMS(ESI): m/z calcd for $C_{28}H_{44}F_2NO_3Si_2^+$ $[M+H]^+$ 536.2822, found, 536.2815.

1.7 Preparation of intermediate **8**

To a 500 mL round bottom flask, the intermediate **7** was dissolved in 100 mL THF, and TBFA solution (1M in THF, 93 mL, 93 mmol, 2.5 equiv) was added portionwise with ice bath, then the reaction was stirred at room temperature for 2 hours until the reaction was complete. The mixture was quenched with saturated NH_4Cl solution and extracted with CH_2Cl_2 for three times. Then the combined organic phases were washed with brine and then evaporated under vacuum. The oily residue was added a large amount of water to precipitate solid, which was collected and dried to afford crude product **8** as a light brown solid powder (8.23 g, 72% crude yield, m.p. 178–180 °C). ^{1}H NMR (400 MHz, $CDCl_3$) δ 8.77 (t, J = 5.7 Hz, 1H), 7.54 – 7.42 (m, 1H), 7.33 (d, J = 7.6 Hz, 2H), 7.28 (d, J = 7.9 Hz, 2H), 7.13 (t, J = 7.9 Hz, 2H), 5.46 (d, J = 4.5 Hz, 1H), 5.18 – 5.11 (m, 1H), 4.74 – 4.65 (m, 1H), 4.48 (d, J = 5.7 Hz, 2H), 3.48 – 3.34 (m,

2H). ^{13}C NMR (100 MHz, $CDCl_3$) δ 159.8, 158.8 (dd, J = 248.4, 8.1 Hz), 141.9, 141.4, 131.3 (t, J = 9.9 Hz), 126.1, 125.9, 115.6 (t, J = 23.2 Hz), 112.0 – 111.5 (m), 71.0, 62.7, 47.3. HRMS(ESI): m/z calcd for $C_{16}H_{16}F_2NO_3^+$ [M+H]$^+$ 308.1093, found, 308.1091.

1.8 Preparation of intermediate **9**

To a 250 mL round bottom flask the intermediate **8** was dissolved in 150 mL CH_2Cl_2. Pyridine (5.17 mL, 64.27 mmol, 2.4 equiv) was added and then SO_2Cl_2 (4.67 mL, 64.27 mmol, 2.4 equiv) was added with a syringe under stirring. The mixture was heated to reflux for 6 hours until the reaction was complete, cooled, quenched with water, and extracted with CH_2Cl_2 for three times. Then, the combined organic phases were washed with brine, dried over anhydrous Na_2SO_4, and evaporated at reduced pressure. Column chromatography (PE/EA = 3:1) afforded **9** as a light yellow solid (7.28 g, 79% yield, m.p. 119–121 °C). ^{1}H NMR (400 MHz, $CDCl_3$) δ 7.46 (d, J = 8.3 Hz, 2H), 7.42 (d, J = 8.3 Hz, 2H), 7.40 – 7.33 (m, 1H), 6.95 (t, J = 8.2 Hz, 2H), 6.36 (s, 1H), 5.16 (dd, J = 8.5, 5.4 Hz, 1H), 4.59 (s, 2H), 4.12 – 4.03 (m, 1H), 3.86 – 3.78 (m, 1H). ^{13}C NMR (100 MHz, $CDCl_3$) δ 160.7, 160.2 (dd, J = 253.0, 6.6 Hz), 138.8, 138.3, 132.2 (t, J = 10.3 Hz), 129.2, 127.8, 113.8 (t, J = 19.7 Hz), 112.4 – 112.1 (m), 61.1, 47.6, 45.7. HRMS(ESI): m/z calcd for $C_{16}H_{14}Cl_2F_2NO^+$ [M+H]$^+$ 344.0415, found, 344.0415.

1.9 Preparation of intermediate **10**

To a 250 mL round bottom flask, the intermediate **9** was dissolved in 100 mL MeCN, then NaOH (1.01 g, 5.39 mmol, 1.2 equiv) was added and the mixture was stirred for 15 min at room temperature until the precipitation of solid. When the reaction was complete, it was quenched with water and extracted with ethyl acetate for three times, then the combined organic phases were washed with brine, dried over anhydrous Na_2SO_4, and evaporated at reduced pressure. Column chromatography (PE/EA = 3:1) afforded **10** as a white solid (5.73 g, 88% yield, m.p. 83–85 °C). ^{1}H NMR (400 MHz, $CDCl_3$) δ 7.47 – 7.34 (m, 5H), 6.99 (t, J = 8.3 Hz, 2H), 5.69 (dd, J = 10.4, 8.0 Hz, 1H), 4.58 (s, 2H), 4.54 (dd, J = 14.9, 10.4 Hz, 1H), 4.02 (dd, J = 14.9, 8.0 Hz, 1H). ^{13}C NMR (100 MHz, $CDCl_3$) δ 161.3 (dd, J = 256.2, 6.2 Hz), 156.6, 141.0, 137.7, 132.5 (t, J = 10.4 Hz), 129.2, 126.2, 112.2 – 111.9 (m), 107.3 (t, J = 17.6 Hz), 80.7, 63.4, 45.9. HRMS(ESI): m/z calcd for $C_{16}H_{13}ClF_2NO^+$ [M+H]$^+$ 308.0648, found, 308.0650.

2. General procedures for preparing target compounds 11a–11y

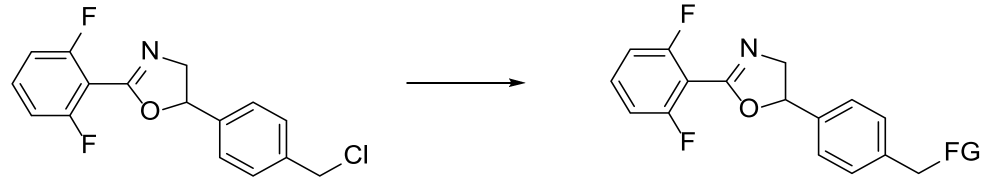

10 **11a-11y**

2.1 Preparation of target compound **11a**

To a 25 mL round bottom flask, the intermediate **10** (308 mg, 1.0 mmol, 1.0 equiv) was resolved in 5 mL DMF, and potassium phthalimide (222 mg, 1.2 mmol, 1.2 equiv) was added under stirring. The reaction mixture was heated to 80 °C for 4 hours. After adding 50 mL water, the mixture was extracted with ethyl acetate for three times, then the combined organic phases were washed with brine, dried over anhydrous Na_2SO_4, and evaporated under vacuum. Column chromatography (PE/EA = 1:1) afforded **11a** as a colorless oil.

2.2 Preparation of target compounds **11b–11g** and **11t–11x**

To a 25 mL glass bottle, the nucleophile reagent (1.1 mmol, 1.1 equiv) was dissolved in 2 mL DMF, and NaH (60 mg, 1.5 mmol, 1.5 equiv) was added under stirring. After stirring for 30 min, the intermediate **10** (308 mg, 1.0 mmol, 1.0 equiv) in 3 mL DMF was added dropwise, and the reaction was stirred for another 4 hours at room temperature. Then, the post-treatment of the reaction follows the operation of **11a**.

2.3 Preparation of target compounds **11h–11m**

To an 8 mL glass vial the intermediate **10** (308 mg, 1.0 mmol, 1.0 equiv) was dissolved in 3 mL MeCN, and then the corresponding alcohol (1.2 mmol, 1.2 equiv), NaOH (48 mg, 1.2 mmol, 1.2 equiv) and KI (199 mg, 1.2 mmol, 1.2 equiv) was successfully added. The mixture was protected with Ar and heated to reflux for 4 hours, Then, after cooling, the post-treatment follows the operation of **11a**.

2.4 Preparation of target compounds **11n–11s**

The synthetic procedure is according to the procedure for **11h–11m** except that NaOH was changed to K_2CO_3 (166 mg, 1.2 mmol, 1.2 equiv).

2.5 Preparation of target compounds **11y**

To an 8 mL glass vial the intermediate **10** (308 mg, 1.0 mmol, 1.0 equiv) was dissolved in 3

mL MeCN, then sodium *p*-toluenesulfinate (214 mg, 1.2 mmol, 1.2 equiv) and KI (199 mg, 1.2 mmol, 1.2 equiv) were added. Then the mixture was stirred under reflux for 4 hours. After cooling, the post-treatment follows the operation of **11a**.

3. Characterization of target compounds 11a–11y

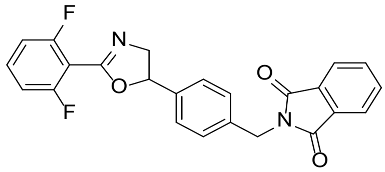

2-(4-(2-(2,6-difluorophenyl)-4,5-dihydrooxazol-5-yl)benzyl)isoindoline-1,3-dione (**11a**), colorless oil, 92% yield. ^{1}H NMR (400 MHz, $CDCl_3$) δ 7.83 (dd, J = 5.5, 2.9 Hz, 2H), 7.70 (dd, J = 5.5, 2.9 Hz, 2H), 7.47 (d, J = 8.1 Hz, 2H), 7.44 – 7.37 (m, 1H), 7.35 (d, J = 8.2 Hz, 2H), 6.97 (t, J = 8.3 Hz, 2H), 5.66 (dd, J = 10.4, 8.0 Hz, 1H), 4.85 (s, 2H), 4.50 (dd, J = 14.9, 10.4 Hz, 1H), 3.98 (dd, J = 14.9, 8.0 Hz, 1H). ^{13}C NMR (100 MHz, $CDCl_3$) δ 168.1, 161.3 (dd, J = 256.1, 6.0 Hz), 156.6 (t, J = 2.0 Hz), 140.4, 136.7, 134.1, 132.4 (t, J = 10.4 Hz), 132.2, 129.2, 126.2, 123.5, 112.2 – 111.9 (m), 107.3 (t, J = 17.7 Hz), 80.8, 63.4, 41.3. HRMS (ESI): m/z calcd for $C_{24}H_{17}F_2N_2O_3^+$ $[M+H]^+$ 419.1202, found 419.1206.

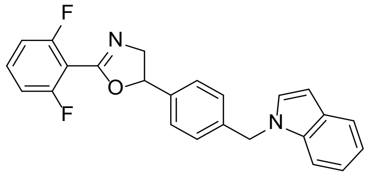

5-(4-((1*H*-indol-1-yl)methyl)phenyl)-2-(2,6-difluorophenyl)-4,5-dihydrooxazole (**11b**), orange oil, 75% yield. ^{1}H NMR (400 MHz, $CDCl_3$) δ 7.65 (d, J = 7.6 Hz, 1H), 7.37 (tt, J = 8.4, 6.2 Hz, 1H), 7.29 (d, J = 8.2 Hz, 2H), 7.25 (d, J = 8.1 Hz, 1H), 7.20 – 7.12 (m, 1H), 7.13 – 7.08 (m, 4H), 6.95 (t, J = 8.3 Hz, 2H), 6.55 (d, J = 2.6 Hz, 1H), 5.62 (dd, J = 10.4, 8.0 Hz, 1H), 5.30 (s, 2H), 4.48 (dd, J = 14.9, 10.4 Hz, 1H), 3.97 (dd, J = 14.9, 8.0 Hz, 1H). ^{13}C NMR (100 MHz, $CDCl_3$) δ 161.3 (dd, J = 256.1, 6.0 Hz), 156.6 (t, J = 2.1 Hz), 140.1, 138.0, 136.3, 132.4 (t, J = 10.4 Hz), 128.8, 128.3, 127.3, 126.3, 121.9, 121.1, 119.7, 112.2 – 111.9 (m), 109.7, 107.3 (t, J = 17.7 Hz), 101.9, 80.8, 63.4, 49.8. HRMS (ESI): m/z calcd for $C_{24}H_{19}F_2N_2O^+$ $[M+H]^+$ 389.1460, found 389.1454.

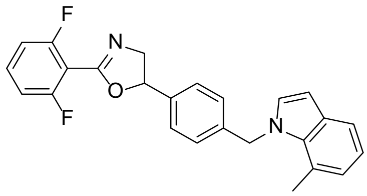

2-(2,6-difluorophenyl)-5-(4-((7-methyl-1*H*-indol-1-yl)methyl)phenyl)-4,5-dihydrooxazole (**11c**), yellow oil, 70% yield. ^{1}H NMR (400 MHz, $CDCl_3$) δ 7.50 (d, J = 7.9 Hz, 1H), 7.37 (tt, J = 8.3, 6.2 Hz, 1H), 7.27 (d, J = 8.0 Hz, 2H), 7.04 – 6.85 (m, 7H), 6.55 (d, J = 3.1 Hz, 1H), 5.62 (dd, J = 10.4, 8.0 Hz, 1H), 5.55 (s, 2H), 4.48 (dd, J = 14.9, 10.4 Hz, 1H), 3.97 (dd, J = 14.9, 8.0 Hz, 1H), 2.50 (s, 3H). ^{13}C NMR (100 MHz, $CDCl_3$) δ 161.3 (dd, J = 256.0, 6.1 Hz), 156.6 (t, J = 2.1 Hz), 140.2, 139.9, 135.1, 132.4 (t, J = 10.5 Hz), 130.2, 129.9, 126.3, 126.0, 124.8, 121.0, 120.0, 119.3, 112.2 – 111.9 (m), 107.3 (t, J = 17.7 Hz), 102.3, 80.9, 63.4, 52.1, 19.6. HRMS (ESI): m/z calcd for $C_{25}H_{21}F_2N_2O^+$ $[M+H]^+$ 403.1616, found 403.1621.

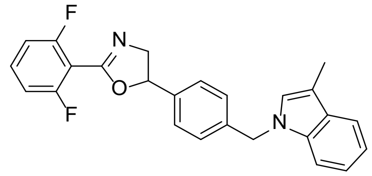

2-(2,6-difluorophenyl)-5-(4-((3-methyl-1*H*-indol-1-yl)methyl)phenyl)-4,5-dihydrooxazole (**11d**), yellow oil, 70% yield. ^{1}H NMR (400 MHz, $CDCl_3$) δ 7.65 (d, J = 7.6 Hz, 1H), 7.43 (tt, J = 8.5, 6.2 Hz, 1H), 7.35 (d, J = 8.2 Hz, 2H), 7.31 – 7.25 (m, 2H), 7.20 – 7.15 (m, 3H), 7.01 (t, J = 8.3 Hz, 2H), 6.93 (d, J = 1.2 Hz, 1H), 5.68 (dd, J = 10.4, 8.0 Hz, 1H), 5.29 (s, 2H), 4.54 (dd, J = 14.9, 10.4 Hz, 1H), 4.03 (dd, J = 14.9, 8.0 Hz, 1H), 2.39 (d, J = 1.1 Hz, 3H). ^{13}C NMR (100 MHz, $CDCl_3$) δ 161.3 (dd, J = 256.0, 5.9 Hz), 156.6, 140.0, 138.3, 136.7, 132.4 (t, J = 10.4 Hz), 129.1, 127.4, 126.2, 125.9, 121.8, 119.2, 119.0, 112.0 (d, J = 23.2 Hz), 111.1, 109.5, 107.3 (t, J = 17.4 Hz), 80.9, 63.4, 49.5, 9.7. HRMS (ESI): m/z calcd for $C_{25}H_{21}F_2N_2O^+$ $[M+H]^+$ 403.1616, found, 403.1618.

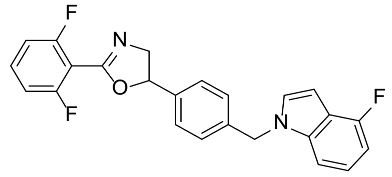

2-(2,6-difluorophenyl)-5-(4-((4-fluoro-1*H*-indol-1-yl)methyl)phenyl)-4,5-dihydrooxazole (**11e**), yellow oil, 85% yield. ^{1}H NMR (400 MHz, $CDCl_3$) δ 7.38 (tt, J = 8.5, 6.2 Hz, 1H), 7.31 (d, J = 8.2 Hz, 2H), 7.29 – 7.23 (m, 1H), 7.17 – 7.06 (m, 4H), 6.96 (t, J = 8.3 Hz, 2H), 6.89 (td, J = 9.1, 2.5 Hz, 1H), 6.50 (d, J = 3.1 Hz, 1H), 5.63 (dd, J = 10.4, 8.0 Hz, 1H), 5.28 (s, 2H), 4.49 (dd, J = 14.9, 10.4 Hz, 1H), 3.98 (dd, J = 14.9, 8.0 Hz, 1H). ^{13}C NMR (100 MHz, $CDCl_3$) δ 161.3 (dd, J = 256.0, 6.0 Hz), 158.0 (d, J = 234.3 Hz), 156.6, 140.3, 137.6, 132.9, 132.5 (t, J = 10.5 Hz), 129.9, 129.1 (d, J = 10.1 Hz), 127.3, 126.3, 112.2 – 111.9 (m), 110.4 (d, J = 3.9 Hz), 110.2 (d, J = 20.6 Hz), 107.3 (t, J = 17.6 Hz), 105.8 (d, J = 23.4 Hz), 101.9 (d, J = 4.7 Hz), 80.8, 63.4,

50.2. HRMS (ESI): m/z calcd for $C_{24}H_{18}F_3N_2O^+$ $[M+H]^+$ 407.1366, found 407.1363.

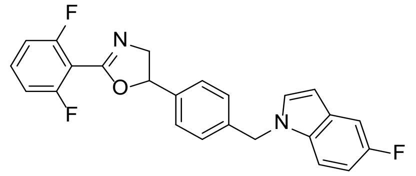

2-(2,6-difluorophenyl)-5-(4-((5-fluoro-1*H*-indol-1-yl)methyl)phenyl)-4,5-dihydrooxazole (**11f**), yellow oil, 85% yield. ^{1}H NMR (400 MHz, $CDCl_3$) δ 7.38 (tt, J = 8.5, 6.2 Hz, 1H), 7.31 (d, J = 8.2 Hz, 2H), 7.29 – 7.23 (m, 1H), 7.17 – 7.06 (m, 4H), 6.96 (t, J = 8.3 Hz, 2H), 6.89 (td, J = 9.1, 2.5 Hz, 1H), 6.50 (d, J = 3.1 Hz, 1H), 5.63 (dd, J = 10.4, 8.0 Hz, 1H), 5.28 (s, 2H), 4.49 (dd, J = 14.9, 10.4 Hz, 1H), 3.98 (dd, J = 14.9, 8.0 Hz, 1H). ^{13}C NMR (100 MHz, $CDCl_3$) δ 161.3 (dd, J = 256.0, 6.0 Hz), 158.0 (d, J = 234.3 Hz), 156.6, 140.3, 137.6, 132.9, 132.5 (t, J = 10.5 Hz), 129.9, 129.1 (d, J = 10.1 Hz), 127.3, 126.3, 112.2 – 111.9 (m), 110.4 (d, J = 3.9 Hz), 110.2 (d, J = 20.6 Hz), 107.3 (t, J = 17.6 Hz), 105.8 (d, J = 23.4 Hz), 101.9 (d, J = 4.7 Hz), 80.8, 63.4, 50.2. HRMS (ESI): m/z calcd for $C_{24}H_{18}F_3N_2O^+$ $[M+H]^+$ 407.1366, found 407.1367.

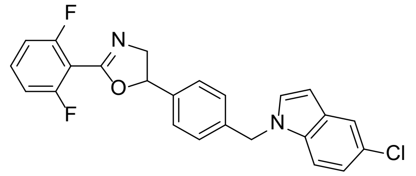

5-(4-((5-chloro-1*H*-indol-1-yl)methyl)phenyl)-2-(2,6-difluorophenyl)-4,5-dihydrooxazole (**11g**), yellow oil, 82% yield. ^{1}H NMR (400 MHz, $CDCl_3$) δ 7.59 (d, J = 1.9 Hz, 1H), 7.38 (tt, J = 8.5, 6.2 Hz, 1H), 7.30 (d, J = 8.3 Hz, 2H), 7.17 – 7.05 (m, 5H), 6.96 (t, J = 8.3 Hz, 2H), 6.48 (d, J = 3.1 Hz, 1H), 5.63 (dd, J = 10.4, 7.9 Hz, 1H), 5.27 (s, 2H), 4.49 (dd, J = 14.9, 10.4 Hz, 1H), 3.97 (dd, J = 14.9, 7.9 Hz, 1H). ^{13}C NMR (100 MHz, $CDCl_3$) δ 161.3 (dd, J = 255.9, 6.0 Hz), 156.6, 140.4, 137.5, 134.7, 132.5 (t, J = 10.4 Hz), 129.8, 129.7, 127.2, 126.3, 125.5, 122.2, 120.5, 112.2 – 111.9 (m), 110.8, 107.3 (t, J = 17.6 Hz), 101.6, 80.8, 63.4, 50.1. HRMS (ESI): m/z calcd for $C_{24}H_{18}ClF_2N_2O^+$ $[M+H]^+$ 423.1070, found 423.1078.

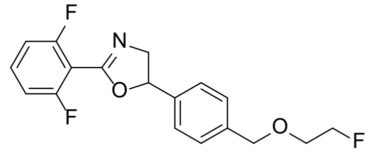

2-(2,6-difluorophenyl)-5-(4-((2-fluoroethoxy)methyl)phenyl)-4,5-dihydrooxazole (**11h**), yellow oil, 16% yield. ^{1}H NMR (400 MHz, $CDCl_3$) δ 7.46 – 7.36 (m, 5H), 6.99 (t, J = 8.2 Hz, 2H), 5.69 (t, J = 10.1 Hz, 1H), 4.68 – 4.48 (m, 5H), 4.03 (dd, J = 14.9, 8.0 Hz, 1H), 3.80 – 3.65 (m, 2H). ^{13}C NMR (100 MHz, $CDCl_3$) δ 161.4 (dd, J = 256.0,

6.1 Hz), 156.7, 140.3, 138.3, 132.4 (t, *J* = 10.4 Hz), 128.3, 126.0, 112.3 – 111.9 (m), 107.4 (t, *J* = 17.8 Hz), 83.2 (d, *J* = 169.3 Hz), 81.0, 73.1, 69.4 (d, *J* = 19.7 Hz), 63.5. HRMS (ESI): m/z calcd for $C_{18}H_{17}F_3NO_2^+$ $[M+H]^+$ 336.1206, found 336.1205.

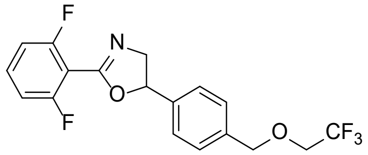

2-(2,6-difluorophenyl)-5-(4-((3,3,3-trifluoropropoxy)methyl)phenyl)-4,5-dihydrooxazole (**11i**), yellow oil, 17% yield. ^1H NMR (400 MHz, $CDCl_3$) δ 7.47 – 7.36 (m, 5H), 7.00 (t, *J* = 8.3 Hz, 2H), 5.71 (dd, *J* = 10.4, 8.0 Hz, 1H), 4.69 (s, 2H), 4.55 (dd, *J* = 14.9, 10.4 Hz, 1H), 4.03 (dd, *J* = 14.9, 8.0 Hz, 1H), 3.83 (q, *J* = 8.7 Hz, 2H). ^{13}C NMR (100 MHz, $CDCl_3$) δ 161.4 (dd, *J* = 256.1, 6.0 Hz), 156.7 (t, *J* = 2.0 Hz), 140.9, 136.9, 132.5 (t, *J* = 10.5 Hz), 128.5, 126.2, 124.2 (q, *J* = 279.3 Hz), 112.3 – 111.9 (m), 107.4 (t, *J* = 17.6 Hz), 80.9, 73.8, 67.4 (q, *J* = 34.1 Hz), 63.5. HRMS (ESI): m/z calcd for $C_{18}H_{15}F_5NO_2^+$ $[M+H]^+$ 372.1017, found 372.1017.

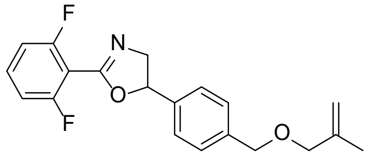

2-(2,6-difluorophenyl)-5-(4-(((2-methylallyl)oxy)methyl)phenyl)-4,5-dihydrooxazole (**11j**), yellow oil, 10% yield. ^1H NMR (400 MHz, $CDCl_3$) δ 7.45–7.37 (m, 5H), 6.98 (t, *J* = 8.3 Hz, 2H), 5.69 (dd, *J* = 10.4, 8.0 Hz, 1H), 5.00 (s, 1H), 4.93 (s, 1H), 4.57 – 4.49 (m, 3H), 4.03 (dd, *J* = 14.9, 8.1 Hz, 1H), 3.93 (s, 2H), 1.77 (s, 3H). ^{13}C NMR (100 MHz, $CDCl_3$) δ 161.4 (dd, *J* = 256.1, 6.1 Hz), 156.7, 142.2, 140.0, 138.9, 132.4 (t, *J* = 10.3 Hz), 128.2, 125.9, 112.5, 112.2 – 111.9 (m), 107.4 (t, *J* = 17.5 Hz), 81.1, 74.3, 71.5, 63.4, 19.7. HRMS (ESI): m/z calcd for $C_{20}H_{20}F_2NO_2^+$ $[M+H]^+$ 344.1457, found, 344.1452.

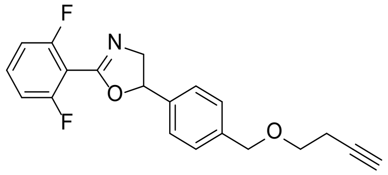

5-(4-((but-3-yn-1-yloxy)methyl)phenyl)-2-(2,6-difluorophenyl)-4,5-dihydrooxazole (**11k**), yellow oil, 15% yield. ^1H NMR (400 MHz, $CDCl_3$) δ 7.45–7.38 (m, 5H), 6.99 (t, *J* = 8.3 Hz, 2H), 5.70 (dd, *J* = 10.4, 8.0 Hz, 1H), 4.57 (s, 2H), 4.53 (dd, *J* = 14.9, 10.4 Hz, 1H), 4.03 (dd, *J* = 14.9, 8.0 Hz, 1H), 3.60 (t, *J* = 6.9 Hz, 2H), 2.51 (td, *J* = 6.9, 2.7 Hz, 2H), 2.00 (t, *J* = 2.7 Hz, 1H). ^{13}C NMR (100 MHz, $CDCl_3$) δ 161.4 (dd, *J* = 256.1,

6.0 Hz), 156.7, 140.2, 138.5, 132.4 (t, *J* = 10.5 Hz), 128.3, 126.0, 112.2 – 111.9 (m), 107.4 (t, *J* = 17.5 Hz), 81.4, 81.1, 72.7, 69.5, 68.3, 63.5, 20.0. HRMS (ESI): m/z calcd for $C_{20}H_{18}F_2NO_2^+$ $[M+H]^+$ 342.1300, found, 342.1306.

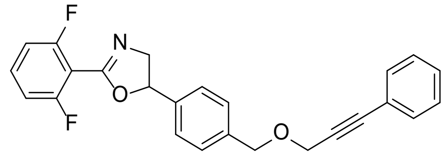

2-(2,6-difluorophenyl)-5-(4-(((3-phenylprop-2-yn-1-yl)oxy)methyl)phenyl)-4,5-dihydrooxazole (**11l**), yellow oil, 37% yield. ^{1}H NMR (400 MHz, $CDCl_3$) δ 7.49 – 7.36 (m, 7H), 7.32–7.30 (m, 3H), 6.98 (t, *J* = 8.3 Hz, 2H), 5.69 (dd, *J* = 10.4, 8.0 Hz, 1H), 4.68 (s, 2H), 4.53 (dd, *J* = 14.9, 10.4 Hz, 1H), 4.40 (s, 2H), 4.02 (dd, *J* = 14.9, 8.0 Hz, 1H). ^{13}C NMR (100 MHz, $CDCl_3$) δ 161.3 (dd, *J* = 255.9, 6.0 Hz), 156.7, 140.4, 138.0, 132.4 (t, *J* = 10.4 Hz), 131.9, 128.7, 128.6, 128.4, 126.0, 122.7, 112.2 – 111.9 (m), 107.4 (t, *J* = 17.7 Hz), 86.7, 85.0, 81.0, 71.3, 63.4, 58.1. HRMS (ESI): m/z calcd for $C_{25}H_{20}F_2NO_2^+$ $[M+H]^+$ 404.1457, found, 404.1452.

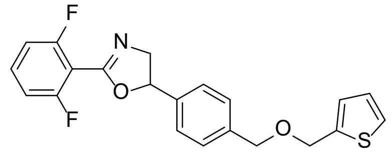

2-(2,6-difluorophenyl)-5-(4-((thiophen-2-ylmethoxy)methyl)phenyl)-4,5-dihydro oxazole (**11m**), yellow oil, 38% yield. ^{1}H NMR (400 MHz, $CDCl_3$) δ 7.45 – 7.36 (m, 5H), 7.30 (dd, *J* = 5.2, 1.3 Hz, 1H), 7.03 – 6.93 (m, 4H), 5.69 (dd, *J* = 10.4, 8.0 Hz, 1H), 4.71 (s, 2H), 4.57 – 4.49 (m, 3H), 4.03 (dd, *J* = 14.9, 8.0 Hz, 1H). ^{13}C NMR (100 MHz, $CDCl_3$) δ 161.3 (dd, *J* = 256.2, 6.2 Hz), 156.7, 141.0, 140.2, 138.4, 132.4 (t, *J* = 10.5 Hz), 128.4, 126.8, 126.7, 126.02, 125.95, 112.2 – 111.9 (m), 107.4 (t, *J* = 17.7 Hz), 81.1, 71.3, 66.6, 63.4. HRMS (ESI): m/z calcd for $C_{21}H_{18}F_2NO_2S^+$ $[M+H]^+$ 386.1021, found, 386.1023.

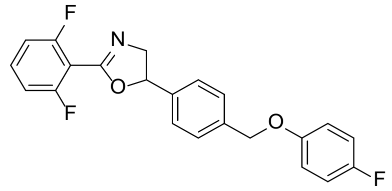

2-(2,6-difluorophenyl)-5-(4-((4-fluorophenoxy)methyl)phenyl)-4,5-dihydrooxazole (**11n**), colorless oil, 42% yield. ^{1}H NMR (400 MHz, $CDCl_3$) δ 7.46–7.37 (m, 5H), 7.02 – 6.93 (m, 4H), 6.92 – 6.86 (m, 2H), 5.70 (dd, *J* = 10.4, 8.0 Hz, 1H), 5.02 (s, 2H), 4.54 (dd, *J* = 14.9, 10.4 Hz, 1H), 4.03 (dd, *J* = 14.9, 8.0 Hz, 1H). ^{1}H NMR (400 MHz, $CDCl_3$) δ 161.4 (dd, *J* = 256.1, 6.0 Hz), 157.5 (d, *J* = 238.4 Hz), 156.7, 154.9 (d, *J* = 1.9 Hz),

140.6, 137.2, 132.5 (t, *J* = 10.4 Hz), 128.0, 126.1, 115.97 (d, *J* = 7.9 Hz), 115.96 (d, *J* = 23.1 Hz), 112.3 – 111.8 (m), 107.3 (t, *J* = 17.7 Hz), 80.9, 70.3, 63.5. HRMS (ESI): m/z calcd for $C_{22}H_{17}F_3NO_2^+$ $[M+H]^+$ 384.1206, found, 384.1204.

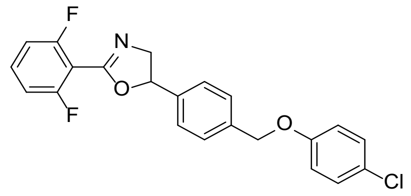

5-(4-((4-chlorophenoxy)methyl)phenyl)-2-(2,6-difluorophenyl)-4,5-dihydrooxazole (**11o**), white solid, m.p. 85 – 87 °C, 56% yield. ^{1}H NMR (400 MHz, $CDCl_3$) δ 7.46 – 7.38 (m, 4H), 7.22 (d, *J* = 9.0 Hz, 2H), 6.98 (t, *J* = 8.1 Hz, 2H), 6.88 (d, *J* = 9.0 Hz, 2H), 5.70 (dd, *J* = 10.4, 8.0 Hz, 1H), 5.03 (s, 2H), 4.54 (dd, *J* = 14.9, 10.4 Hz, 1H), 4.03 (dd, *J* = 15.0, 7.9 Hz, 1H). ^{13}C NMR (100 MHz, $CDCl_3$) δ 161.4 (dd, *J* = 256.1, 6.0 Hz), 157.3, 156.7, 140.7, 137.0, 132.5 (t, *J* = 10.4 Hz), 129.5, 128.0, 126.1, 126.0, 116.3, 112.2 – 111.9 (m), 107.4 (t, *J* = 17.7 Hz), 80.9, 70.0, 63.4. HRMS (ESI): m/z calcd for $C_{22}H_{17}ClF_2NO_2^+$ $[M+H]^+$ 400.0910, found, 400.0915.

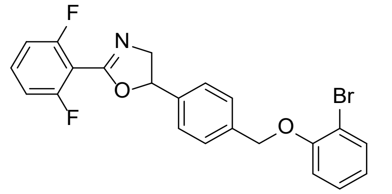

5-(4-((2-bromophenoxy)methyl)phenyl)-2-(2,6-difluorophenyl)-4,5-dihydrooxazole (**11p**), yellow oil, 86% yield. ^{1}H NMR (400 MHz, $CDCl_3$) δ 7.55 (dd, *J* = 7.9, 1.6 Hz, 1H), 7.51 (d, *J* = 7.9 Hz, 2H), 7.45 – 7.36 (m, 3H), 7.26 – 7.20 (m, 1H), 6.98 (t, *J* = 8.4 Hz, 2H), 6.91 (d, *J* = 8.2 Hz, 1H), 6.84 (td, *J* = 7.6, 1.3 Hz, 1H), 5.70 (dd, *J* = 10.4, 8.0 Hz, 1H), 5.15 (s, 2H), 4.53 (dd, *J* = 14.9, 10.4 Hz, 1H), 4.03 (dd, *J* = 14.9, 8.1 Hz, 1H). ^{13}C NMR (100 MHz, $CDCl_3$) δ 161.4 (dd, *J* = 256.0, 6.0 Hz), 156.7, 155.0, 140.4, 136.9, 133.6, 132.4 (t, *J* = 10.4 Hz), 128.5, 127.6, 126.1, 122.4, 114.0, 112.6, 112.2 – 111.9 (m), 107.4 (t, *J* = 17.7 Hz), 81.0, 70.5, 63.5. HRMS (ESI): m/z calcd for $C_{22}H_{17}BrF_2NO_2^+$ $[M+H]^+$ 444.0405, found, 444.0403.

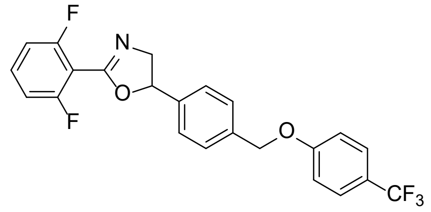

2-(2,6-difluorophenyl)-5-(4-((4-(trifluoromethyl)phenoxy)methyl)phenyl)-4,5-dihydrooxazole (**11q**), white solid, m.p. 105 – 107 °C, 85% yield. ^{1}H NMR (400 MHz,

CDCl$_3$) δ 7.57 (d, J = 8.6 Hz, 2H), 7.52 – 7.40 (m, 5H), 7.08 – 6.98 (m, 4H), 5.74 (dd, J = 10.4, 7.9 Hz, 1H), 5.14 (s, 2H), 4.58 (dd, J = 14.9, 10.4 Hz, 1H), 4.07 (dd, J = 14.9, 7.9 Hz, 1H). **^{13}C** NMR (100 MHz, CDCl$_3$) δ 161.3 (dd, J = 256.0, 6.0 Hz), 161.1, 156.6, 140.8, 136.5, 132.4 (t, J = 10.5 Hz), 127.9, 127.0 (q, J = 3.9 Hz), 126.1, 124.9 (q, J = 271.5 Hz), 123.2 (q, J = 32.4 Hz), 114.8, 112.2 – 111.8 (m), 107.2 (t, J = 17.7 Hz), 80.8, 69.7, 63.4. HRMS (ESI): m/z calcd for $C_{23}H_{17}F_5NO_2^+$ $[M+H]^+$ 434.1174, found, 434.1173.

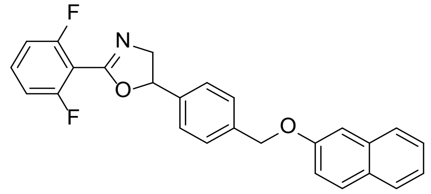

2-(2,6-difluorophenyl)-5-(4-((naphthalen-2-yloxy)methyl)phenyl)-4,5-dihydrooxazole (**11r**), white solid, m.p. 127 – 129 °C, 37% yield. ^{1}H NMR (400 MHz, CDCl$_3$) δ 7.80 – 7.68 (m, 3H), 7.52 (d, J = 8.1 Hz, 2H), 7.45 – 7.31 (m, 5H), 7.27 – 7.19 (m, 2H), 6.98 (t, J = 8.3 Hz, 2H), 5.71 (dd, J = 10.4, 8.0 Hz, 1H), 5.18 (s, 2H), 4.54 (dd, J = 14.9, 10.4 Hz, 1H), 4.05 (dd, J = 14.9, 8.0 Hz, 1H). ^{13}C NMR (100 MHz, CDCl$_3$) δ 161.4 (dd, J = 256.0, 6.0 Hz), 156.72, 156.69, 140.5, 137.3, 134.6, 132.4 (t, J = 10.4 Hz), 129.6, 129.2, 128.1, 127.8, 126.9, 126.5, 126.1, 123.9, 119.1, 112.3 – 111.9 (m), 107.4 (t, J = 17.7 Hz), 107.3, 81.0, 69.7, 63.4. HRMS (ESI): m/z calcd for $C_{26}H_{20}F_2NO_2^+$ $[M+H]^+$ 416.1457, found, 416.1455.

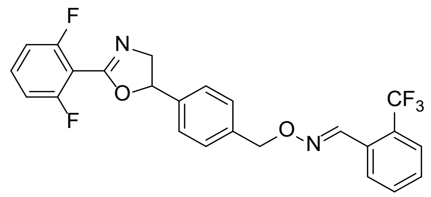

(E)-2-(trifluoromethyl)benzaldehyde O-(4-(2-(2,6-difluorophenyl)-4,5-dihydrooxazol-5-yl)benzyl)oxime (**11s**), yellow oil, 65% yield. ^{1}H NMR (400 MHz, CDCl$_3$) δ 8.52 (dd, J = 4,6, 2.3 Hz, 1H), 8.05 (d, J = 7.8 Hz, 1H), 7.66 (d, J = 7.2 Hz, 1H), 7.53 (t, J = 7.6 Hz, 1H), 7.49 – 7.44 (m, 3H), 7.44 – 7.37 (m, 3H), 6.98 (t, J = 8.3 Hz, 2H), 5.71 (dd, J = 10.4, 8.0 Hz, 1H), 5.26 (s, 2H), 4.54 (dd, J = 14.9, 10.4 Hz, 1H), 4.04 (dd, J = 14.9, 8.0 Hz, 1H). ^{13}C NMR (100 MHz, CDCl$_3$) δ 161.4 (dd, J = 256.0, 6.1 Hz), 156.7, 145.9 (q, J = 1.9 Hz), 140.6, 137.6, 132.4 (t, J = 10.4 Hz), 132.0 (q, J = 1.4 Hz), 130.4 (q, J = 1.7 Hz), 129.6, 129.0, 128.3 (q, J = 31.1 Hz), 127.4, 126.0, 125.9 (q, J = 5.6 Hz), 124.0 (q, J = 273.9 Hz), 112.3 – 111.8 (m), 107.4 (t, J = 17.7 Hz), 81.0, 76.4, 63.5. HRMS (ESI): m/z calcd for $C_{24}H_{18}F_5N_2O_2^+$ $[M+H]^+$ 461.1283, found, 461.1285.

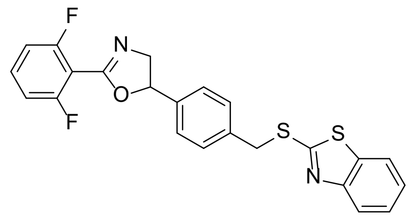

5-(4-((benzo[*d*]thiazol-2-ylthio)methyl)phenyl)-2-(2,6-difluorophenyl)-4,5-dihydrooxazole (**11t**), white solid, m.p. 95 – 97 °C, 62% yield. ^{1}H NMR (400 MHz, $CDCl_3$) δ 7.89 (d, J = 8.5 Hz, 1H), 7.74 (d, J = 8.0 Hz, 1H), 7.49 (d, J = 8.2 Hz, 2H), 7.44 – 7.37 (m, 2H), 7.35 (d, J = 8.2 Hz, 2H), 7.31 – 7.26 (m, 1H), 6.97 (t, J = 8.3 Hz, 2H), 5.66 (dd, J = 10.4, 8.0 Hz, 1H), 4.60 (s, 2H), 4.51 (dd, J = 14.9, 10.4 Hz, 1H), 4.01 (dd, J = 14.9, 8.0 Hz, 1H). ^{13}C NMR (100 MHz, $CDCl_3$) δ 166.2, 161.3 (dd, J = 256.1, 6.0 Hz), 156.7 (t, J = 2.3 Hz), 153.2, 140.2, 136.7, 135.4, 132.4 (t, J = 10.5 Hz), 129.7, 126.2, 124.4, 121.7, 121.1, 112.2 – 111.9 (m), 107.3 (t, J = 17.6 Hz), 80.9, 63.4, 37.3. HRMS(ESI): m/z calcd for $C_{23}H_{17}F_2N_2OS_2^+$ $[M+H]^+$ 439.0745, found, 439.0747.

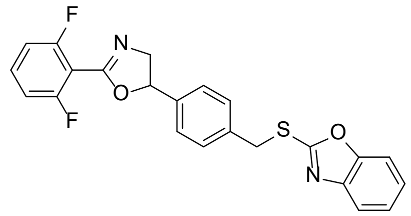

2-((4-(2-(2,6-difluorophenyl)-4,5-dihydrooxazol-5-yl)benzyl)thio)benzo[*d*]oxazole (**11u**), yellow oil, 18% yield. ^{1}H NMR (400 MHz, $CDCl_3$) δ7.62 (d, J = 8.2 Hz, 1H), 7.50 (d, J = 7.9 Hz, 2H), 7.43 (d, J = 8.7 Hz, 1H), 7.36 (d, J = 8.0 Hz, 2H), 7.31 – 7.21 (m, 3H), 6.97 (t, J = 8.5 Hz, 2H), 5.67 (dd, J = 10.4, 8.0 Hz, 1H), 4.56 (s, 2H), 4.51 (dd, J = 15.0, 10.5 Hz, 1H), 4.00 (dd, J = 14.9, 8.0 Hz, 1H). ^{13}C NMR (100 MHz, $CDCl_3$) δ 164.4, 161.3 (dd, J = 256.1, 6.0 Hz), 156.7, 152.0, 142.0, 140.4, 136.4, 132.5 (t, J = 10.4 Hz), 129.7, 126.2, 124.4, 124.1, 118.6, 112.6 – 111.6 (m), 110.0, 107.2 (t, J = 17.7 Hz), 80.8, 63.3, 36.2. HRMS(ESI): m/z calcd for $C_{23}H_{17}F_2N_2O_2S^+$ $[M+H]^+$ 423.0973, found, 423.0976.

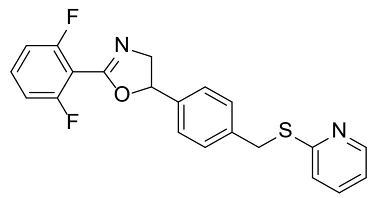

2-(2,6-difluorophenyl)-5-(4-((pyridin-2-ylthio)methyl)phenyl)-4,5-dihydrooxazole (**11v**), yellow oil, 53% yield. ^{1}H NMR (400 MHz, $CDCl_3$) δ 8.45 (d, J = 4.5 Hz, 1H), 7.48 – 7.35 (m, 4H), 7.31 (d, J = 8.1 Hz, 2H), 7.14 (d, J = 8.0 Hz, 1H), 7.02 – 6.90 (m, 3H), 5.65 (dd, J = 10.4, 8.0 Hz, 1H), 4.50 (dd, J = 14.9, 10.4 Hz, 1H), 4.44 (s, 2H), 4.01

(dd, J = 14.9, 8.1 Hz, 1H). ^{13}C NMR (100 MHz, $CDCl_3$) δ 161.3 (dd, J = 256.1, 6.0 Hz), 158.5, 156.6 (t, J = 2.1 Hz), 149.5, 139.4, 138.6, 136.1, 132.4 (t, J = 10.4 Hz), 129.5, 126.0, 122.2, 119.7, 112.4 – 111.7 (m), 107.4 (t, J = 17.7 Hz), 81.0, 63.3, 34.0. HRMS(ESI): m/z calcd for $C_{21}H_{17}F_2N_2OS^+$ $[M+H]^+$ 383.1024, found, 383.1028.

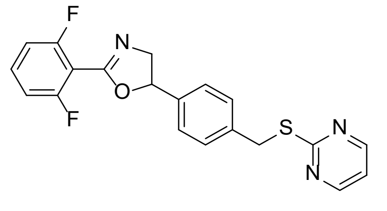

2-(2,6-difluorophenyl)-5-(4-((pyrimidin-2-ylthio)methyl)phenyl)-4,5-dihydro oxazole (**11w**), yellow oil, 80% yield. ^{1}H NMR (400 MHz, $CDCl_3$) δ 8.51 (d, J = 4.9 Hz, 2H), 7.47 (d, J = 8.2 Hz, 2H), 7.40 (tt, J = 8.5, 6.2 Hz, 1H), 7.33 (d, J = 8.2 Hz, 2H), 7.02 – 6.92 (m, 3H), 5.66 (dd, J = 10.4, 8.1 Hz, 1H), 4.51 (dd, J = 14.9, 10.4 Hz, 1H), 4.41 (s, 2H), 4.01 (dd, J = 14.9, 8.0 Hz, 1H). ^{13}C NMR (100 MHz, $CDCl_3$) δ 172.0, 161.3 (dd, J = 256.0, 6.1 Hz), 157.3, 156.6 (t, J = 2.0 Hz), 139.6, 138.0, 132.4 (t, J = 10.4 Hz), 129.6, 126.0, 116.7, 112.3 – 111.8 (m), 107.4 (t, J = 17.6 Hz), 81.0, 63.3, 34.9. HRMS(ESI): m/z calcd for $C_{20}H_{16}F_2N_3OS^+$ $[M+H]^+$ 384.0977, found, 384.0979.

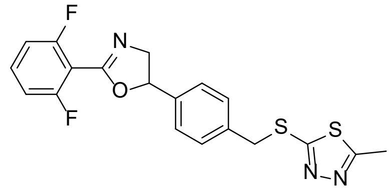

2-(2,6-difluorophenyl)-5-(4-(((5-methyl-1,3,4-thiadiazol-2-yl)thio)methyl)phenyl) - 4,5-dihydrooxazole (**11x**), yellow oil, 35% yield. ^{1}H NMR (400 MHz, $CDCl_3$) δ 7.56 – 7.37 (m, 3H), 7.34 (d, J = 7.9 Hz, 2H), 6.98 (t, J = 8.4 Hz, 2H), 5.67 (dd, J = 10.4, 8.0 Hz, 1H), 4.58 – 4.45 (m, 3H), 4.00 (dd, J = 14.9, 8.0 Hz, 1H), 2.70 (s, 3H). ^{13}C NMR (100 MHz, $CDCl_3$) δ 165.3, 164.7, 161.3 (dd, J = 256.2, 6.1 Hz), 156.6, 140.4, 136.5, 132.5 (t, J = 10.4 Hz), 129.8, 126.2, 112.1 (d, J = 22.7 Hz), 107.3 (t, J = 17.1 Hz), 80.8, 63.4, 37.8, 15.7. HRMS(ESI): m/z calcd for $C_{19}H_{16}F_2N_3OS_2^+$ $[M+H]^+$ 404.0697, found, 404.0697.

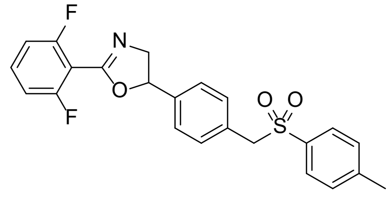

2-(2,6-difluorophenyl)-5-(4-(tosylmethyl)phenyl)-4,5-dihydrooxazole (**11y**), yellow oil, 64% yield. ^{1}H NMR (400 MHz, $CDCl_3$) δ 7.50 (d, J = 8.3 Hz, 2H), 7.42 (tt, J = 8.5,

6.2 Hz, 1H), 7.28 (d, *J* = 8.1 Hz, 2H), 7.24 (d, *J* = 8.0 Hz, 2H), 7.12 (d, *J* = 8.2 Hz, 2H), 6.98 (t, *J* = 8.3 Hz, 2H), 5.67 (dd, *J* = 10.4, 7.9 Hz, 1H), 4.52 (dd, *J* = 14.9, 10.5 Hz, 1H), 4.29 (s, 2H), 3.96 (dd, *J* = 14.9, 7.9 Hz, 1H), 2.41 (s, 3H). ^{13}C NMR (100 MHz, $CDCl_3$) δ 161.3 (dd, *J* = 256.0, 6.2 Hz), 156.7, 144.9, 141.4, 135.0, 132.6 (t, *J* = 10.4 Hz), 131.4, 129.7 (d, *J* = 4.0 Hz), 128.7, 128.5, 125.9, 112.1 (d, *J* = 23.8 Hz), 107.2 (t, *J* = 17.5 Hz), 80.6, 63.4, 62.6, 21.7. HRMS(ESI): m/z calcd for $C_{23}H_{20}F_2NO_3S^+$ $[M+H]^+$ 428.1126, found, 428.1132.

4. ^{1}H and ^{13}C NMR spectrums of intermediates 4-10 and target compounds 11a–11y.

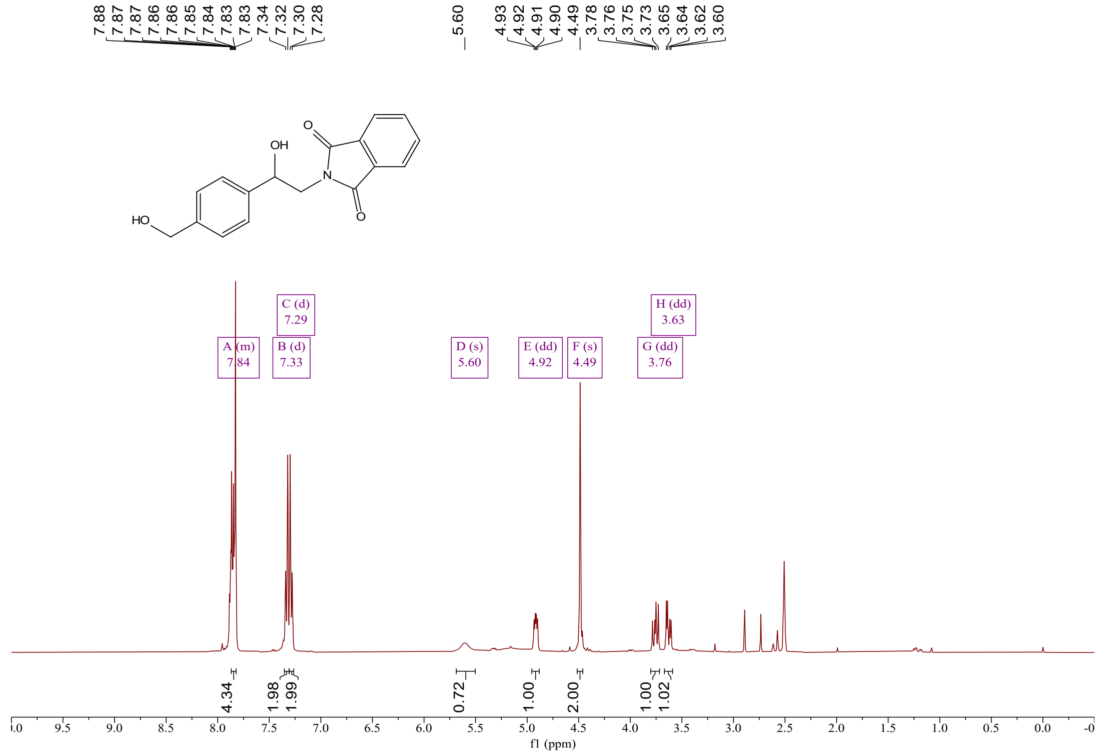

^{1}H NMR spectra of intermediate **4**

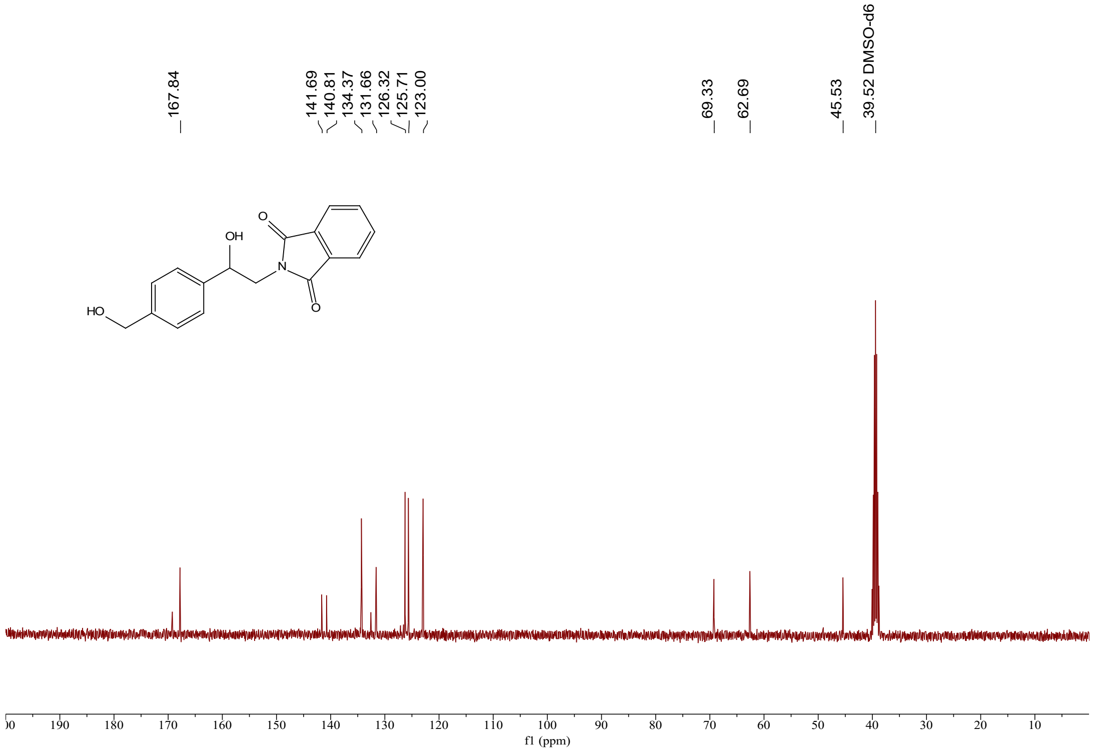

^{13}C NMR spectra of intermediate **4**

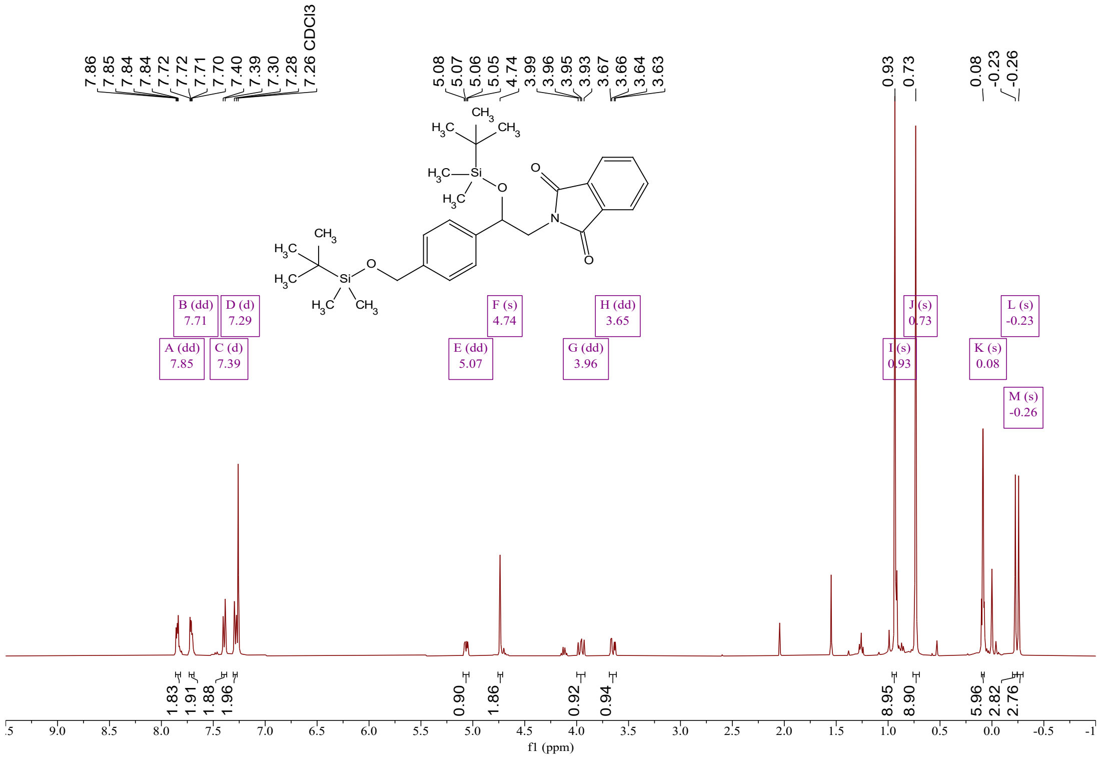

^{1}H NMR spectra of intermediate **5**

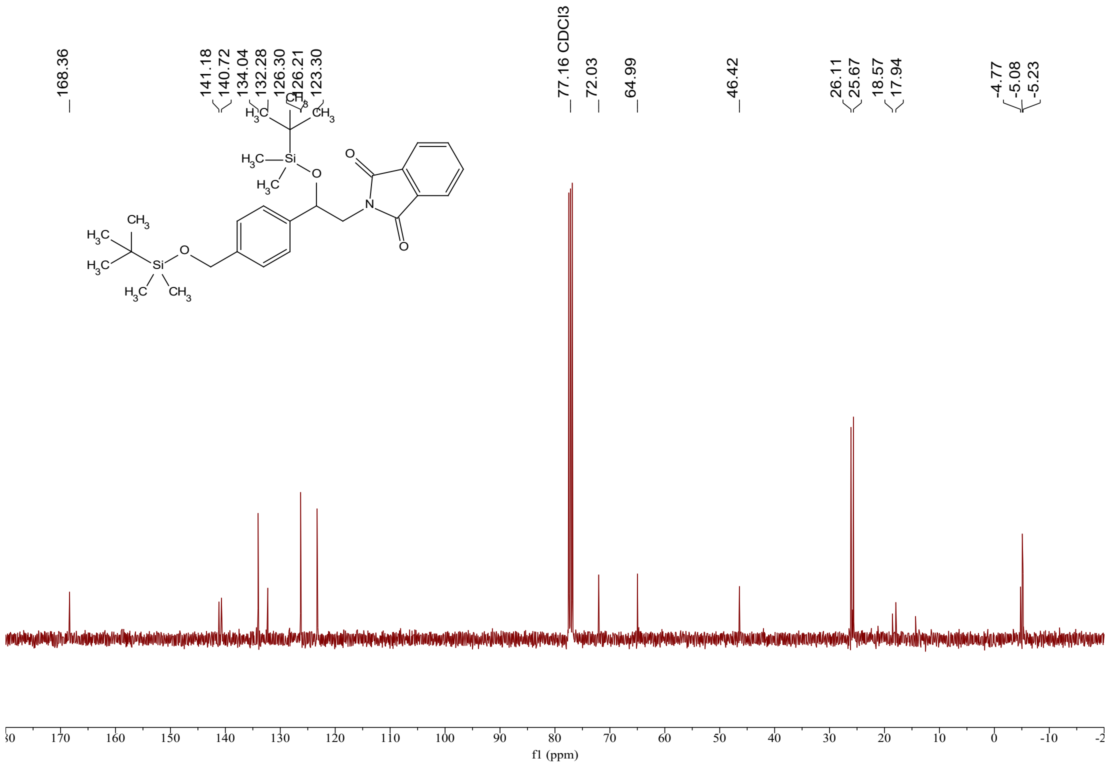

^{13}C NMR spectra of intermediate **5**

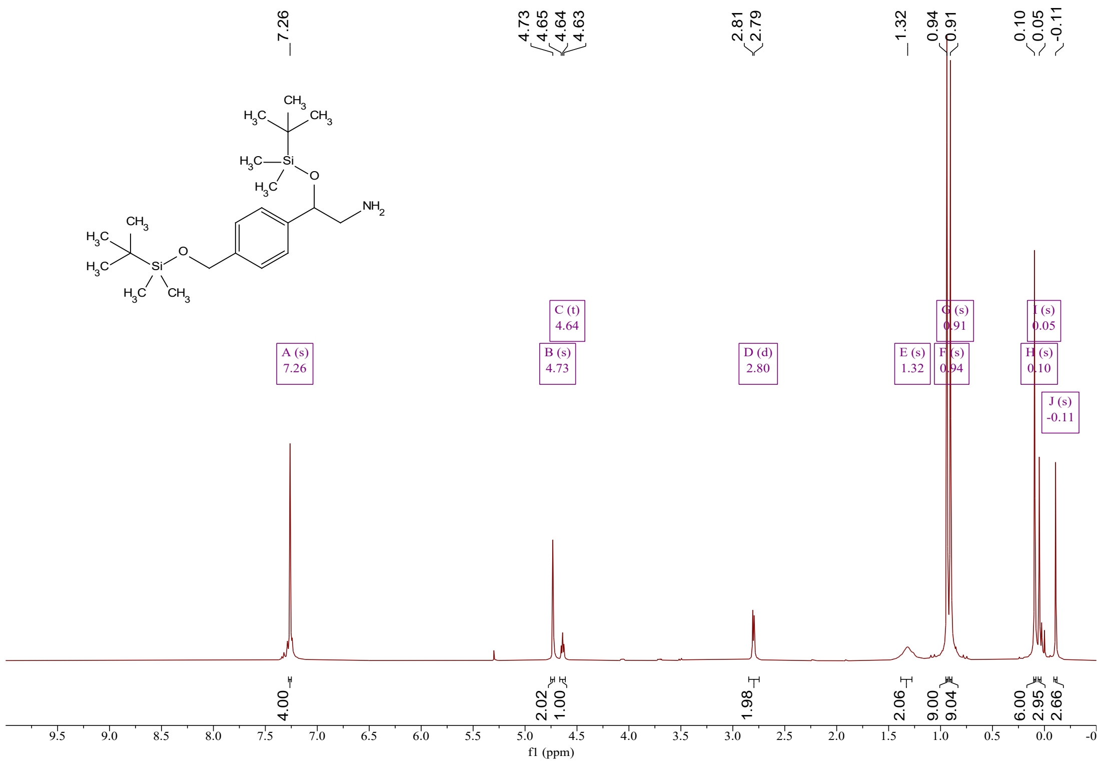

^{1}H NMR spectra of intermediate **6**

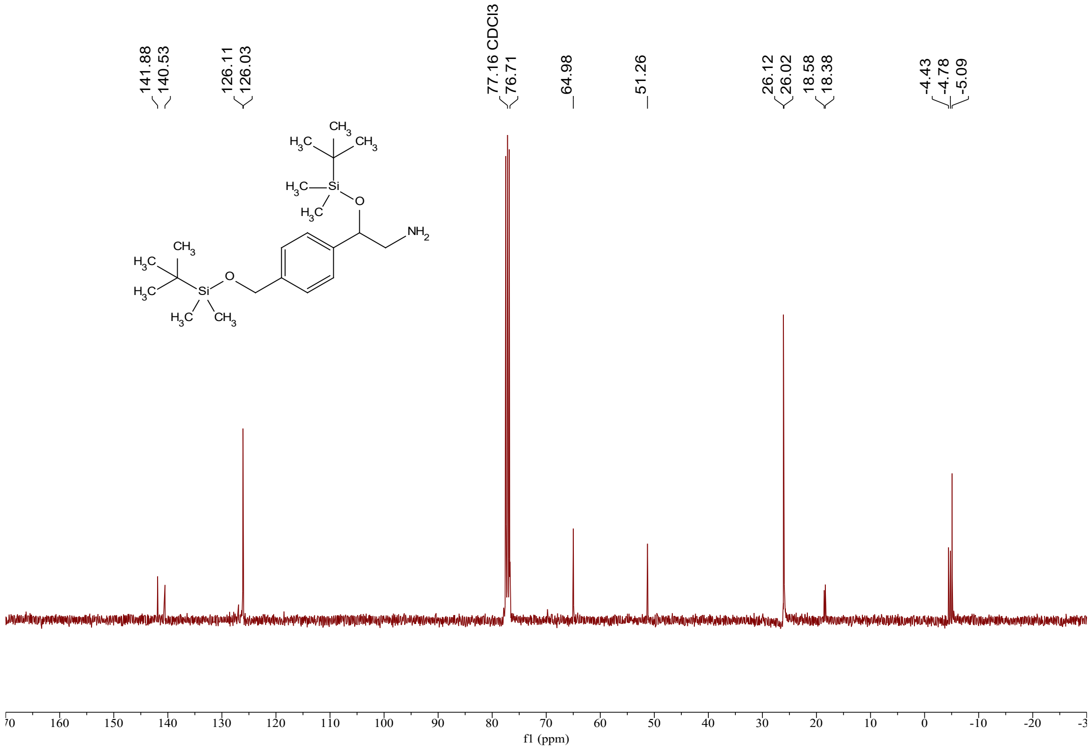

^{13}C NMR spectra of intermediate **6**

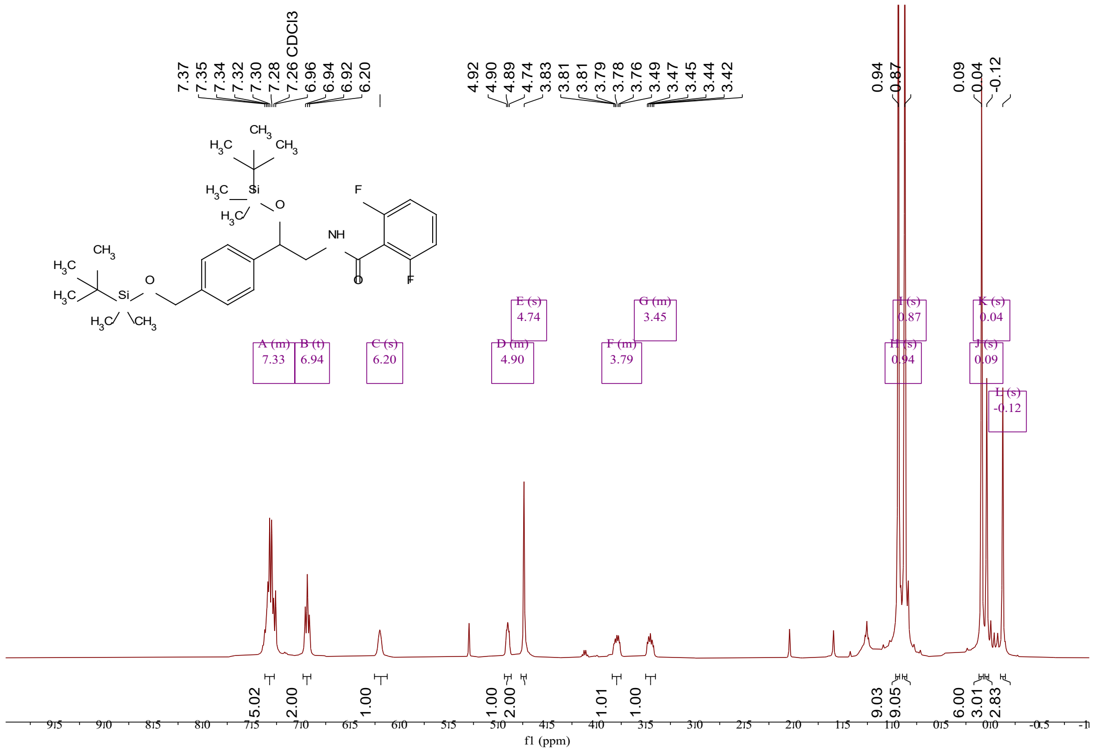

^{1}H NMR spectra of intermediate **7**

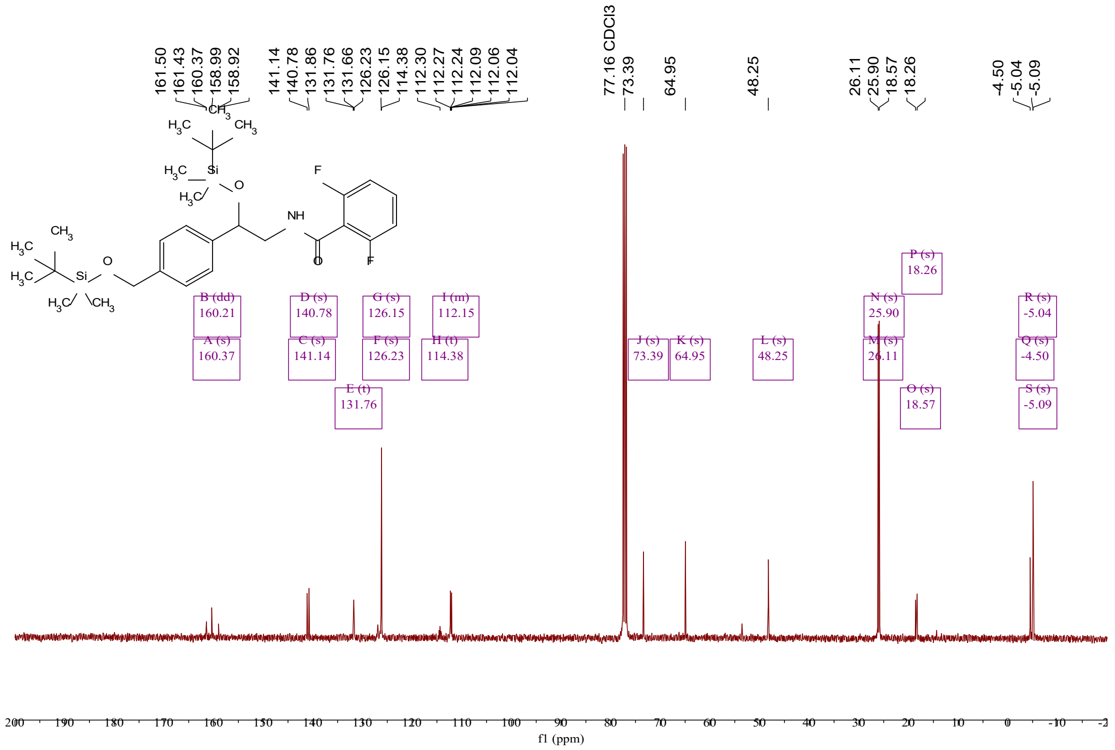

^{13}C NMR spectra of intermediate **7**

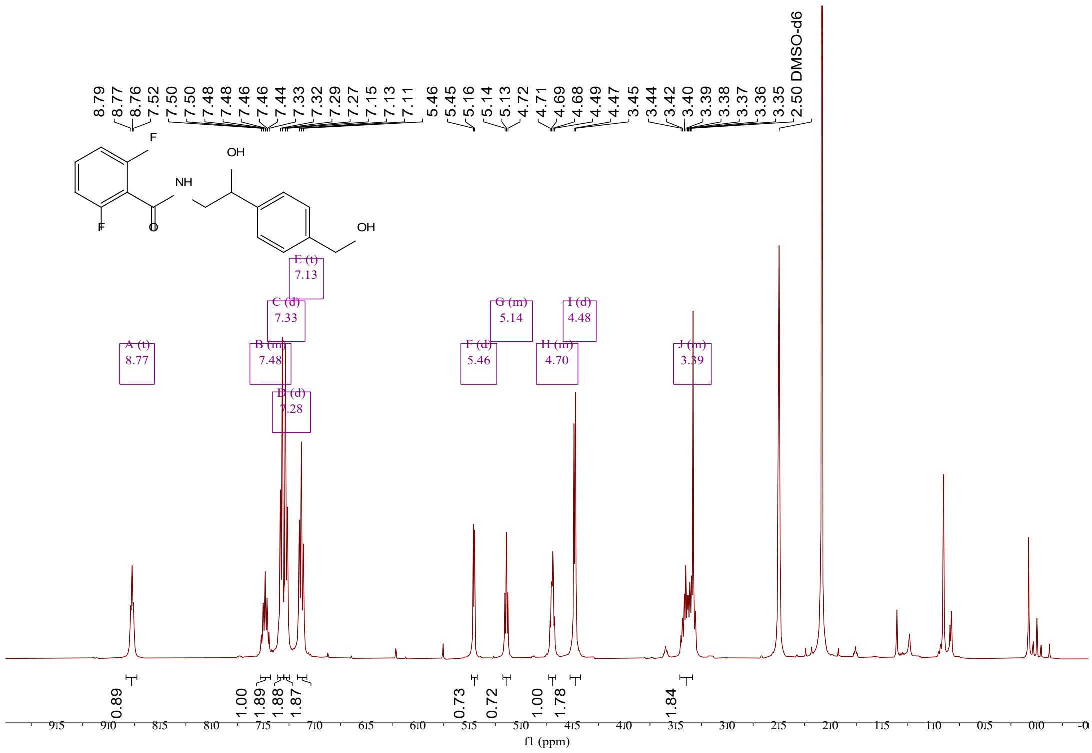

^{1}H NMR spectra of intermediate **8**

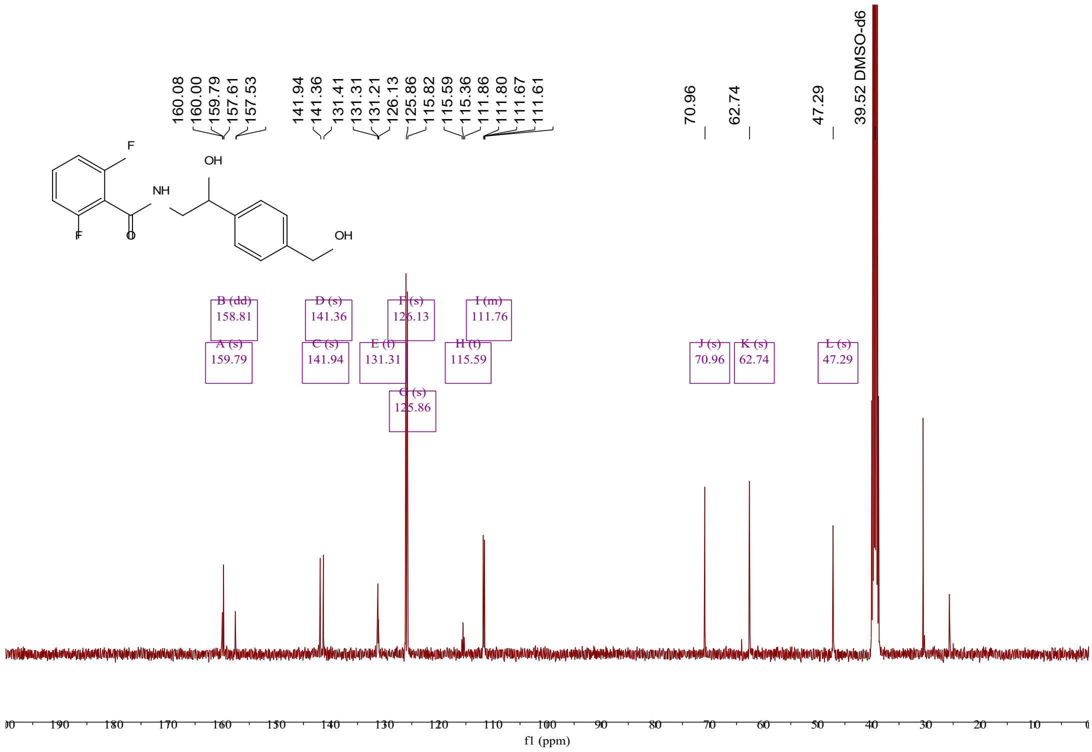

^{13}C NMR spectra of intermediate **8**

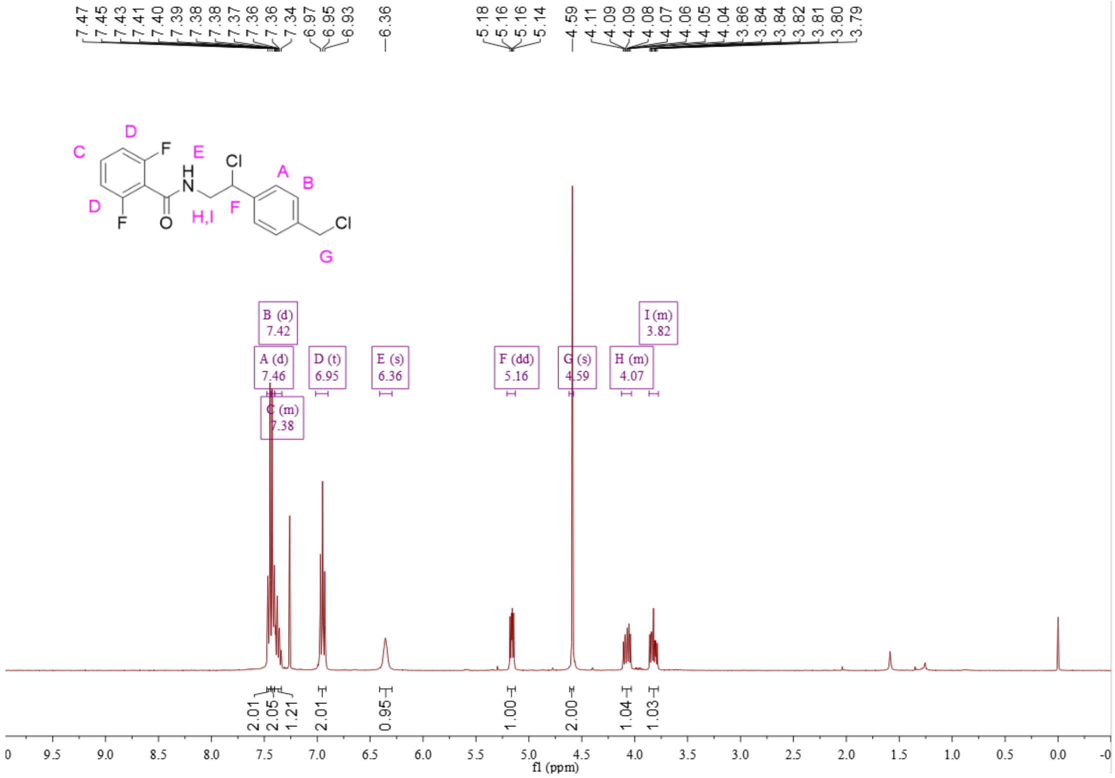

^{1}H NMR spectra of intermediate **9**

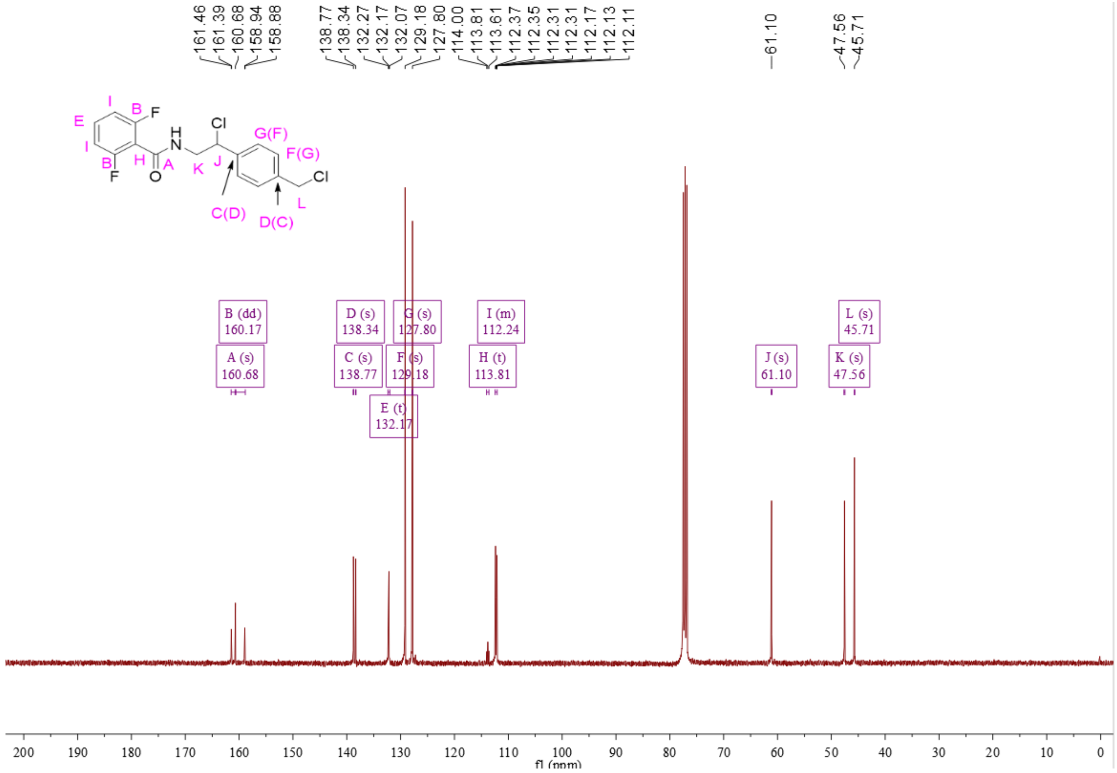

^{13}C NMR spectra of intermediate **9**

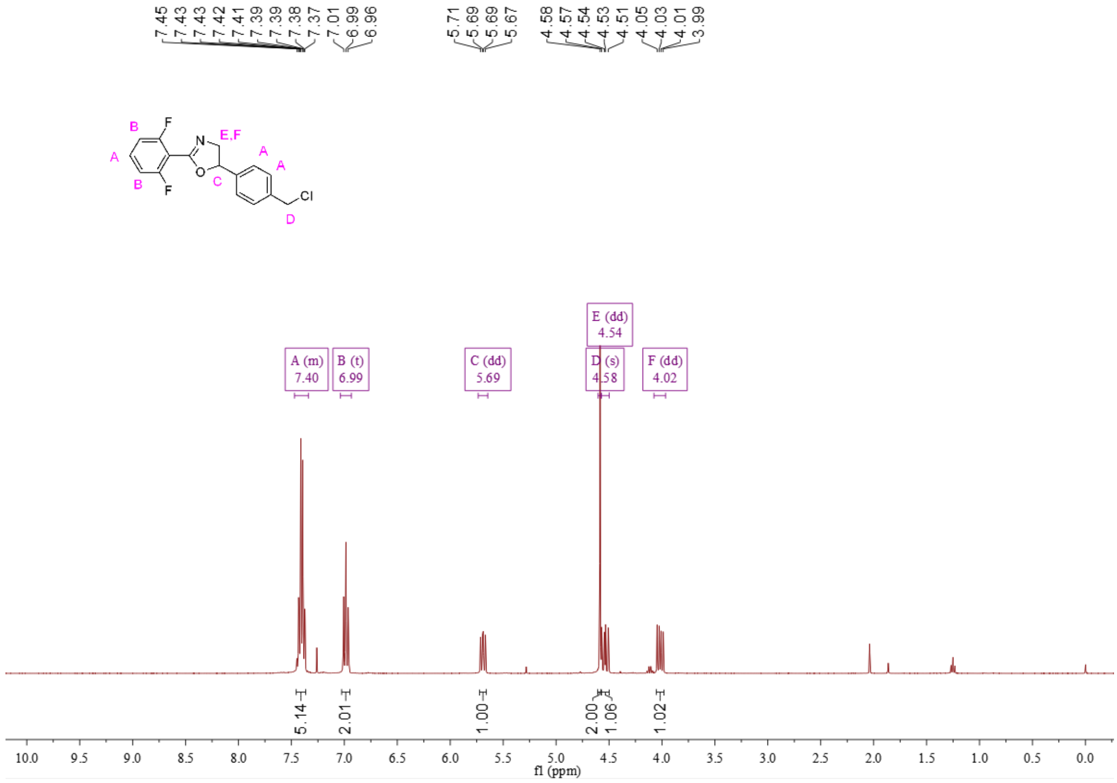

^{1}H NMR spectra of intermediate **10**

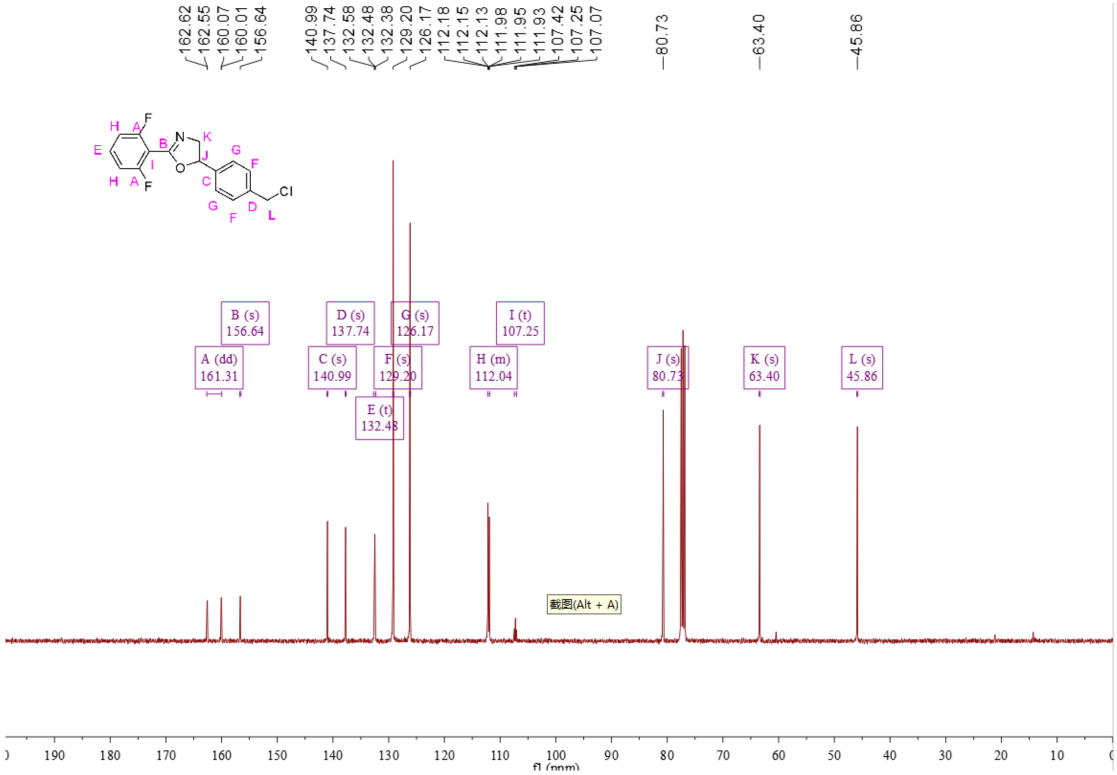

^{13}C NMR spectra of intermediate **10**

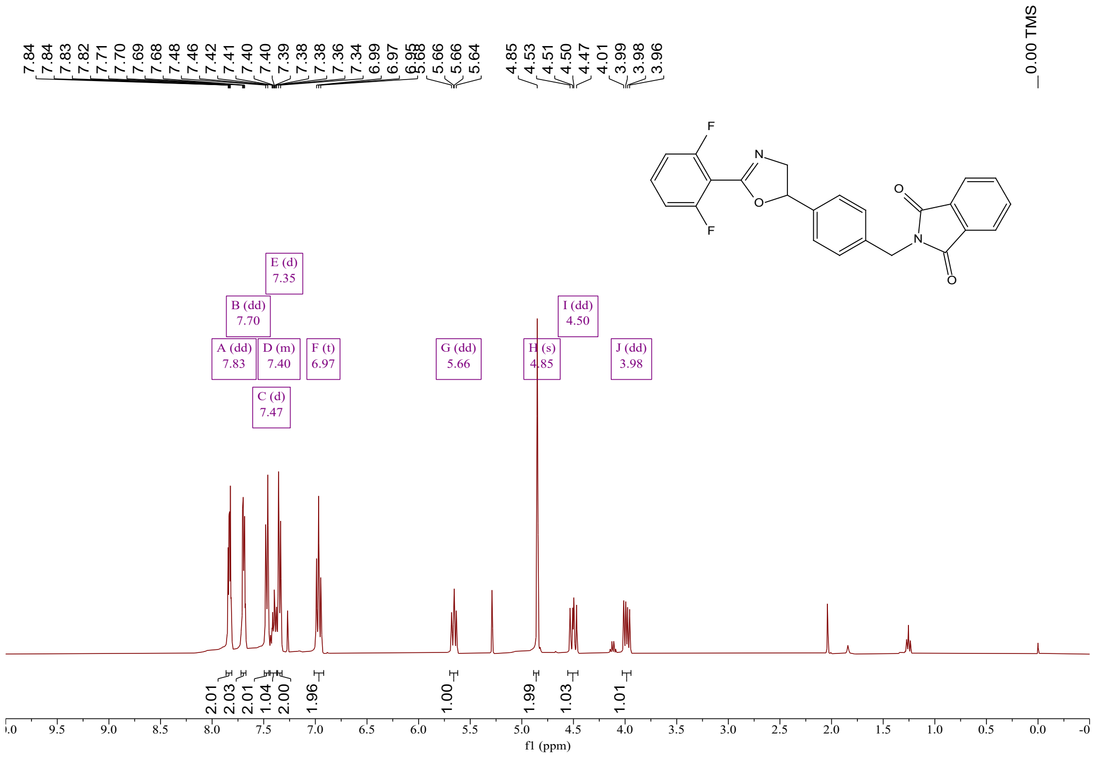

^{1}H NMR spectra of target compound **11a**

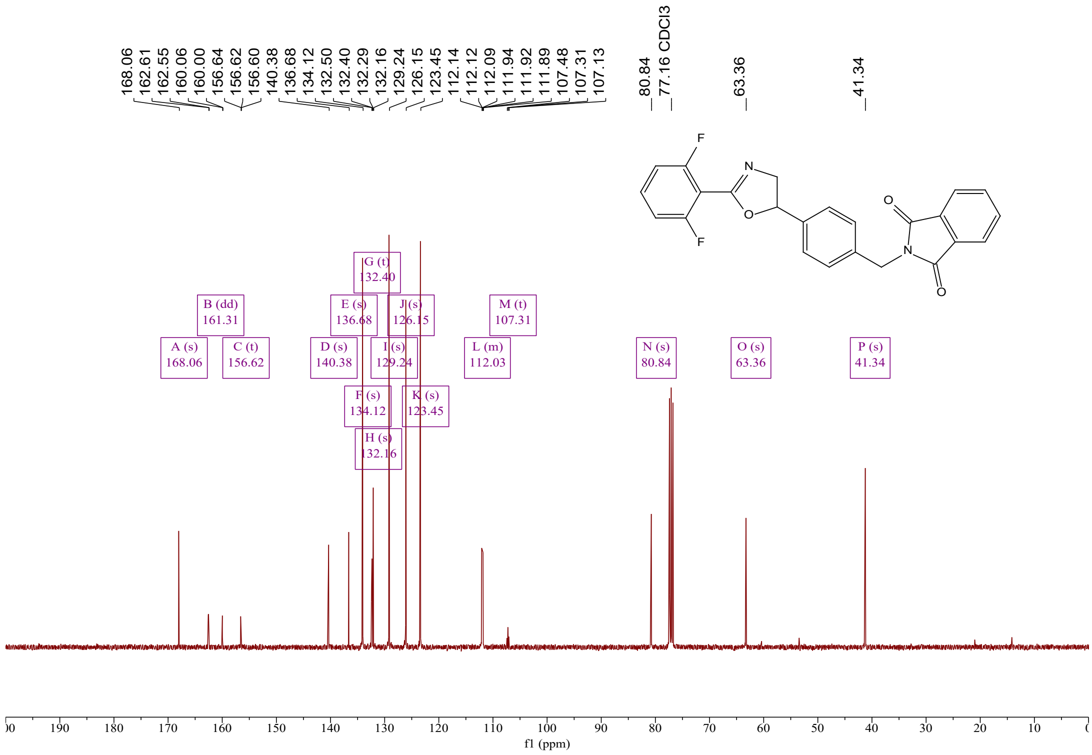

^{13}C NMR spectra of target compound **11a**

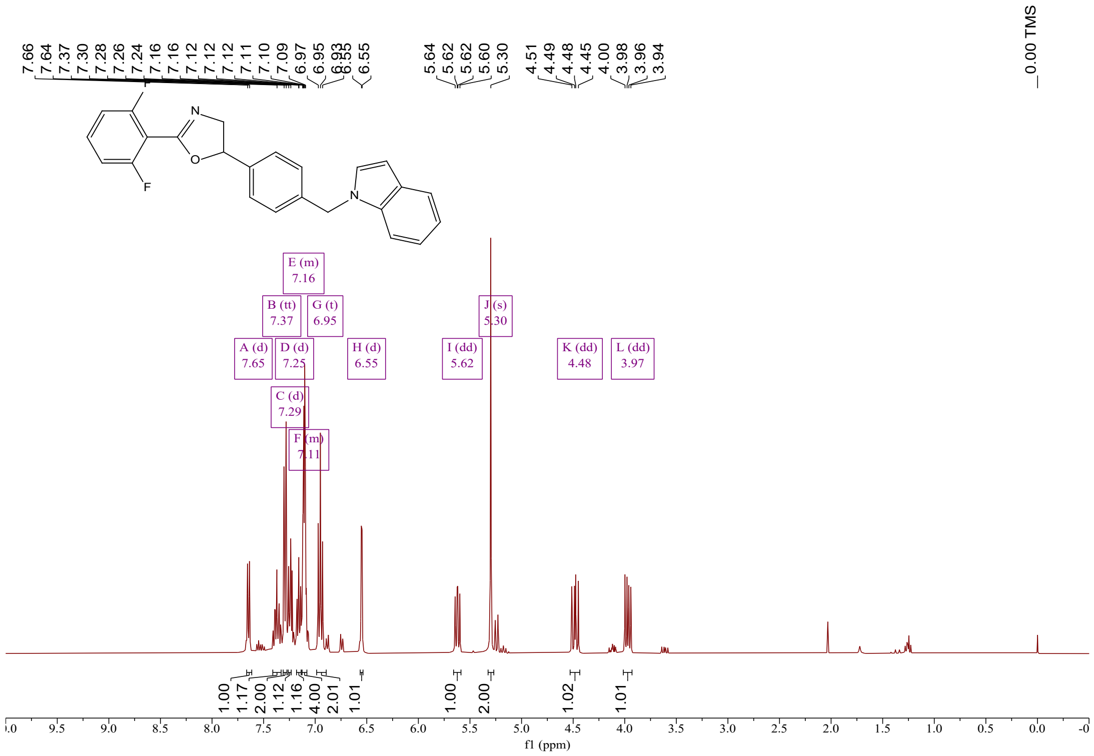

^{1}H NMR spectra of target compound **11b**

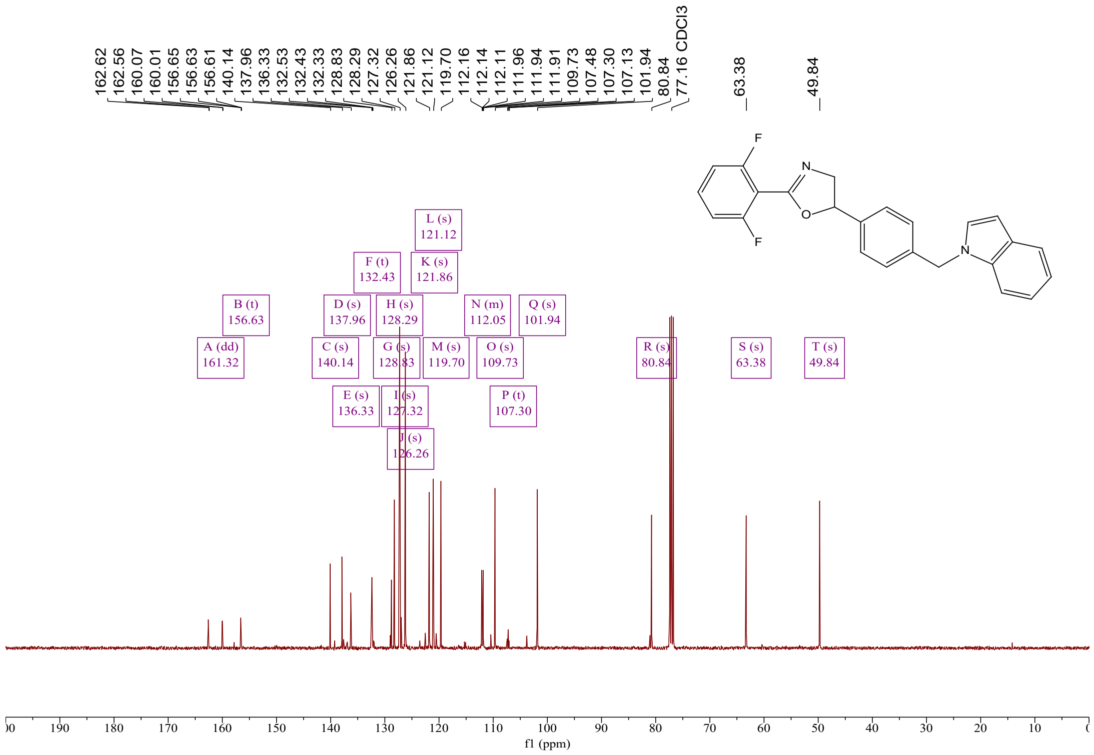

^{13}C NMR spectra of target compound **11b**

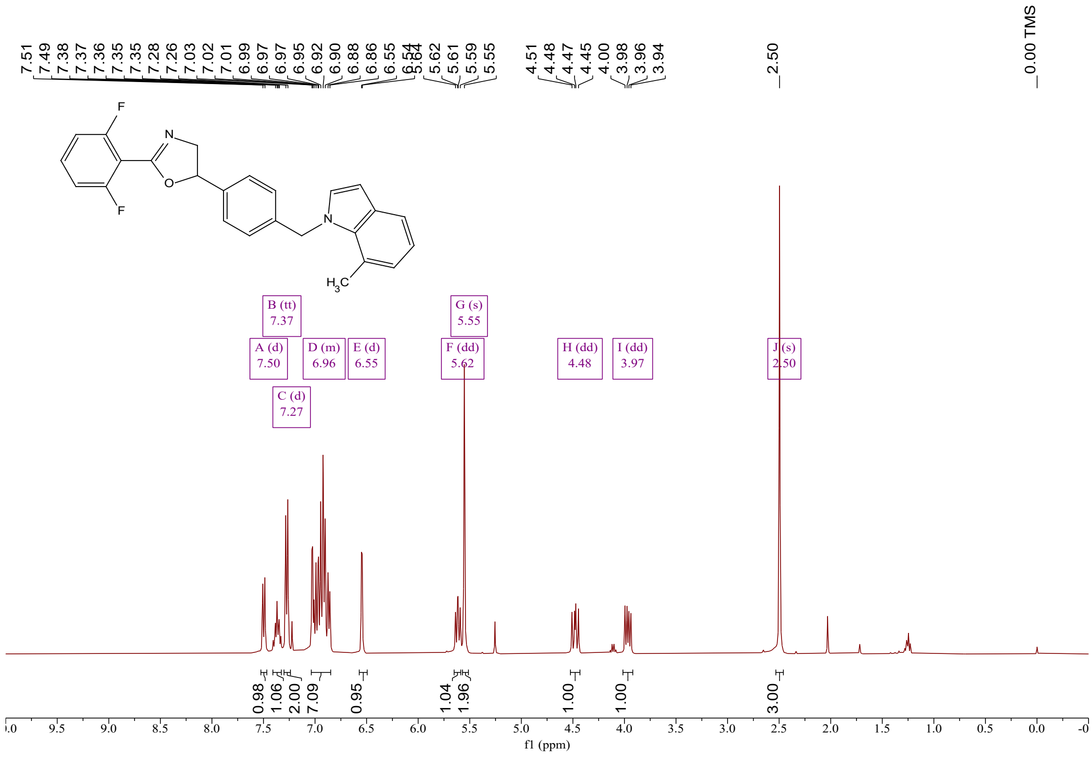

^{1}H NMR spectra of target compound **11c**

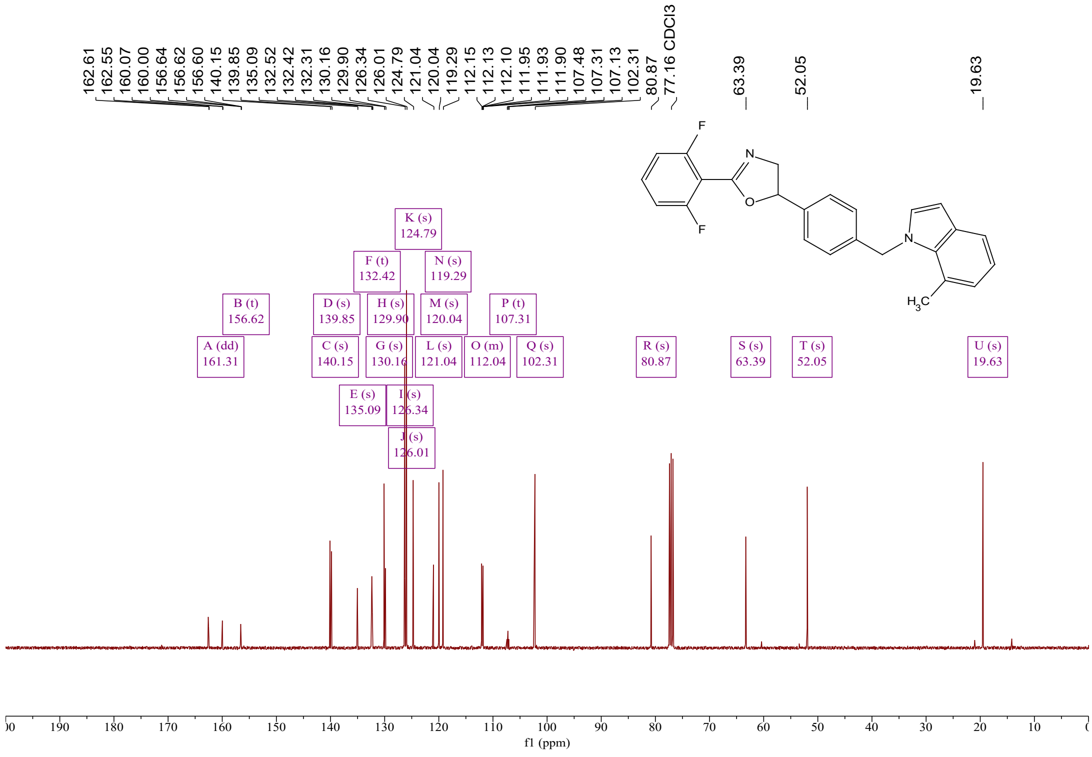

^{13}C NMR spectra of target compound **11c**

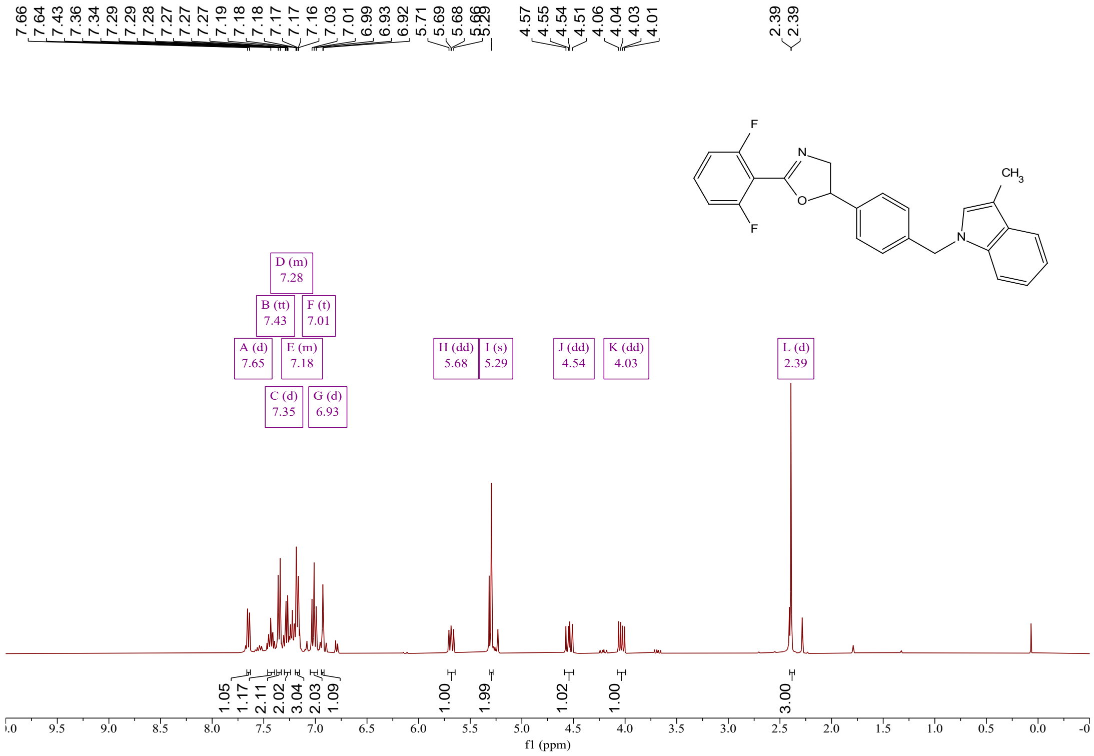

^{1}H NMR spectra of target compound **11d**

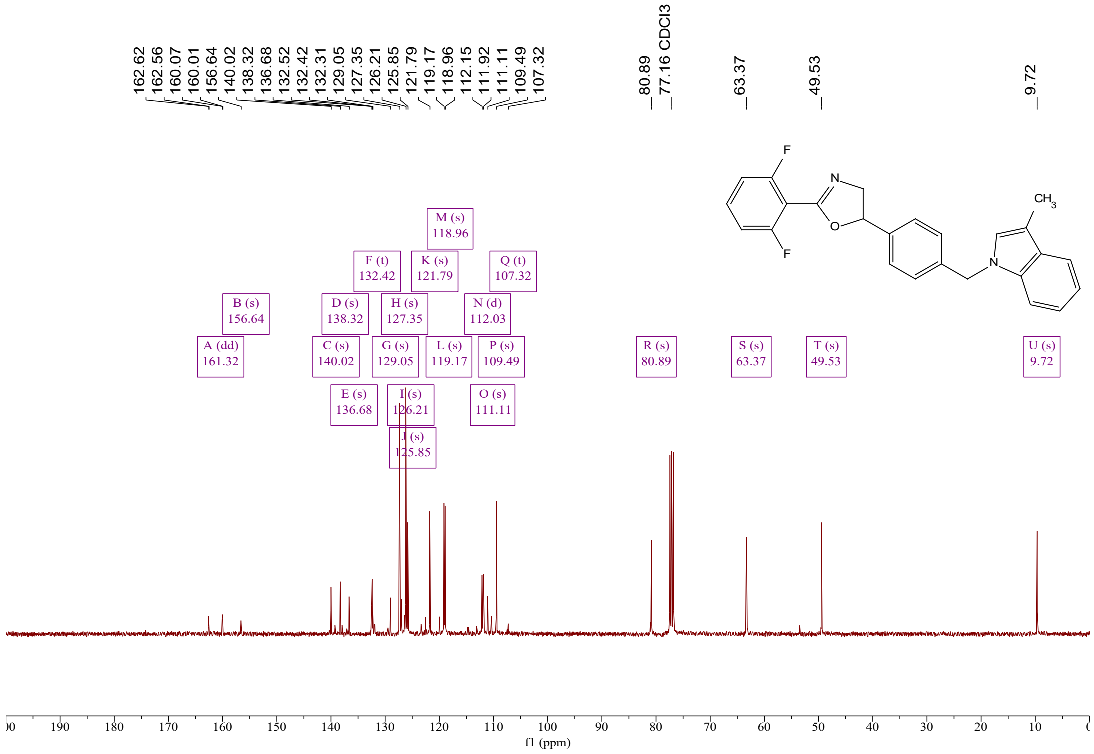

^{13}C NMR spectra of target compound **11d**

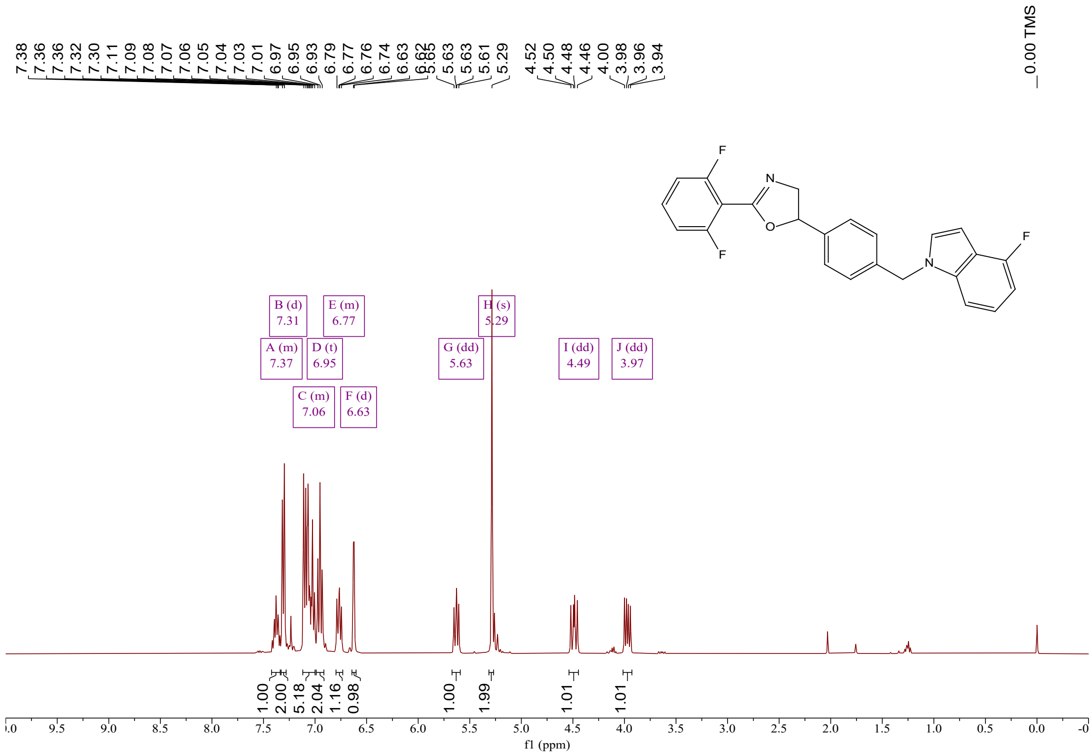

^{1}H NMR spectra of target compound **11e**

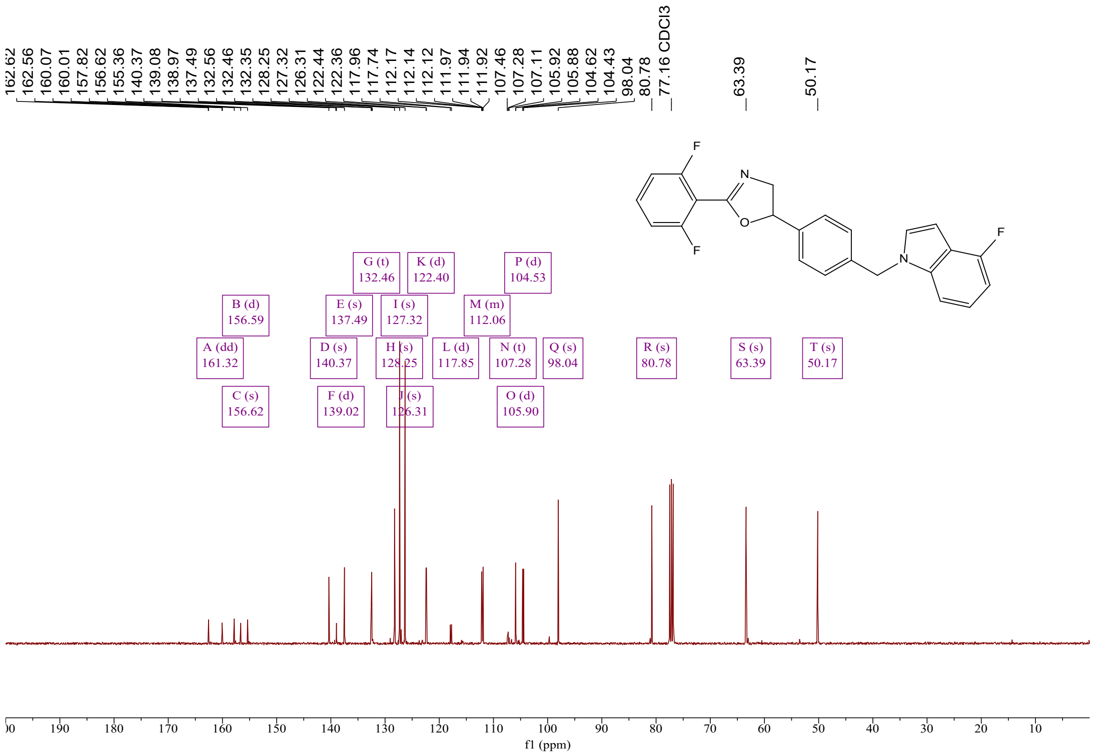

^{13}C NMR spectra of target compound **11e**

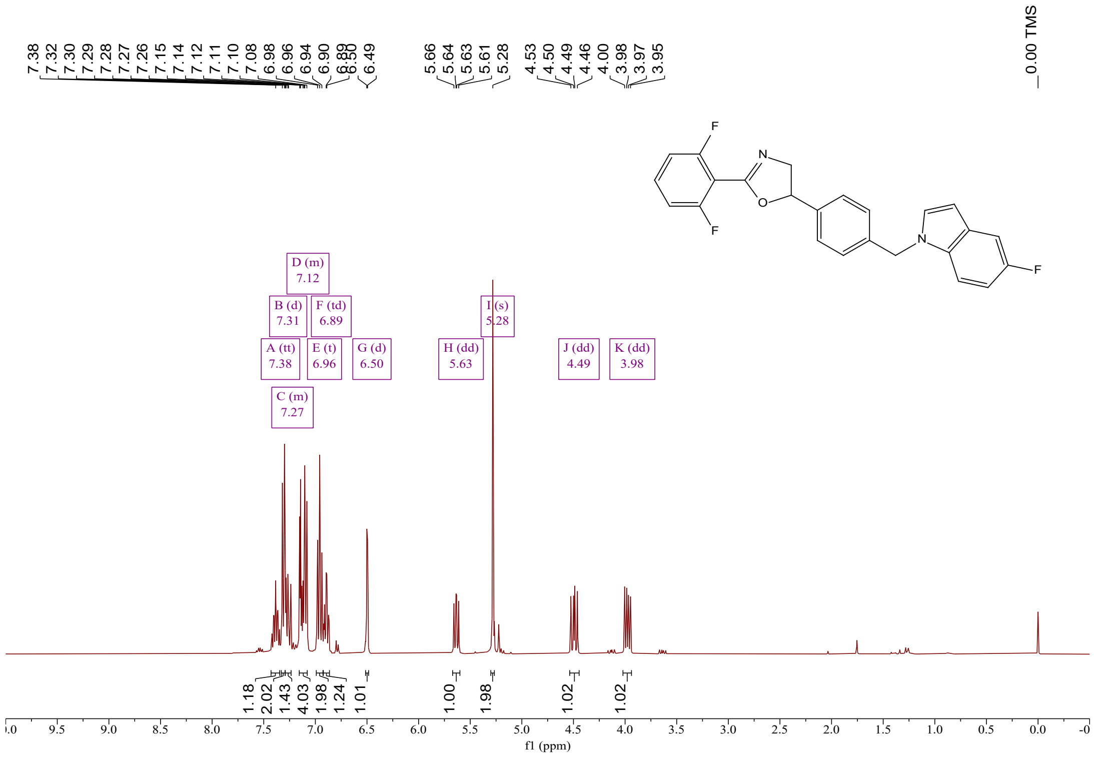

^{1}H NMR spectra of target compound **11f**

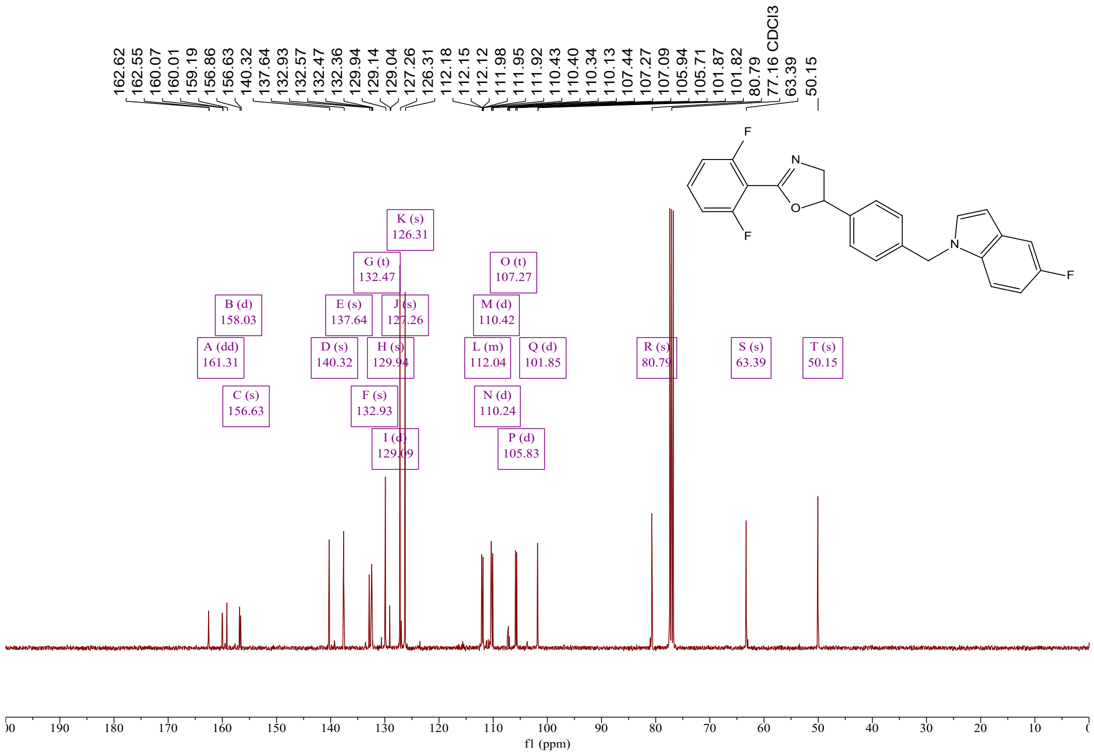

^{13}C NMR spectra of target compound **11f**

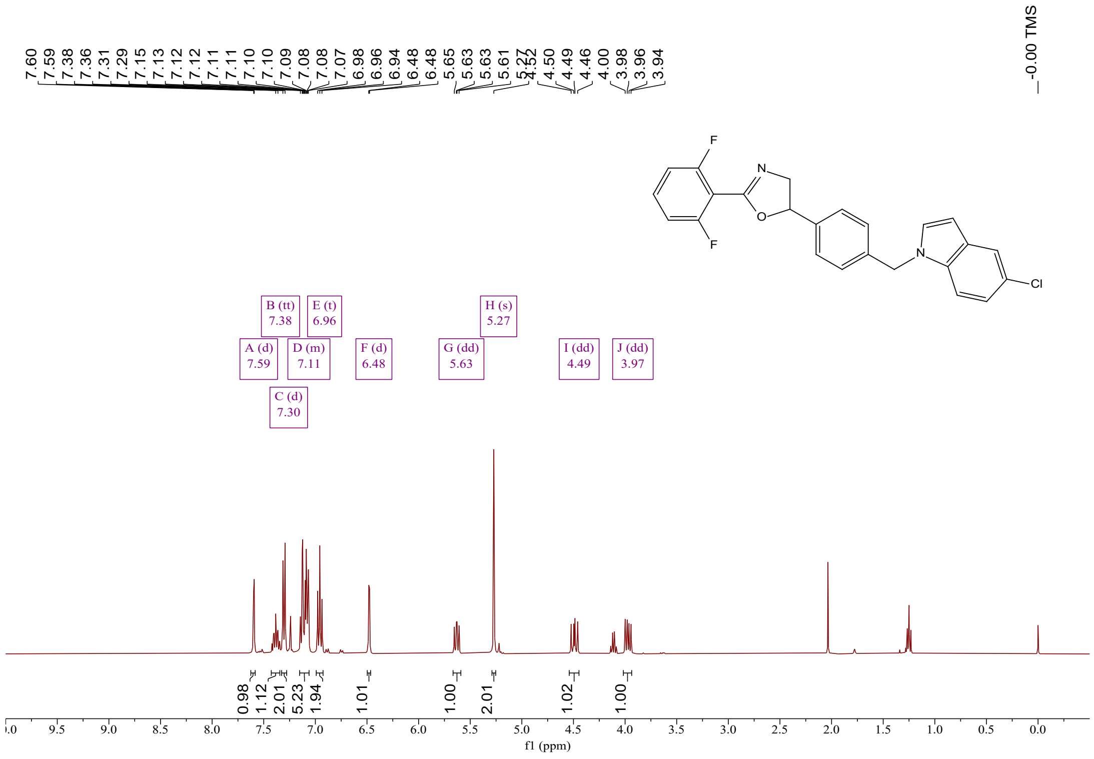

^{1}H NMR spectra of target compound **11g**

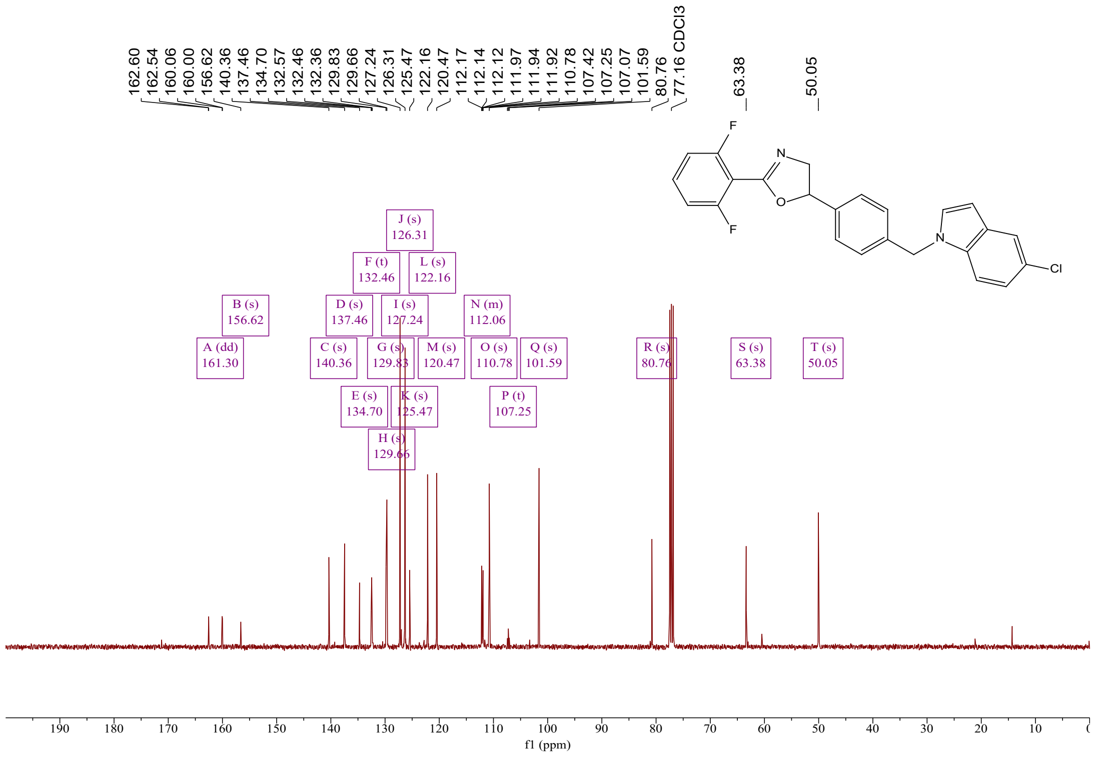

^{13}C NMR spectra of target compound **11g**

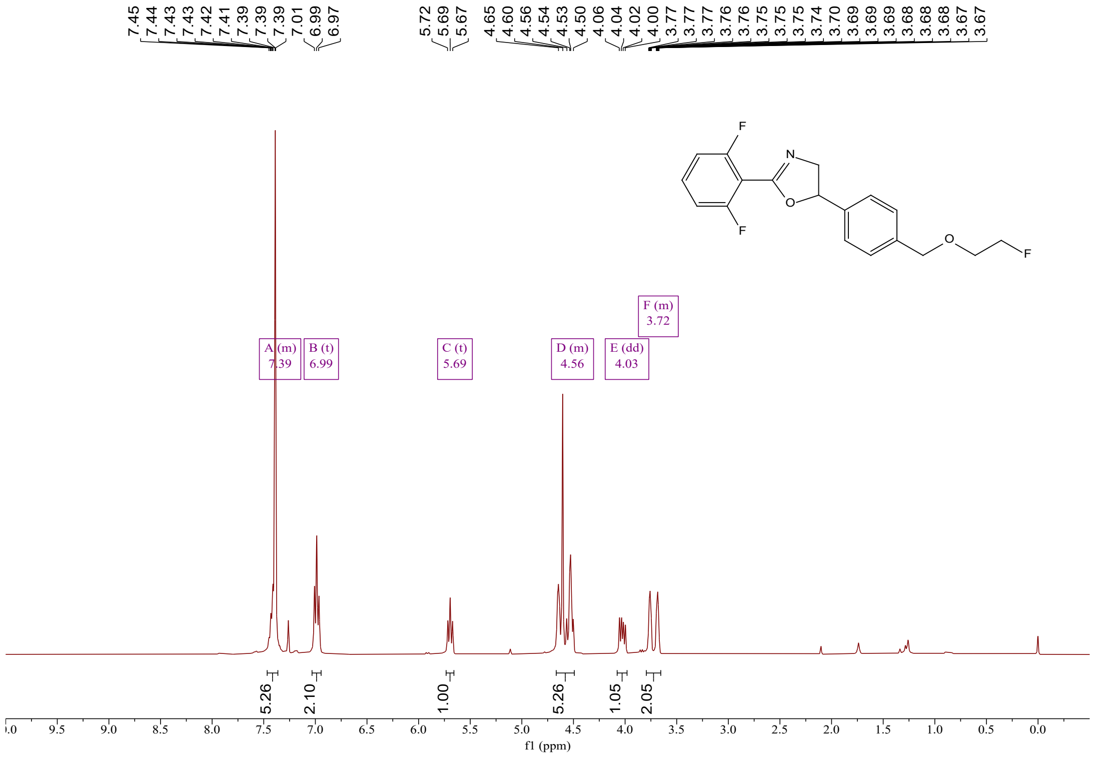

^{1}H NMR spectra of target compound **11h**

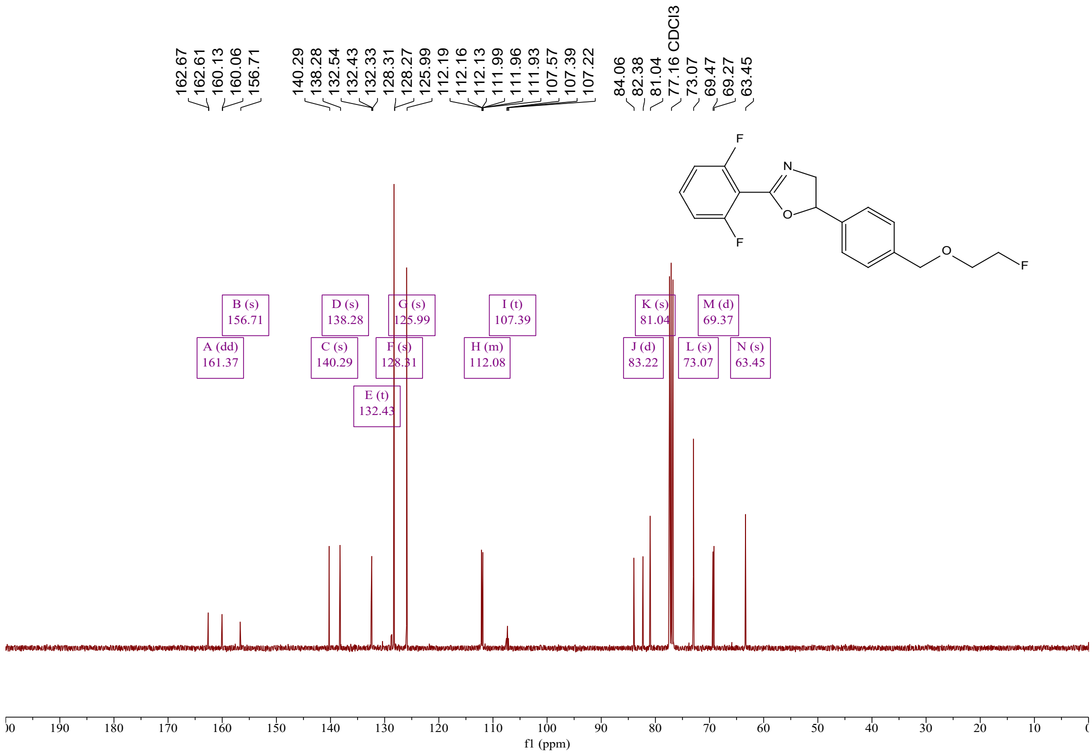

^{13}C NMR spectra of target compound **11h**

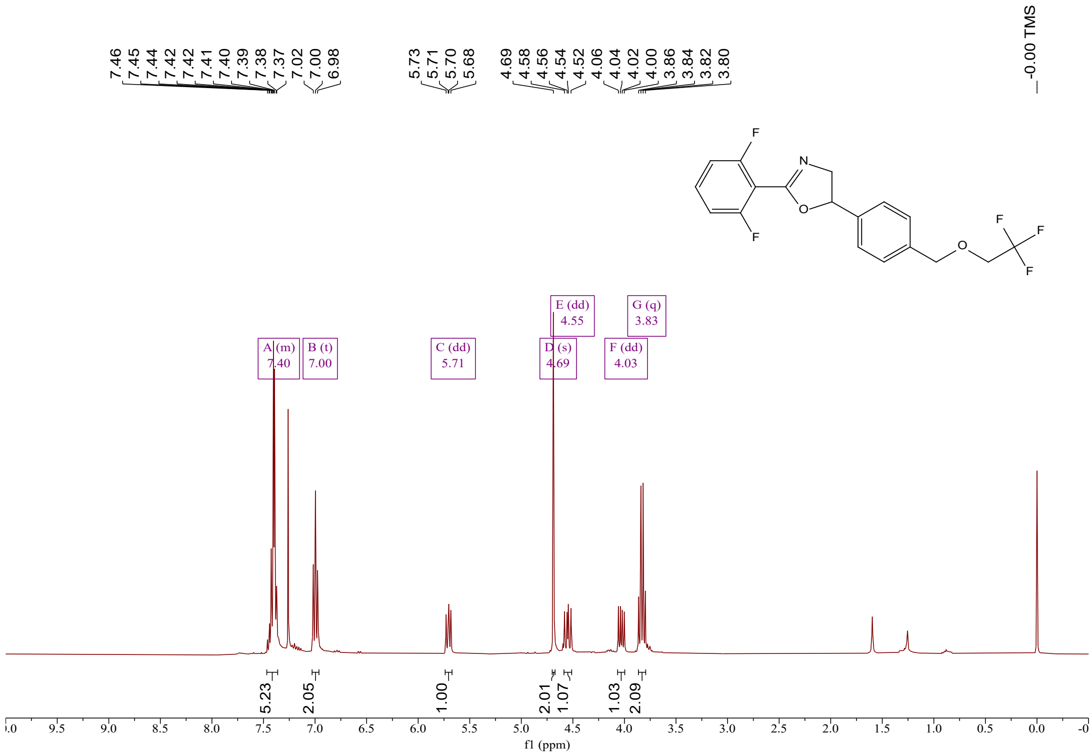

^{1}H NMR spectra of target compound **11i**

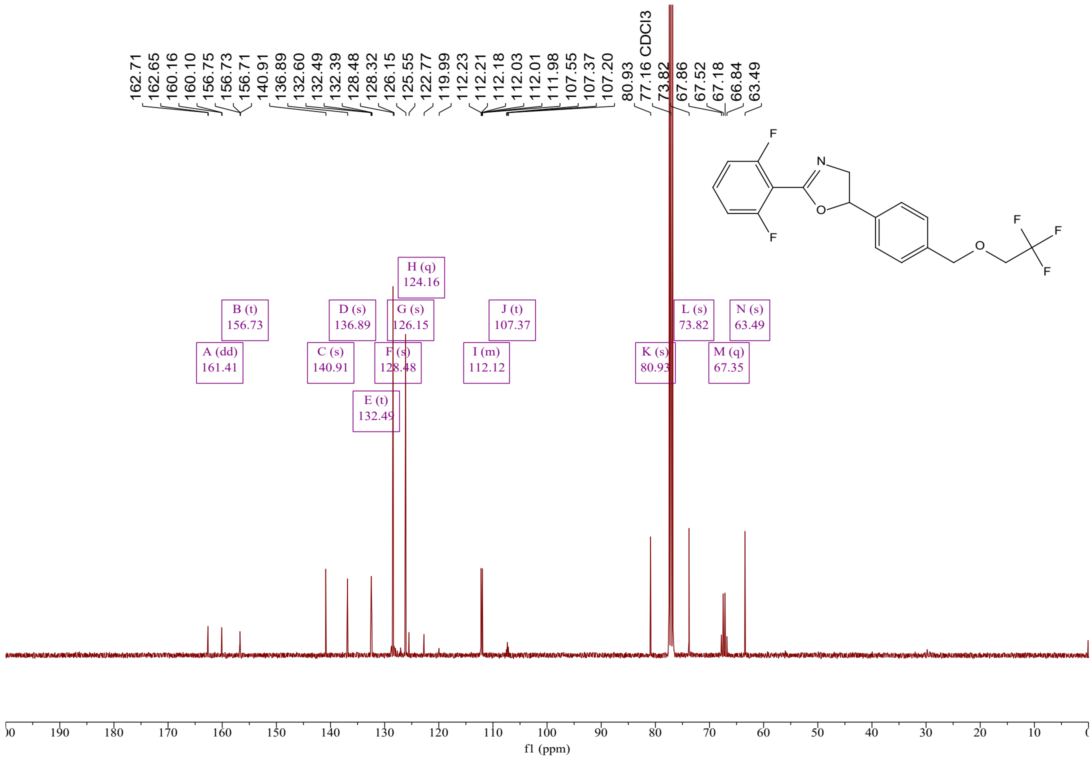

^{13}C NMR spectra of target compound **11i**

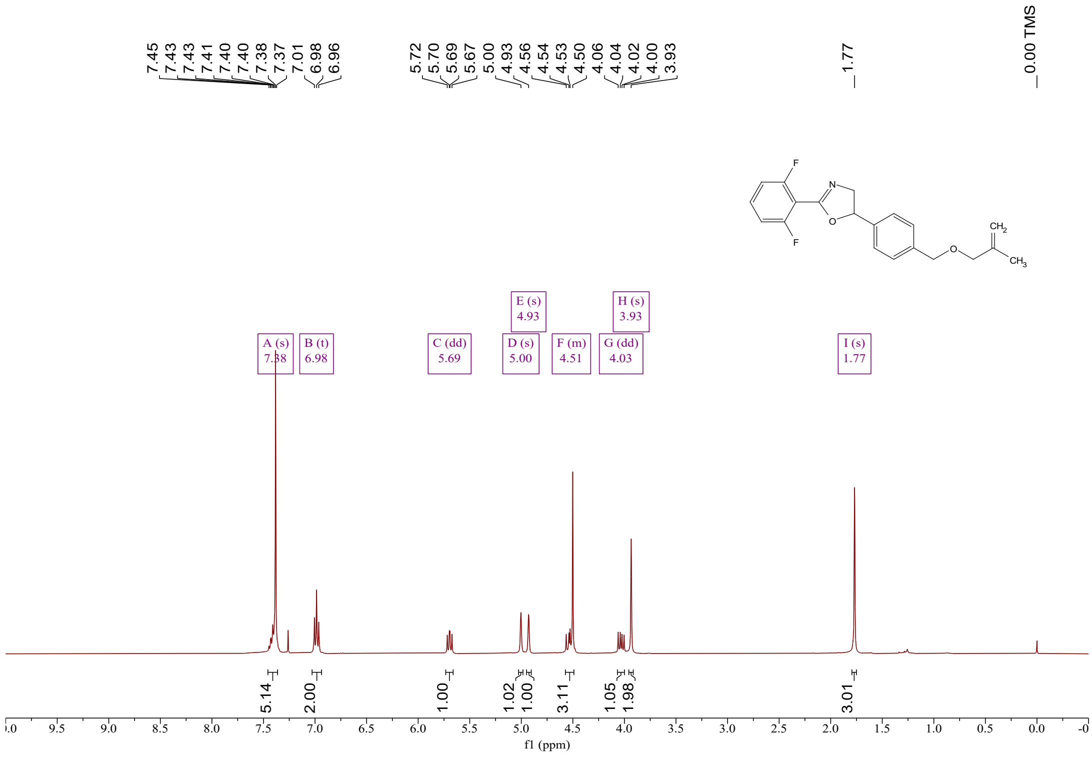

^{1}H NMR spectra of target compound **11j**

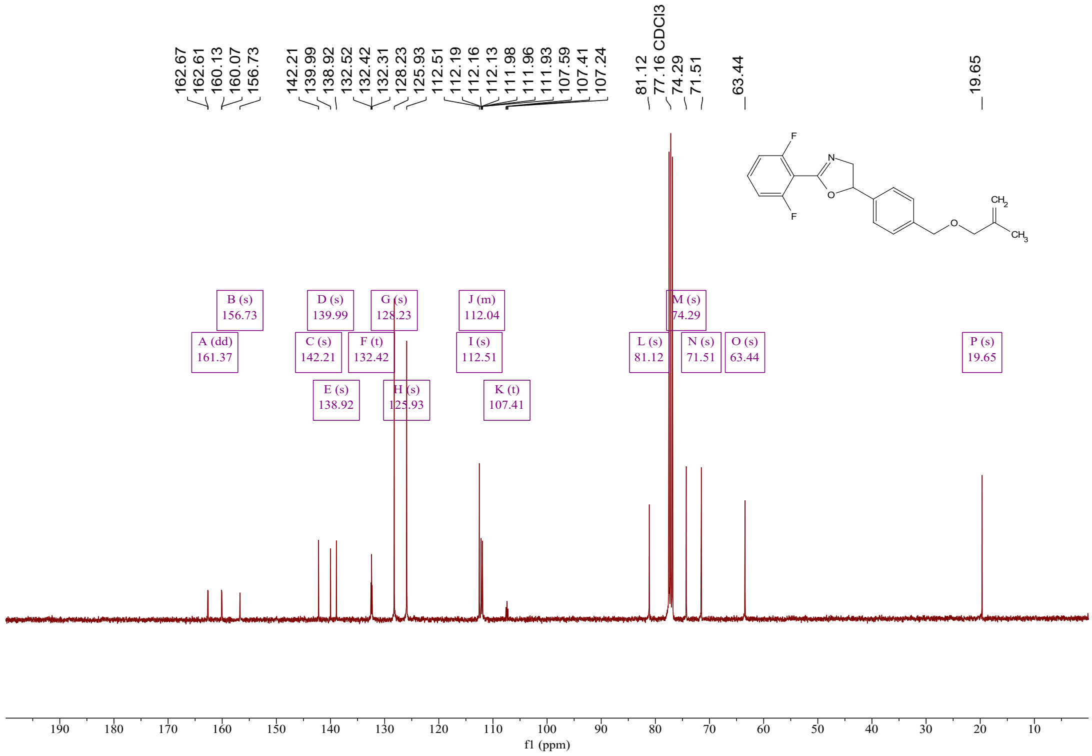

^{13}C NMR spectra of target compound **11j**

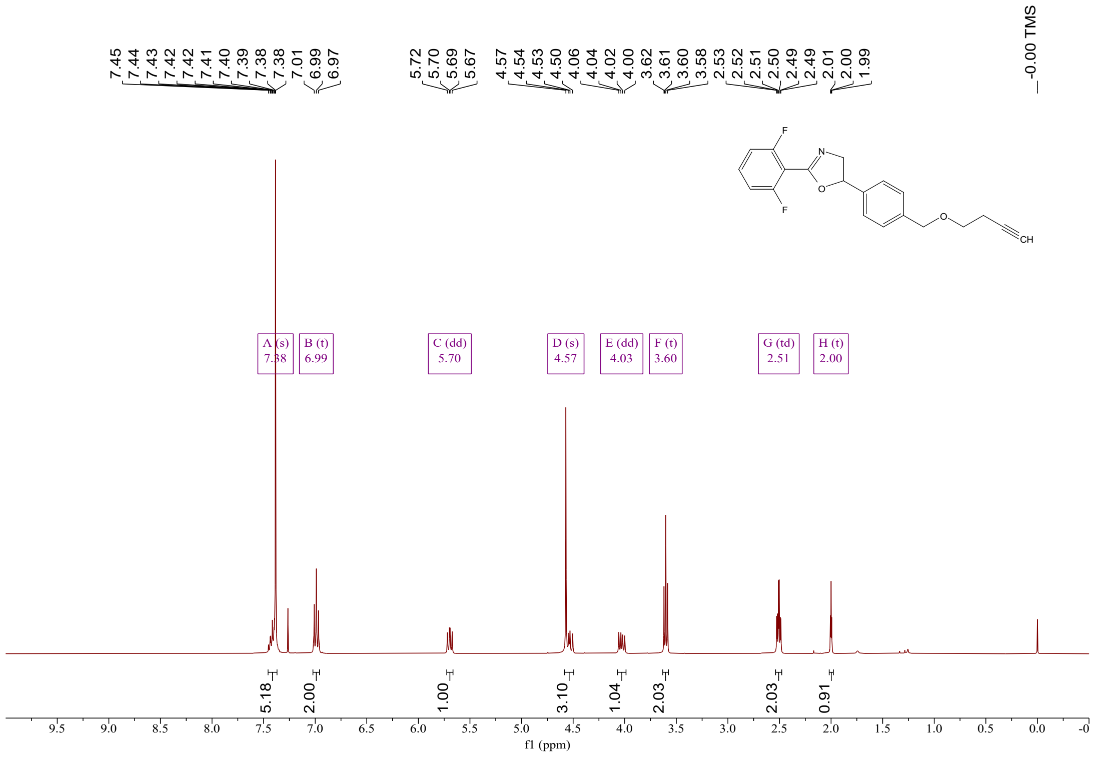

^{1}H NMR spectra of target compound **11k**

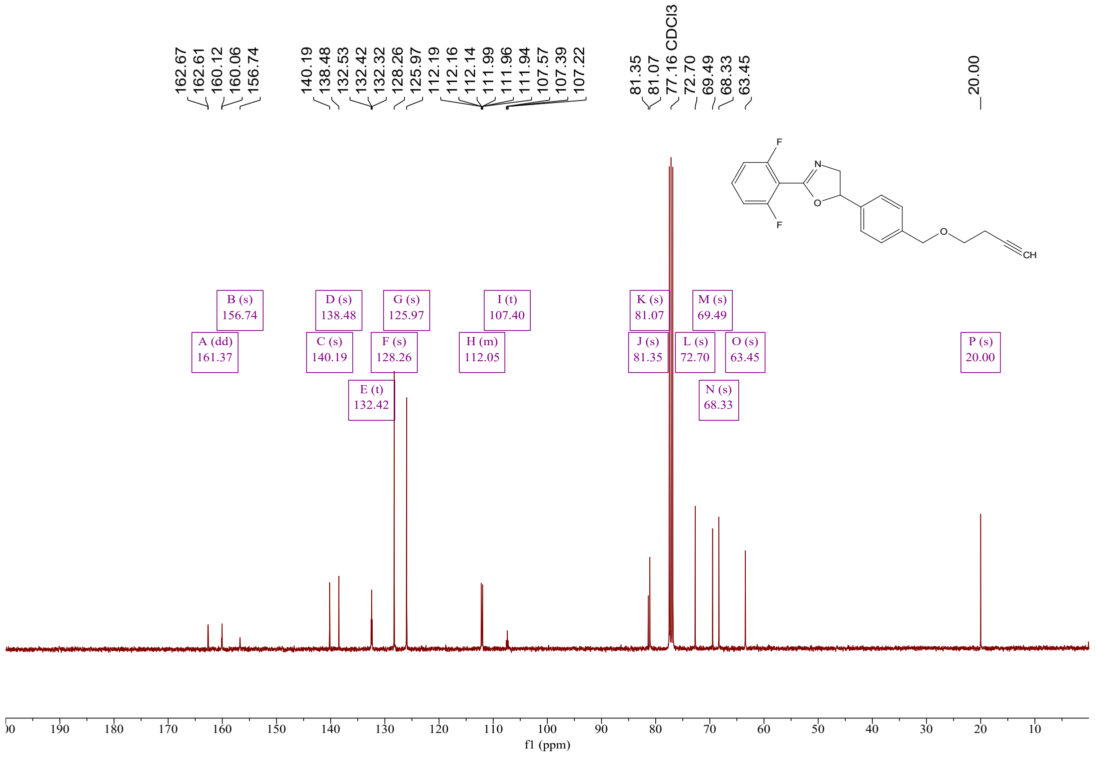

^{13}C NMR spectra of target compound **11k**

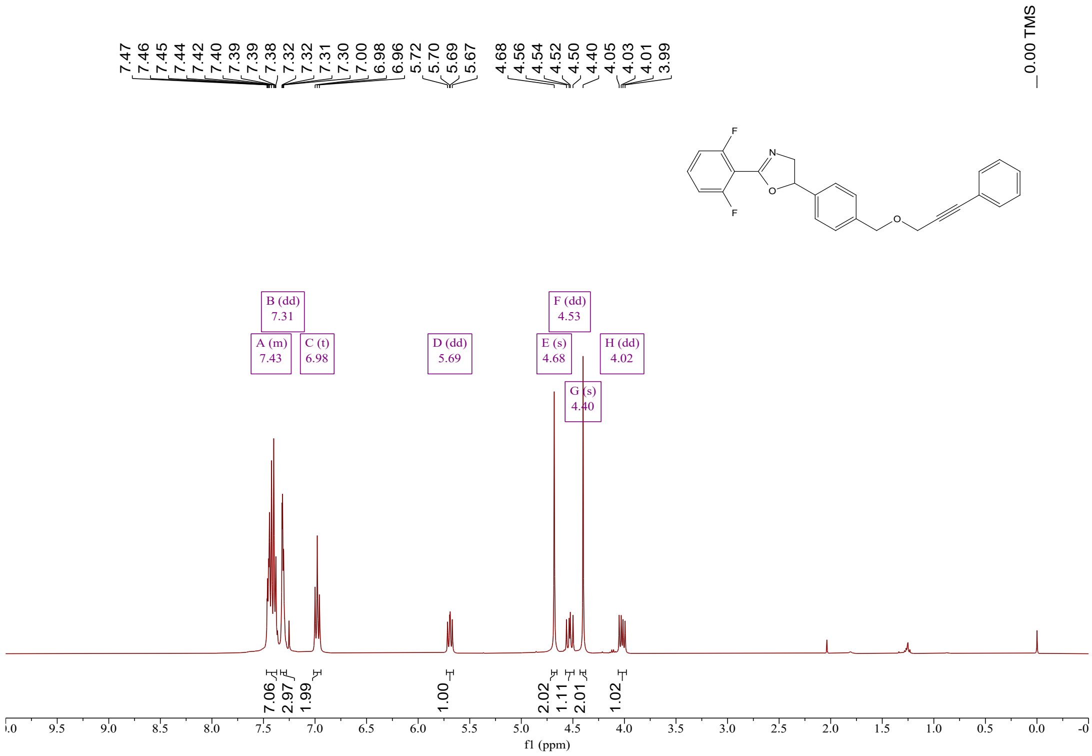

^{1}H NMR spectra of target compound **11l**

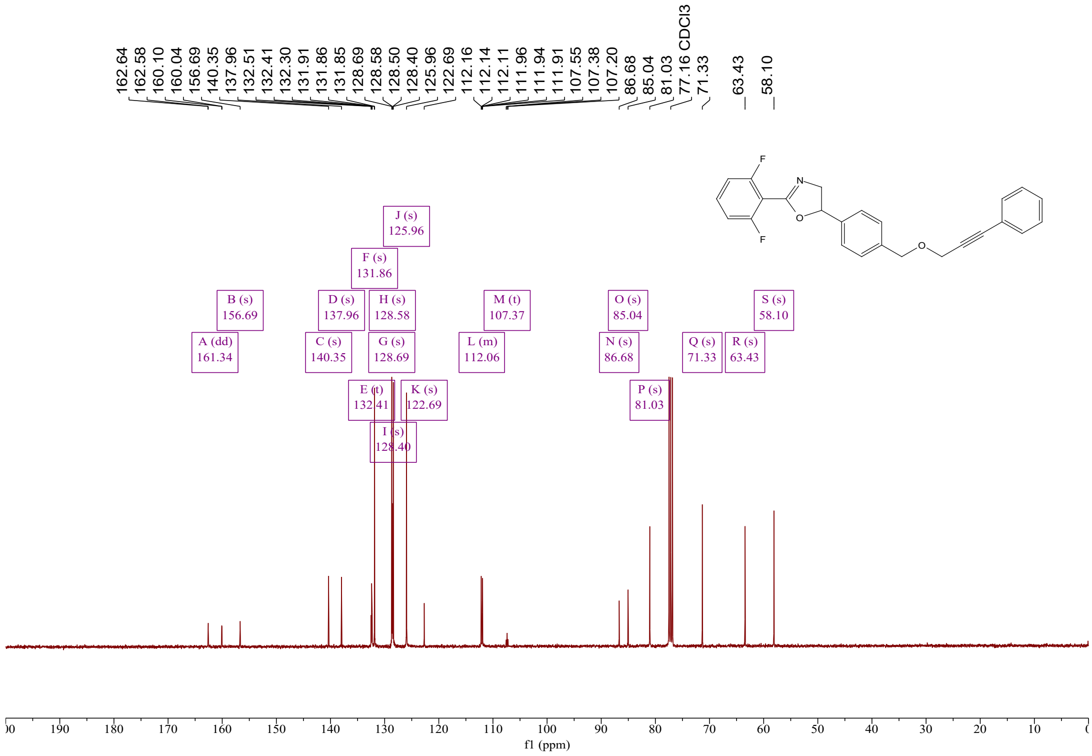

^{13}C NMR spectra of target compound **11l**

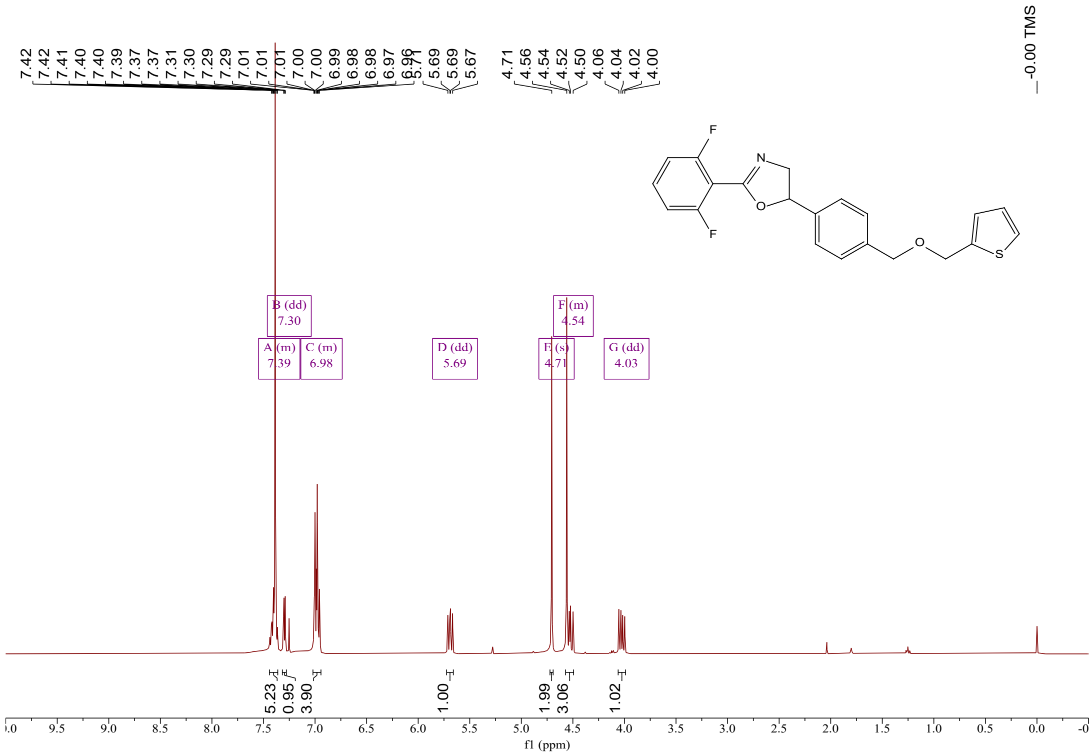

^{1}H NMR spectra of target compound **11m**

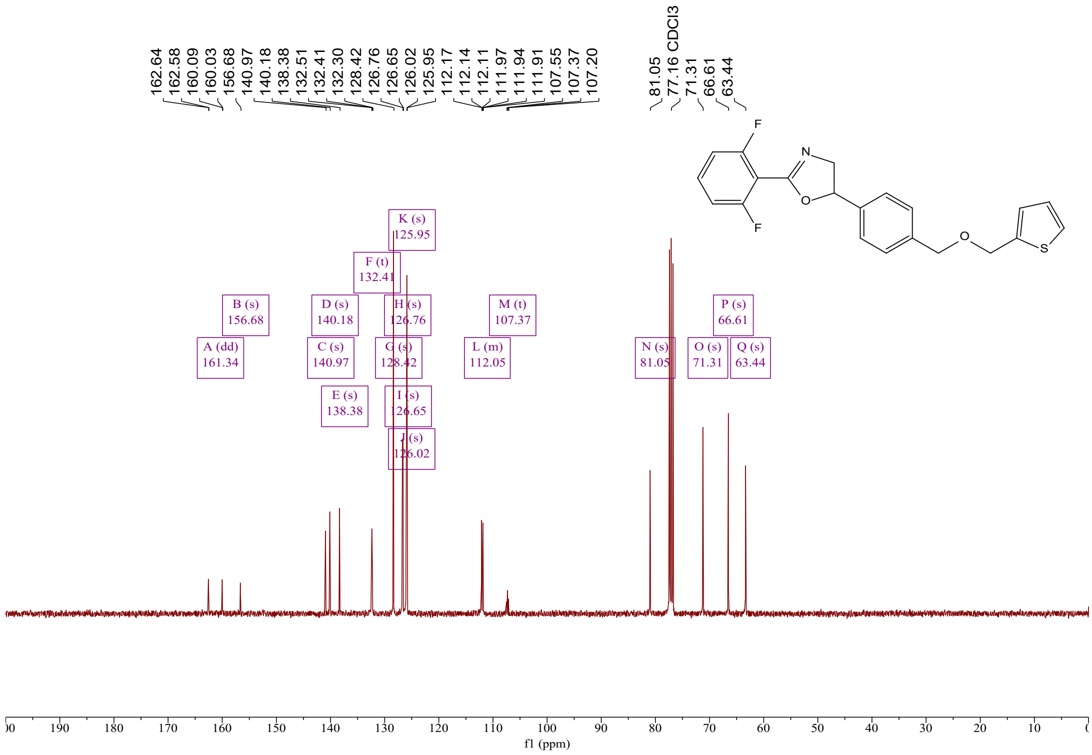

^{13}C NMR spectra of target compound **11m**

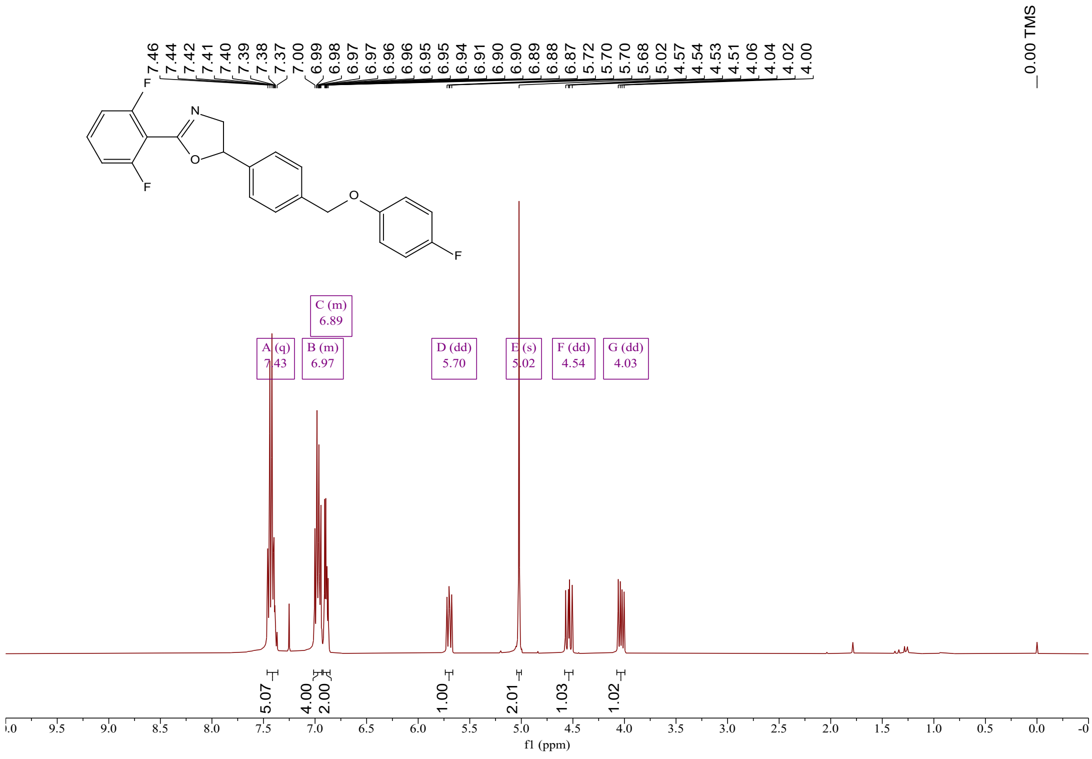

^{1}H NMR spectra of target compound **11n**

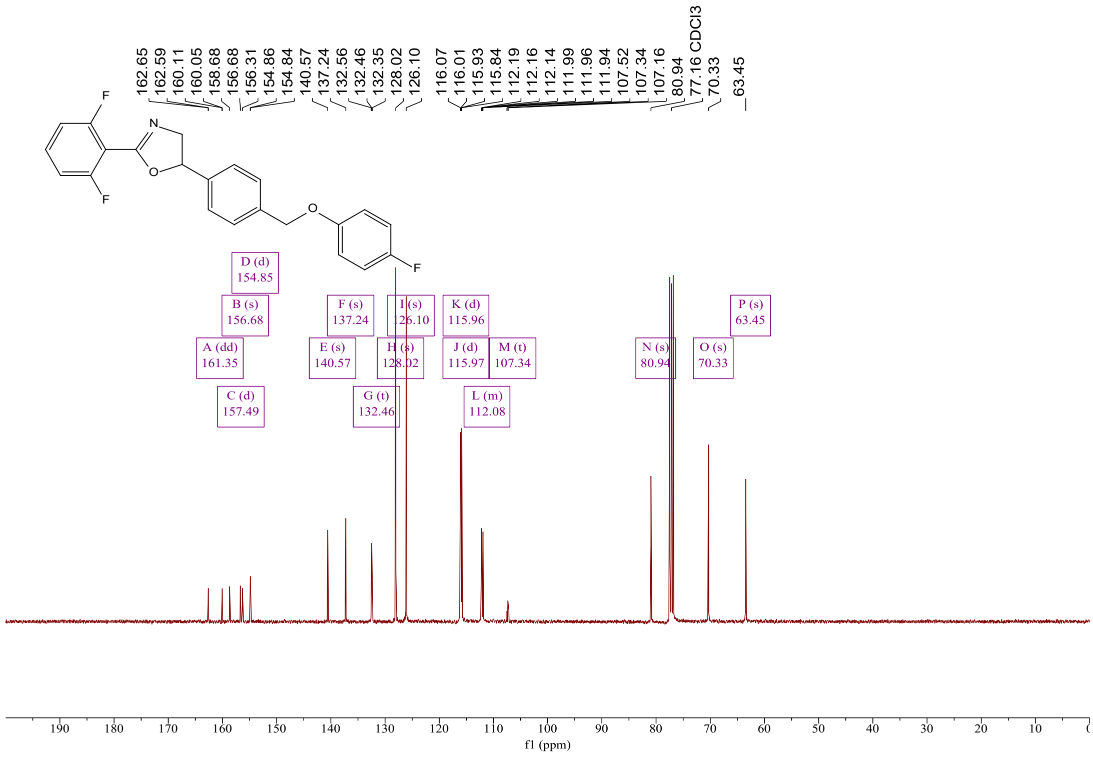

^{13}C NMR spectra of target compound **11n**

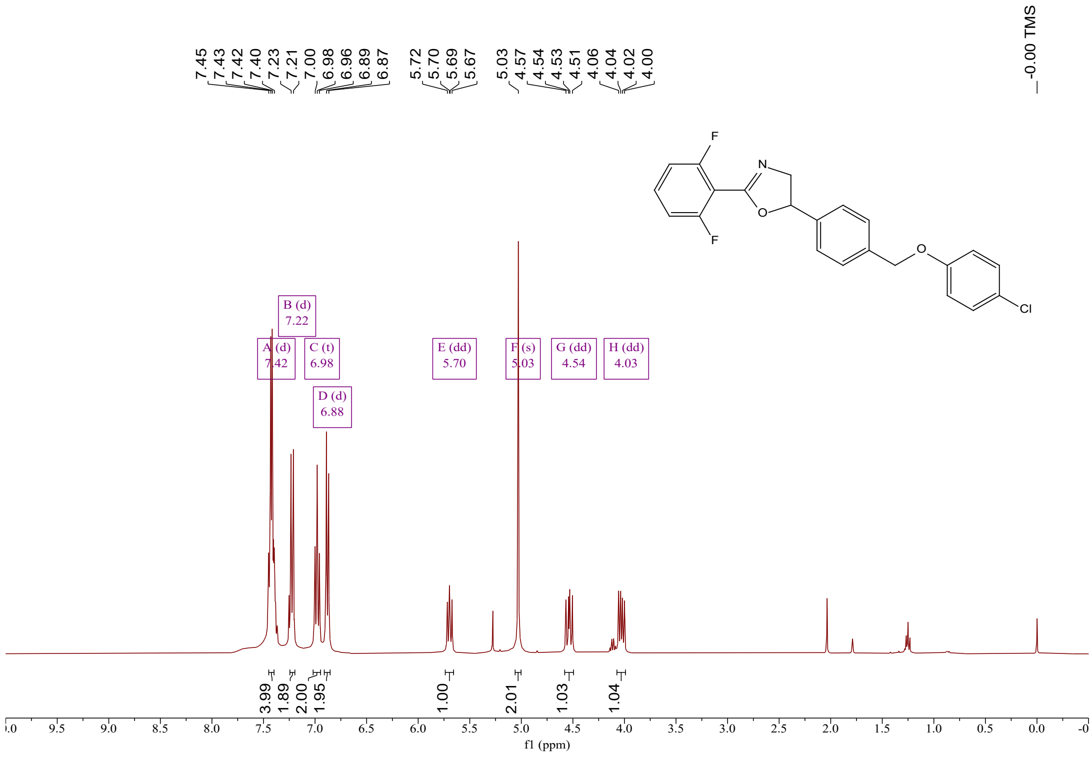

^{1}H NMR spectra of target compound **11o**

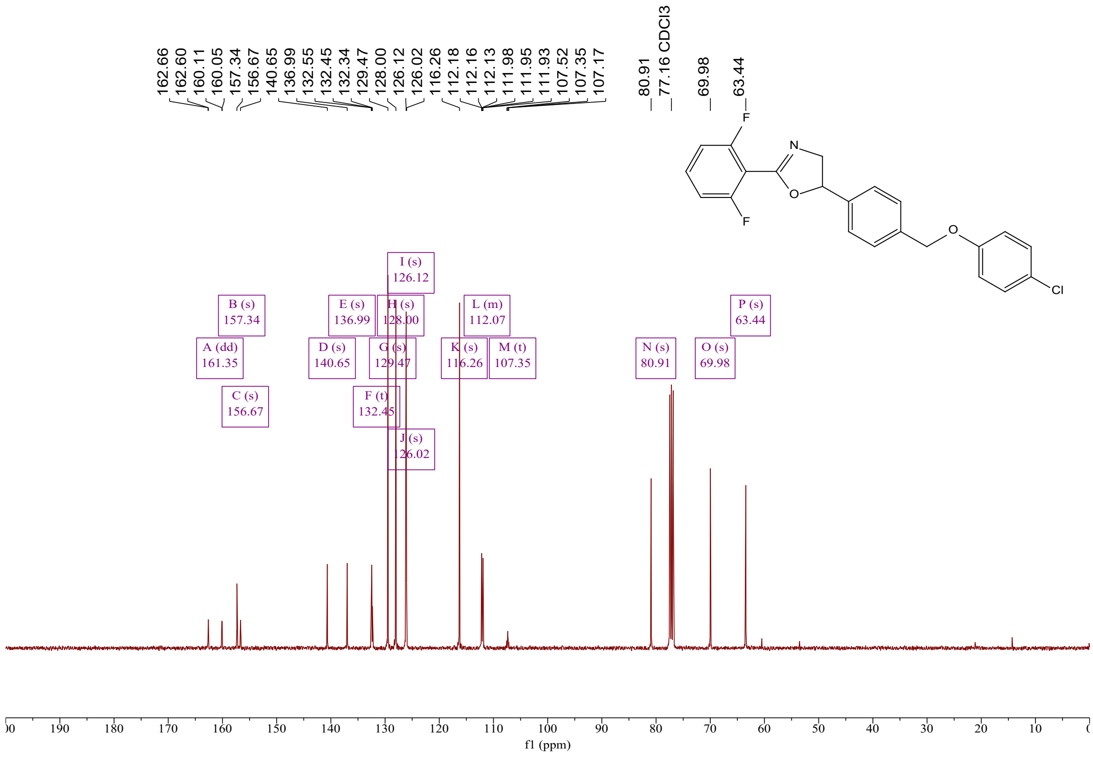

^{13}C NMR spectra of target compound **11o**

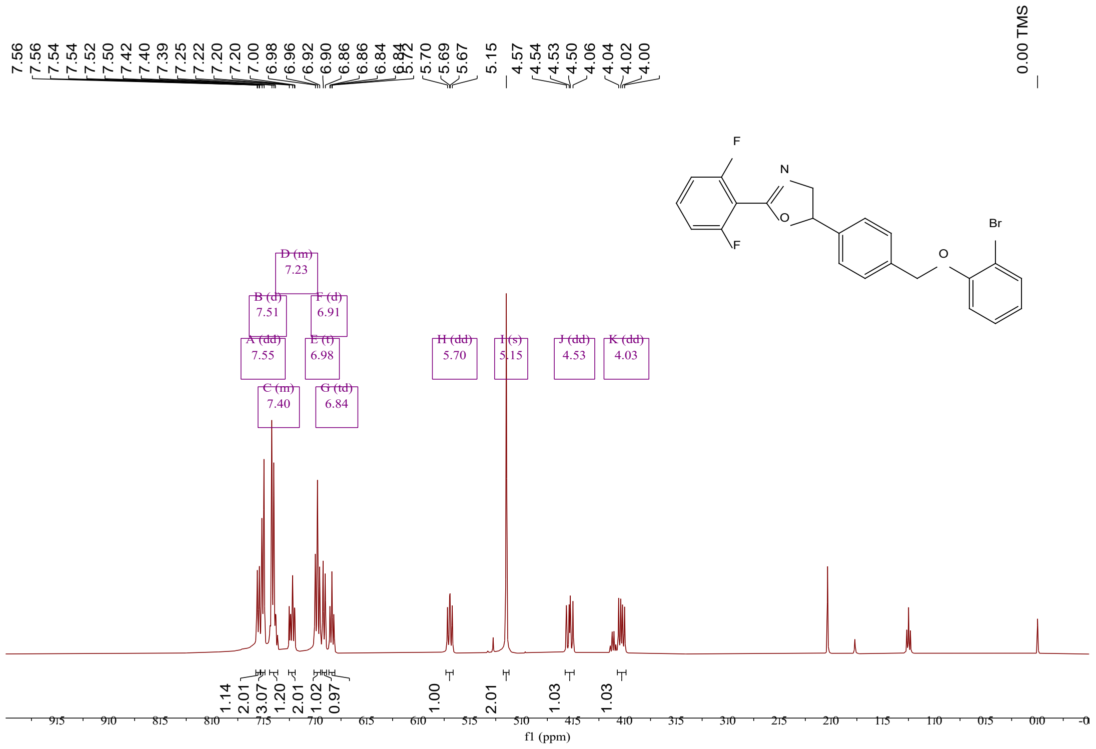

^{1}H NMR spectra of target compound **11p**

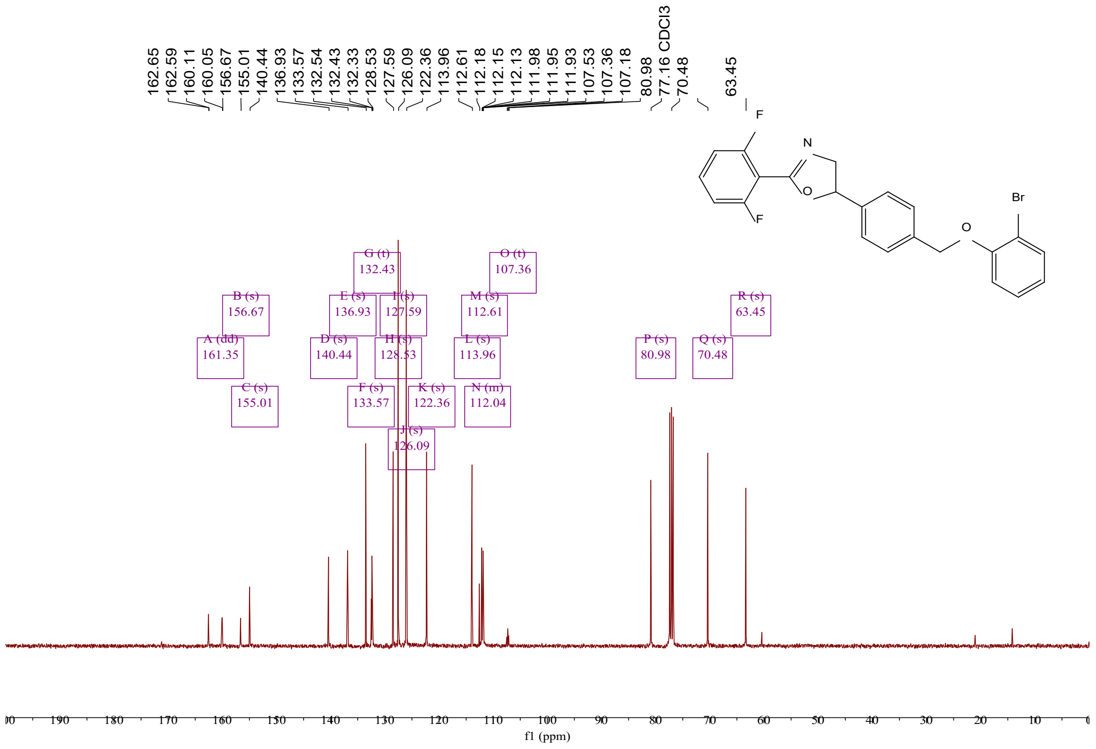

^{13}C NMR spectra of target compound **11p**

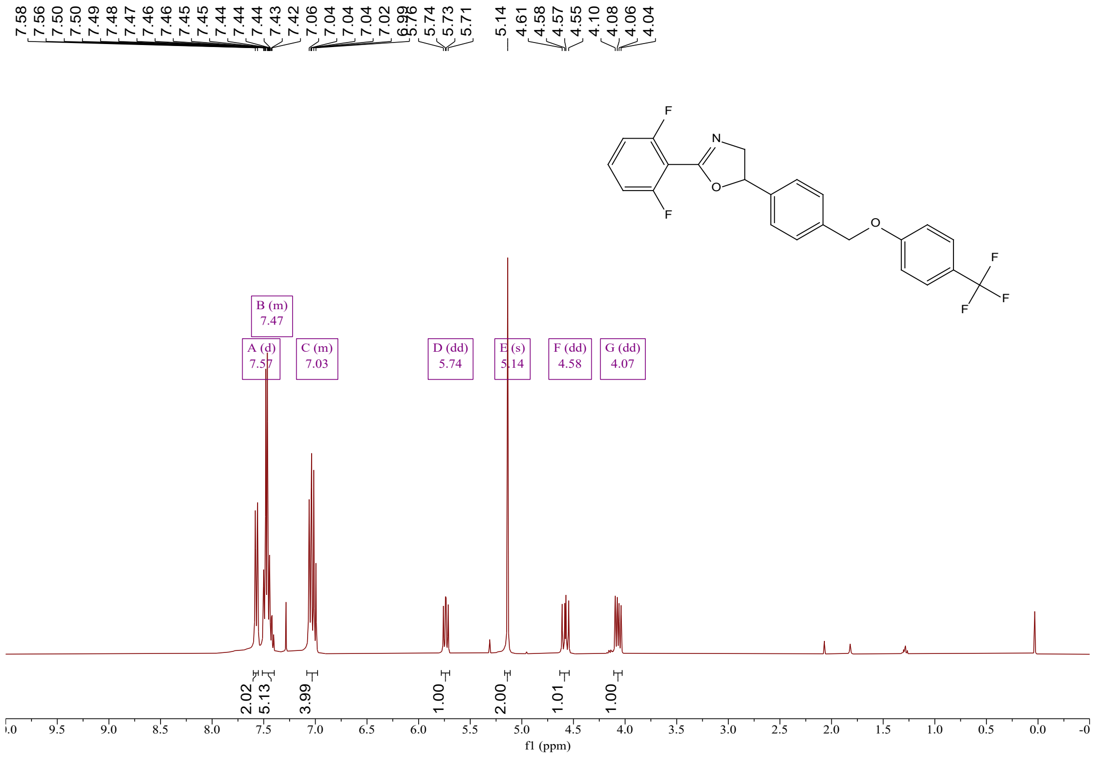

^{1}H NMR spectra of target compound **11q**

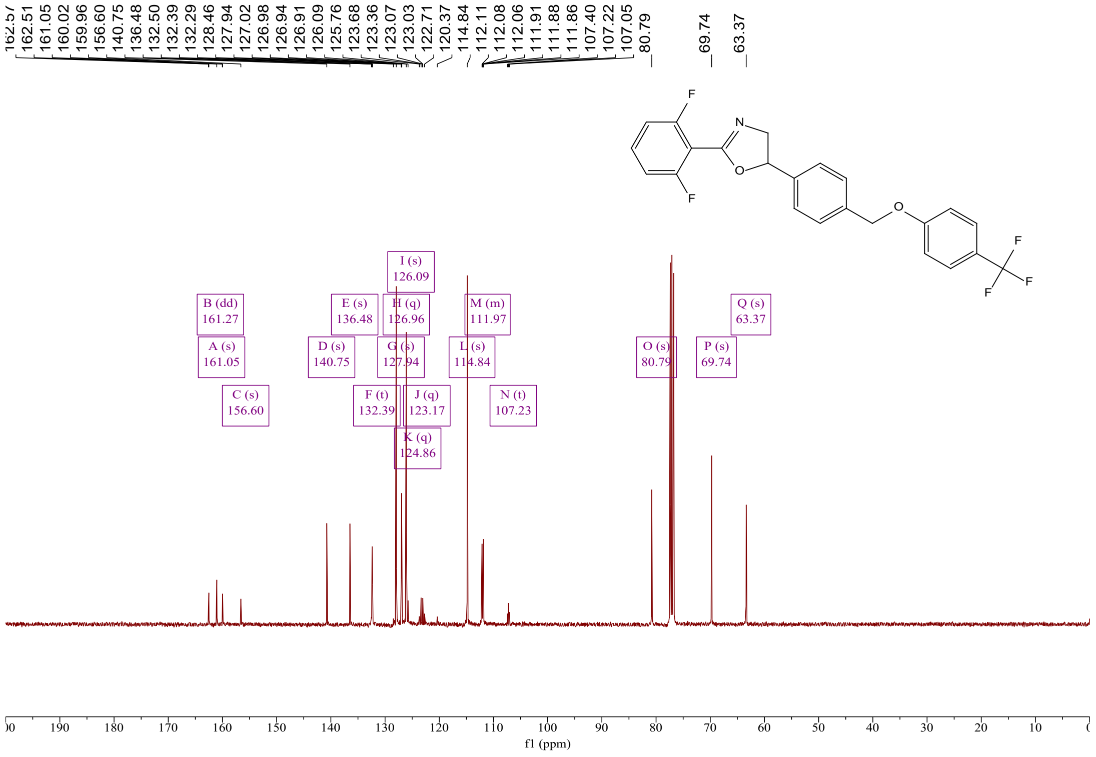

^{13}C NMR spectra of target compound **11q**

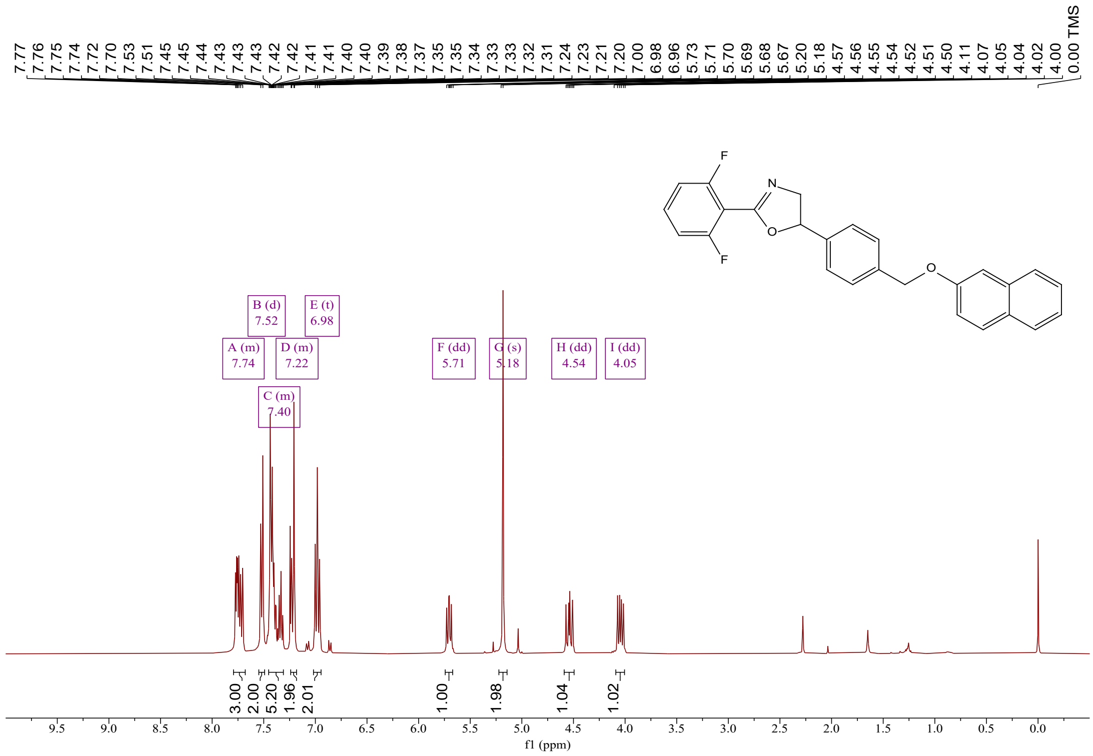

^{1}H NMR spectra of target compound **11r**

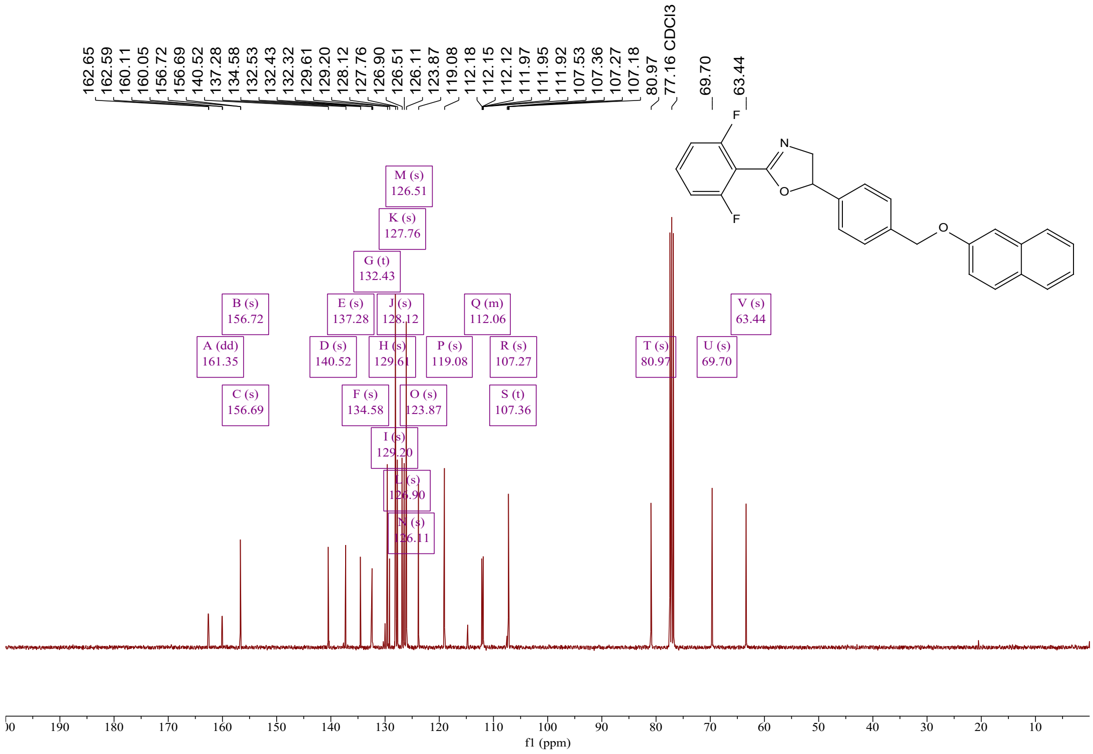

^{13}C NMR spectra of target compound **11r**

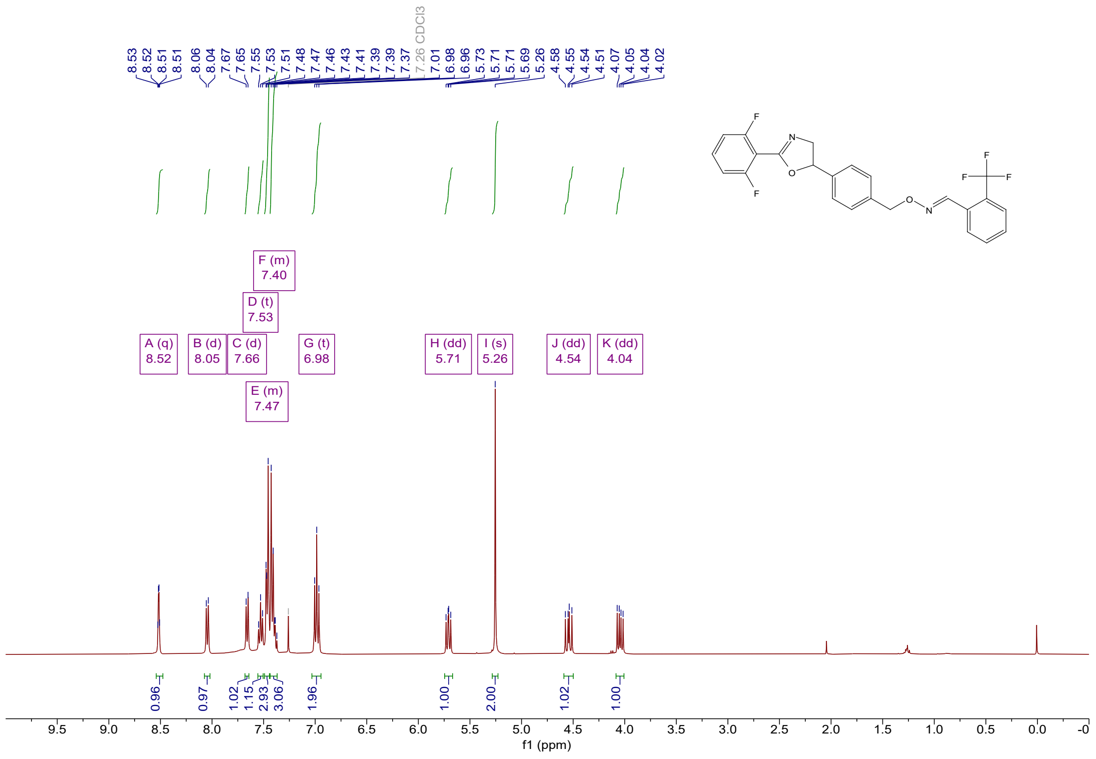

^{1}H NMR spectra of target compound **11s**

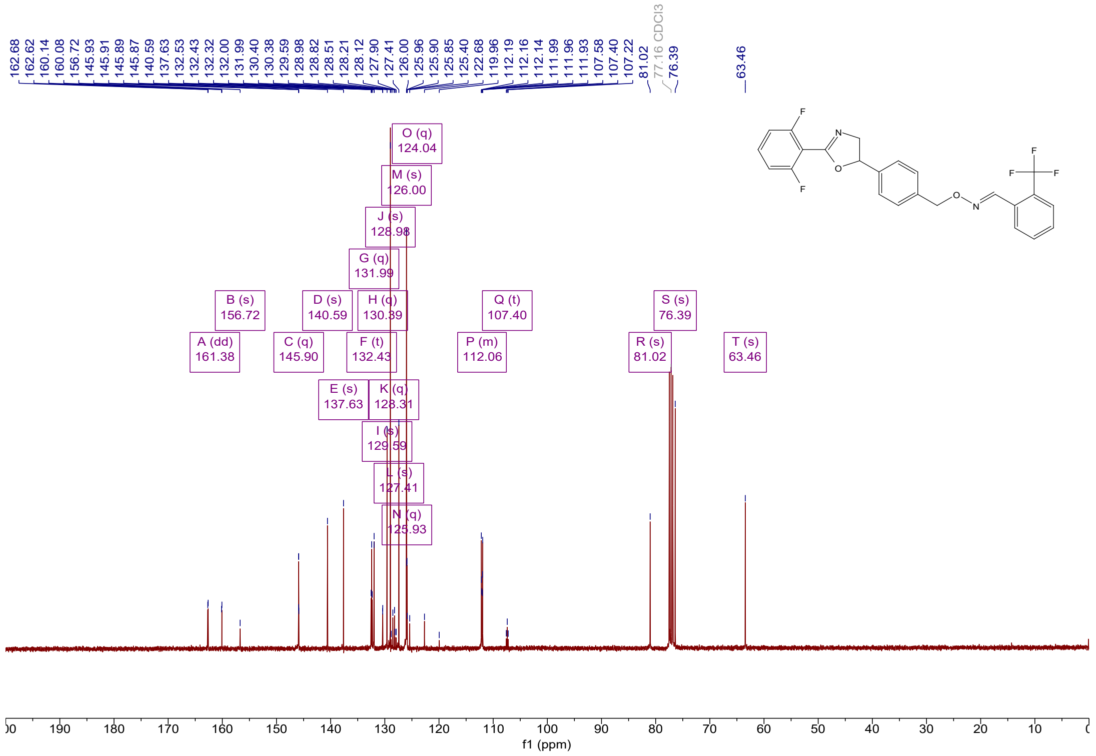

^{13}C NMR spectra of target compound **11s**

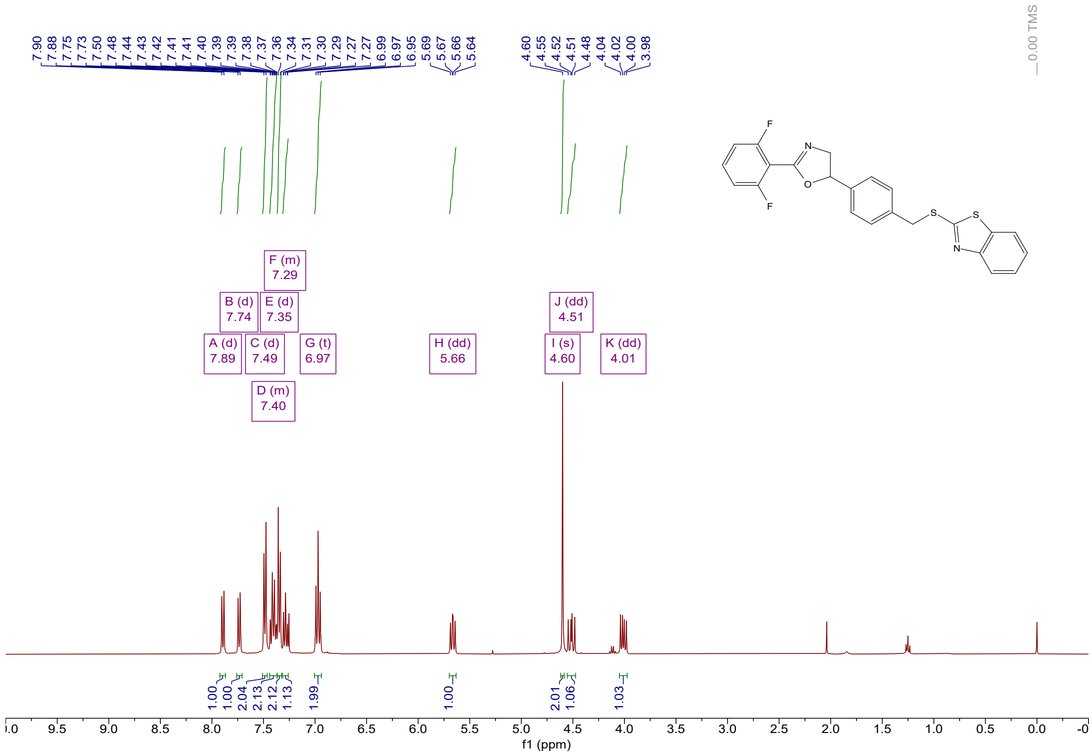

^{1}H NMR spectra of target compound **11t**

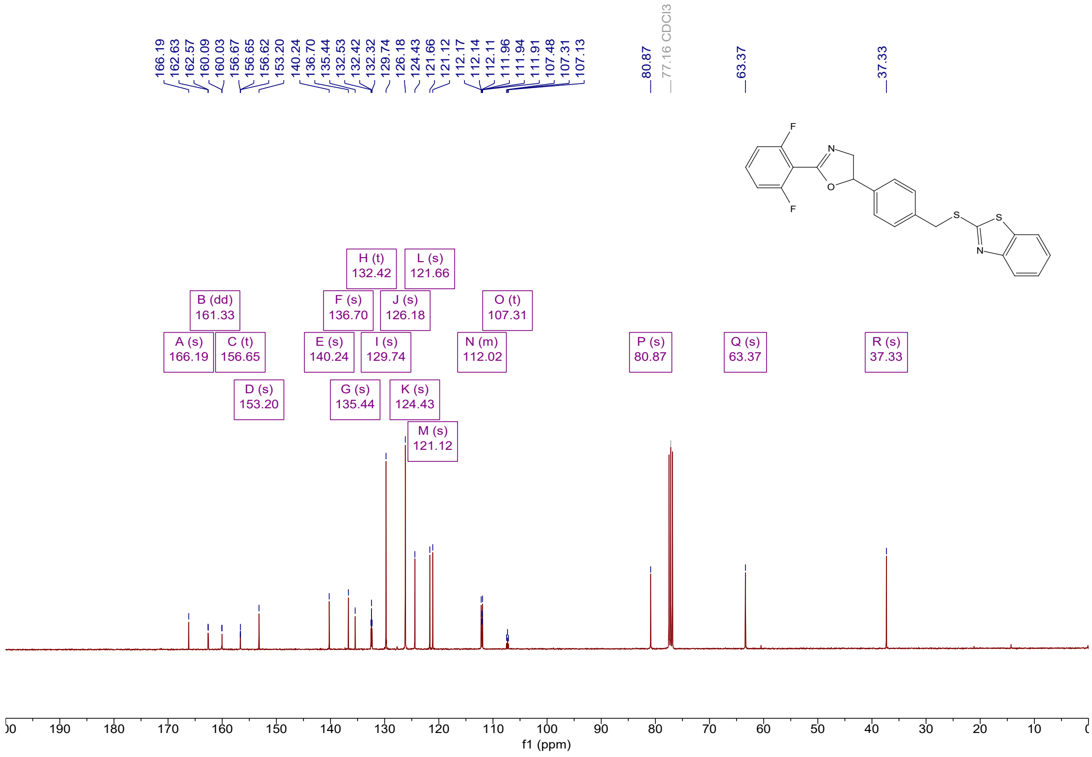

^{13}C NMR spectra of target compound **11t**

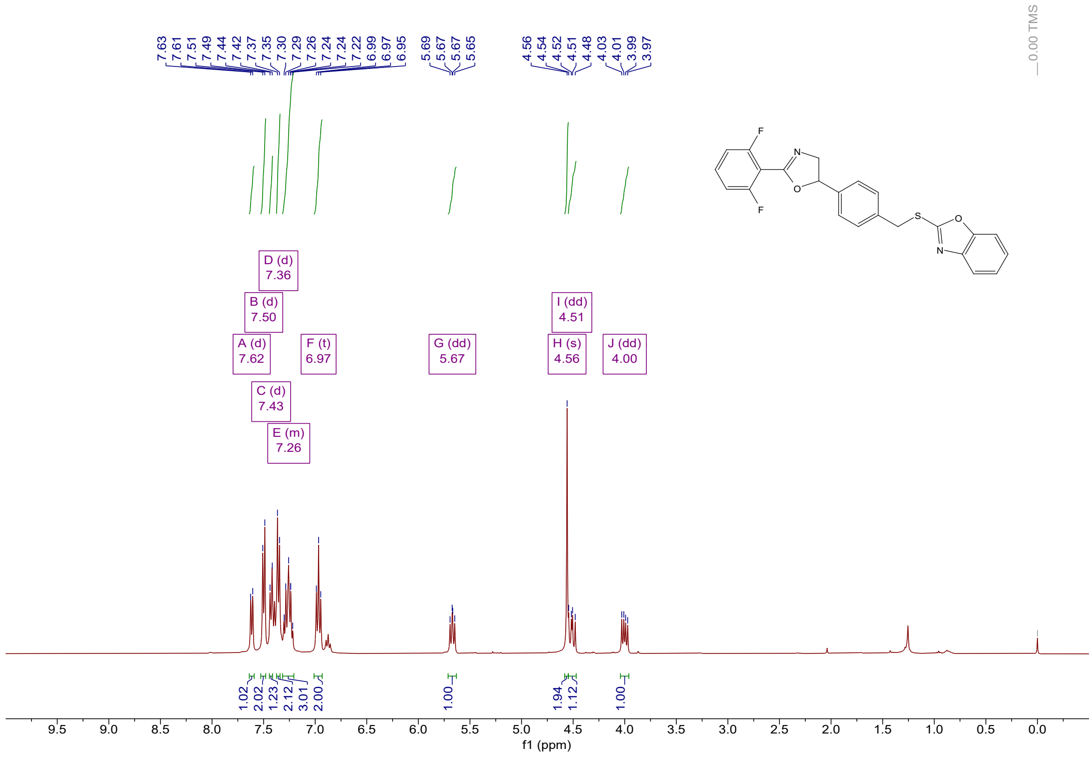

^{1}H NMR spectra of target compound **11u**

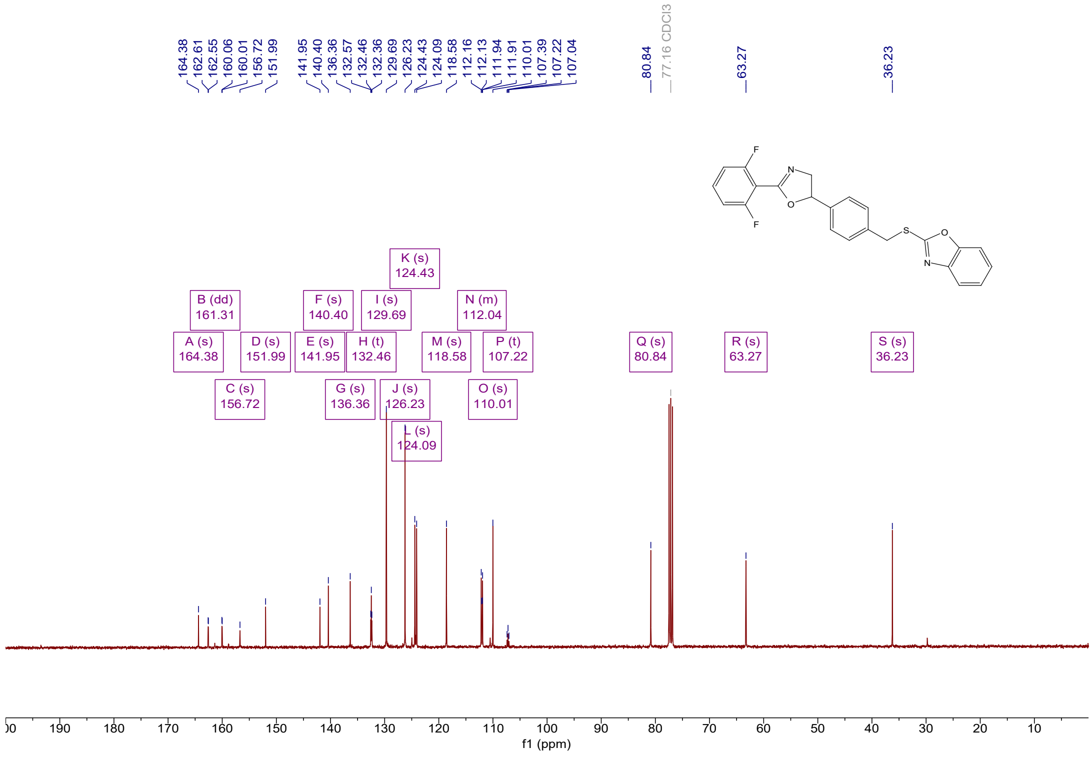

^{13}C NMR spectra of target compound **11u**

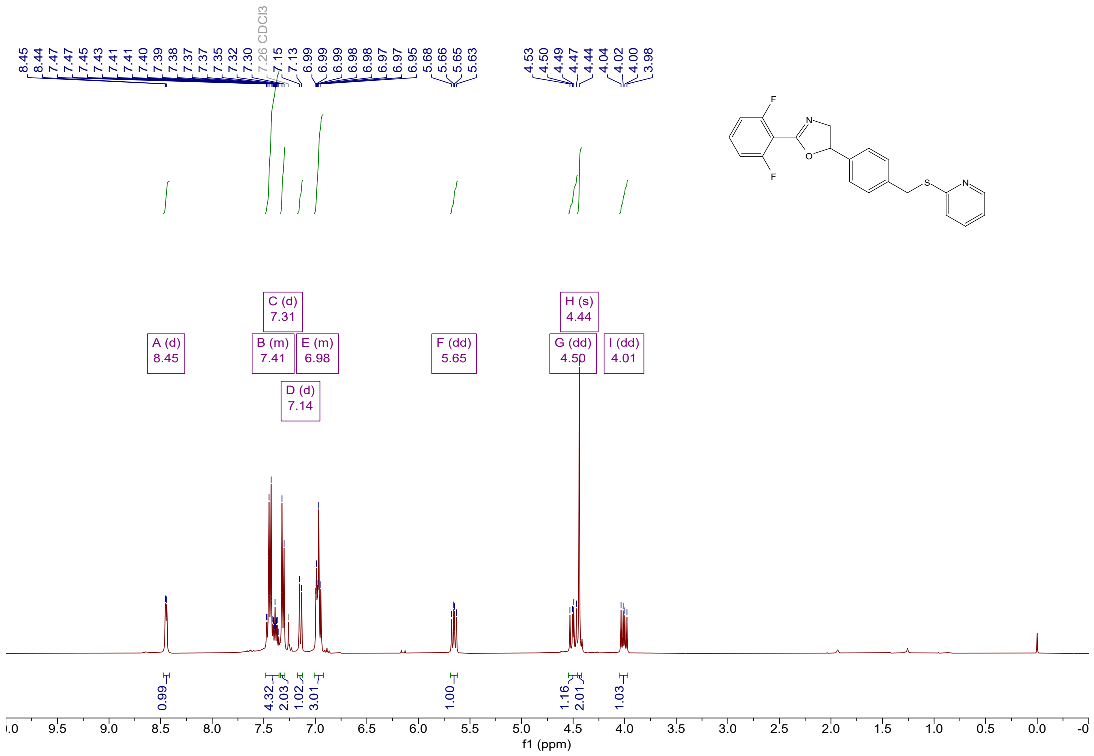

^{1}H NMR spectra of target compound **11v**

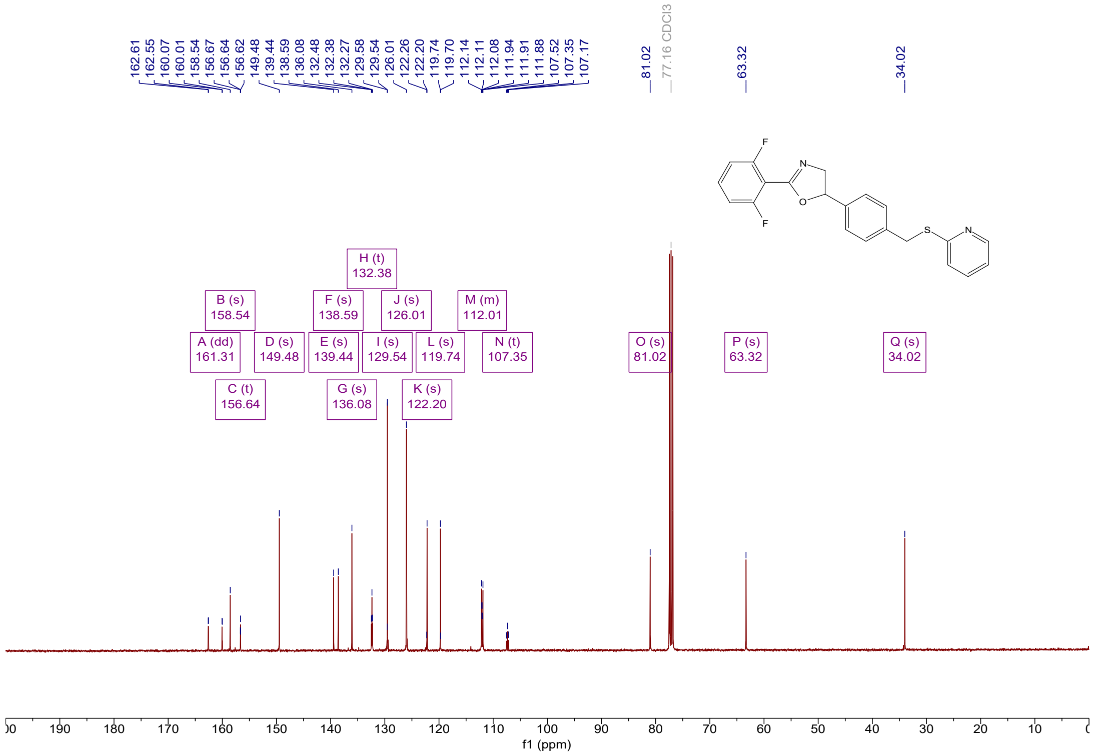

^{13}C NMR spectra of target compound **11v**

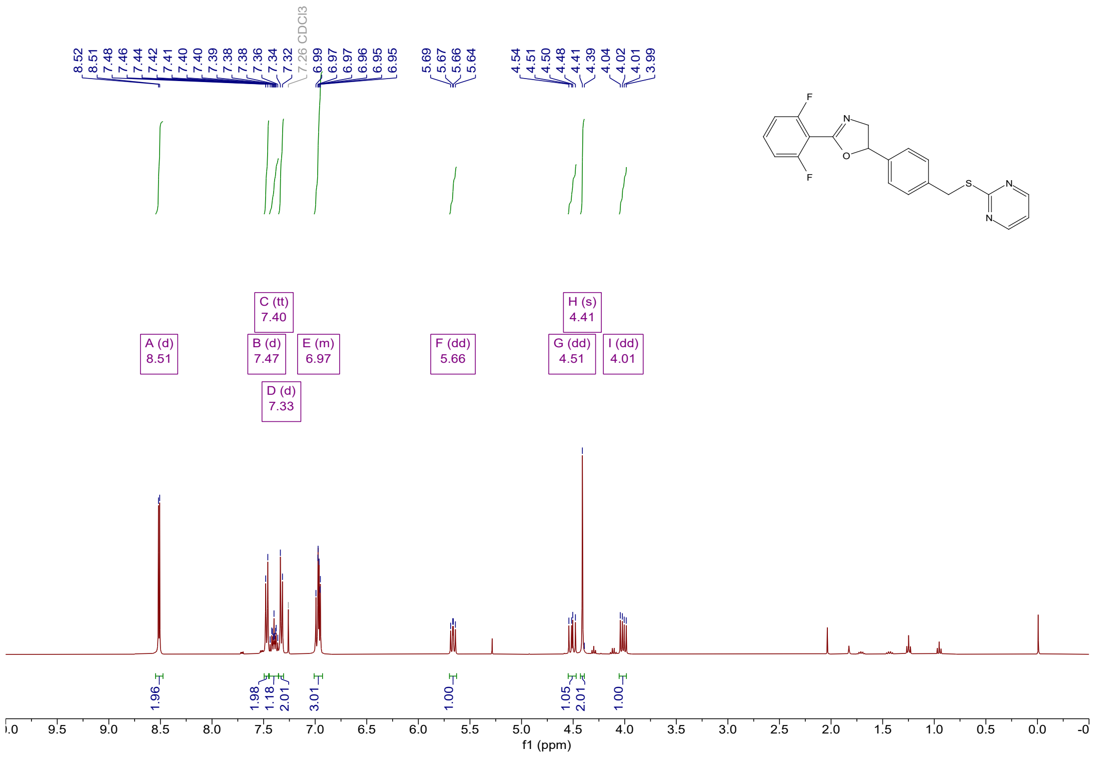

^{1}H NMR spectra of target compound **11w**

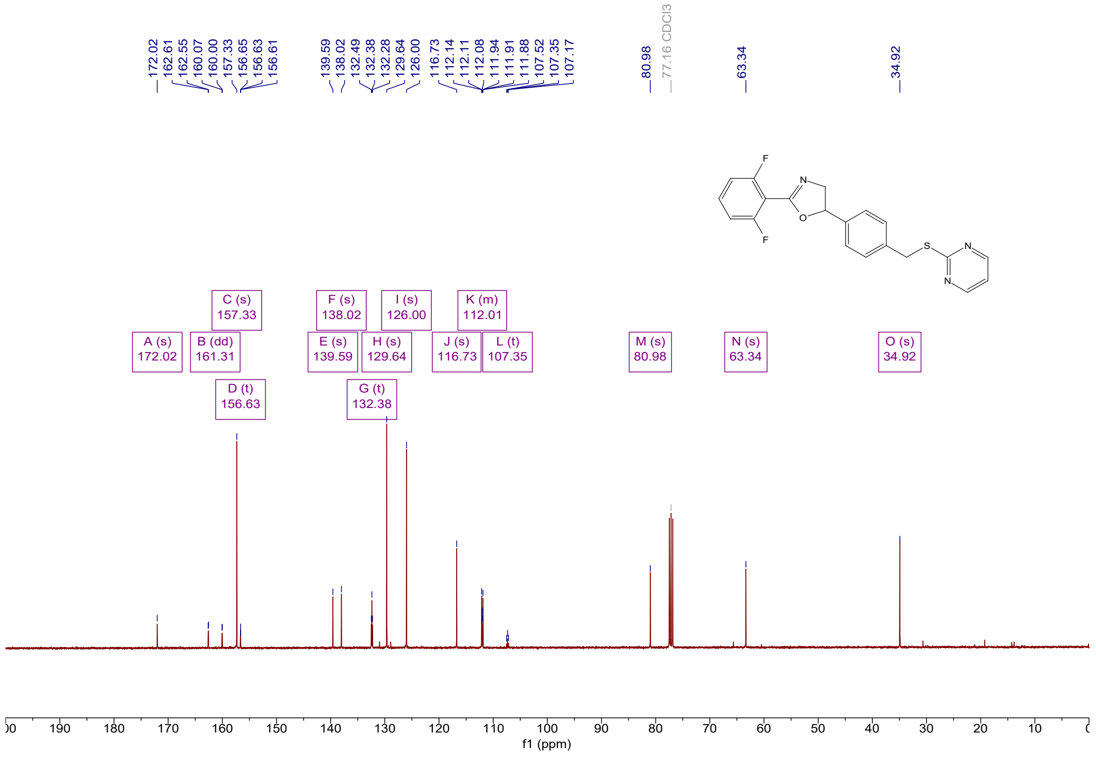

^{13}C NMR spectra of target compound **11w**

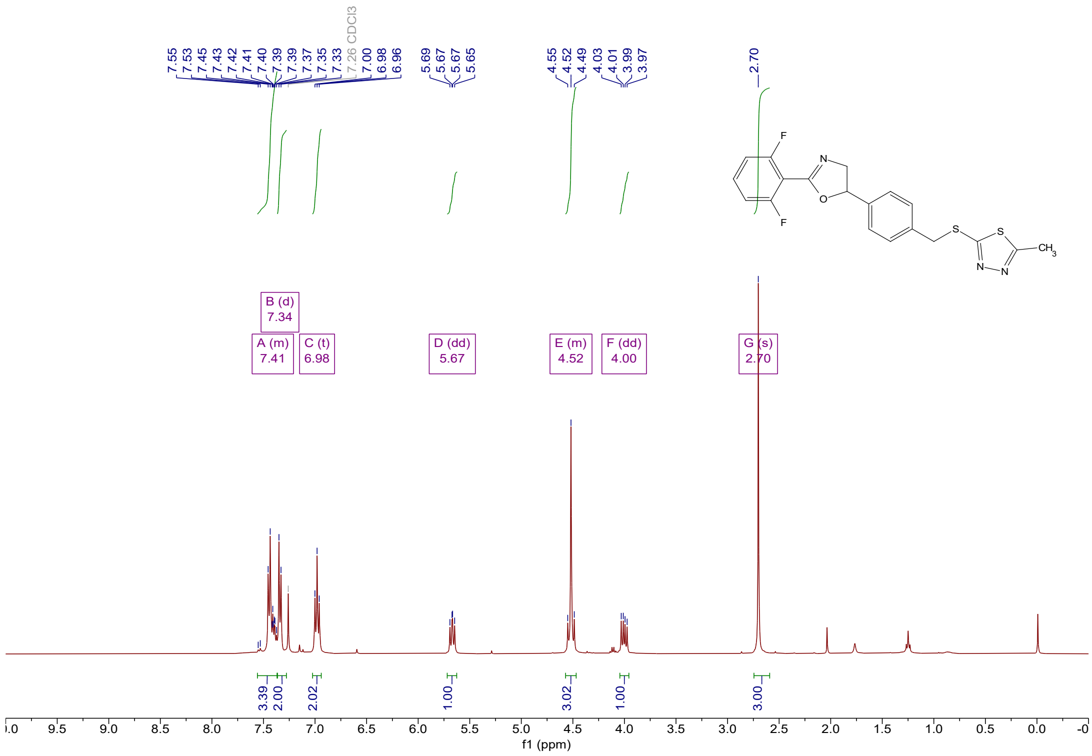

^{1}H NMR spectra of target compound **11x**

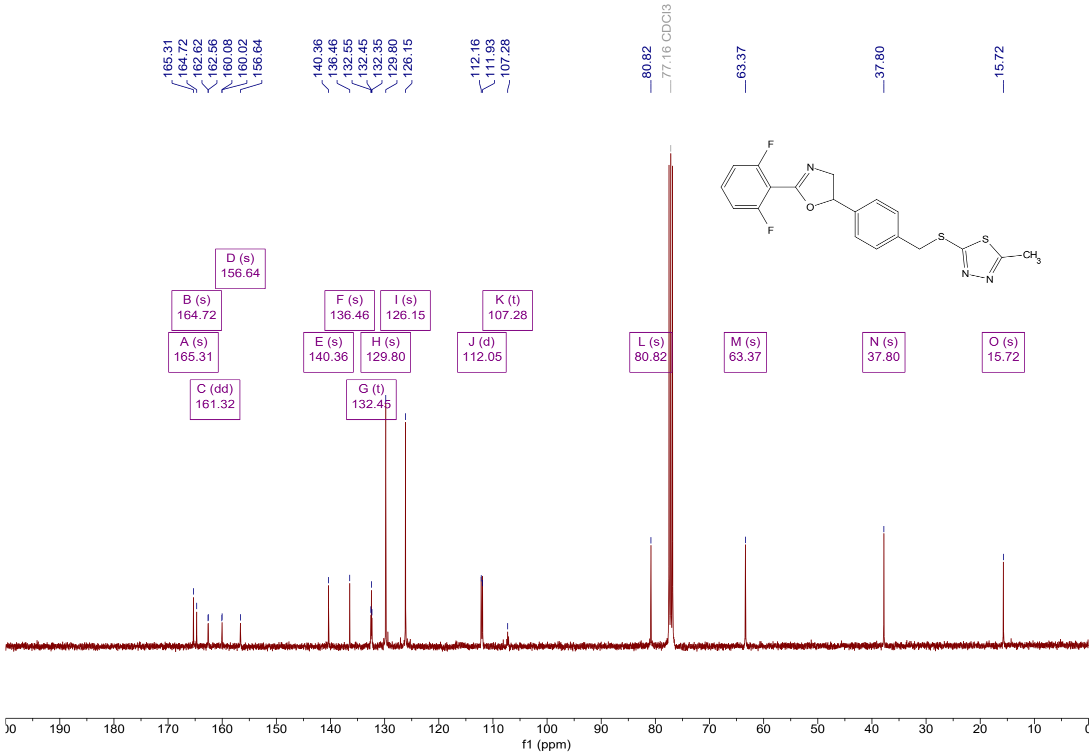

^{13}C NMR spectra of target compound **11x**

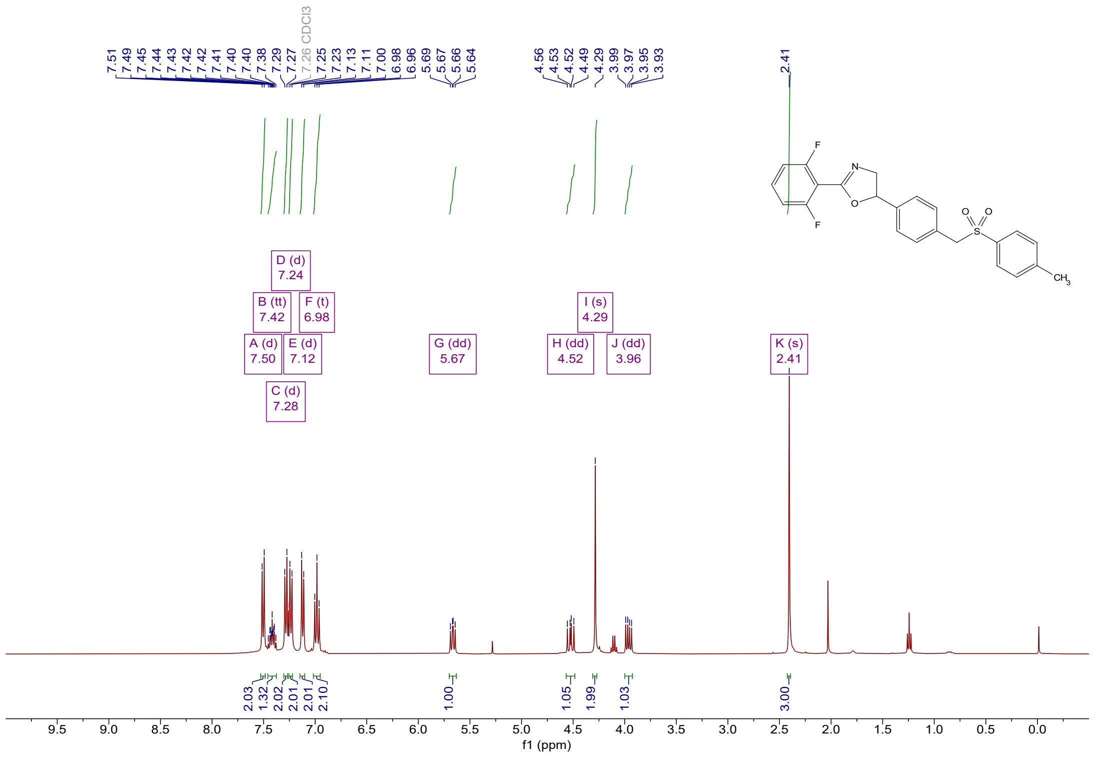

^{1}H NMR spectra of target compound **11y**

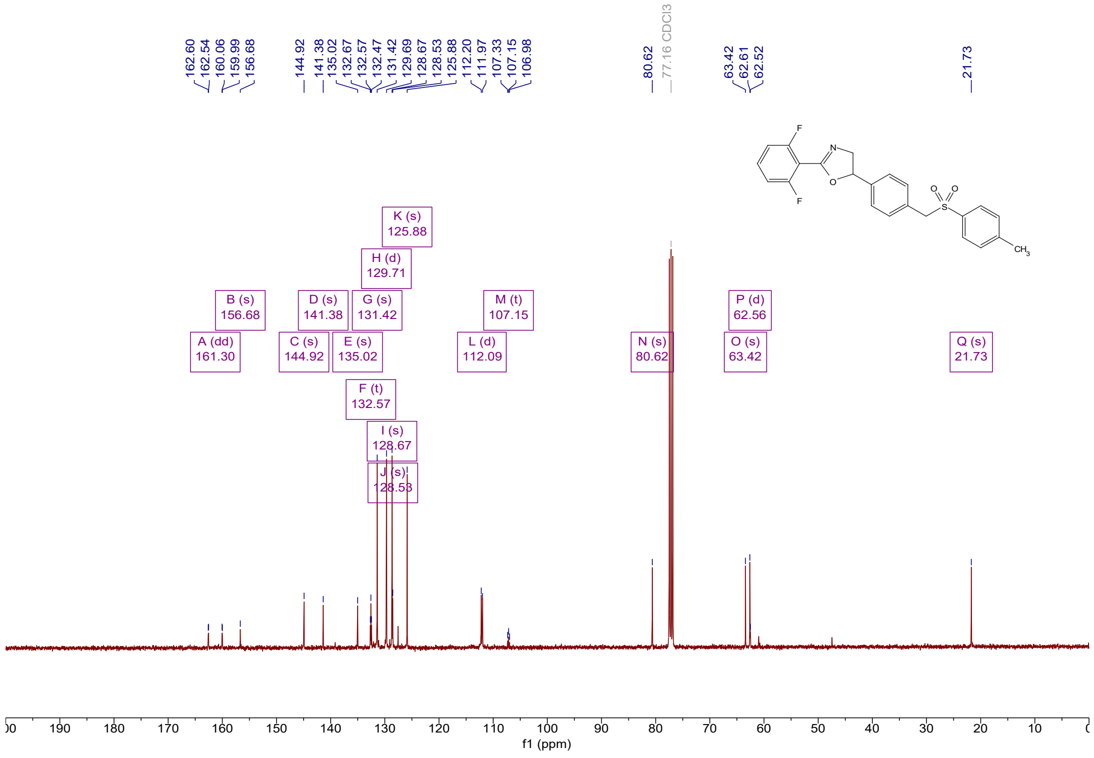

^{13}C NMR spectra of target compound **11y**

5. HRMS spectrums of intermediates and target compounds

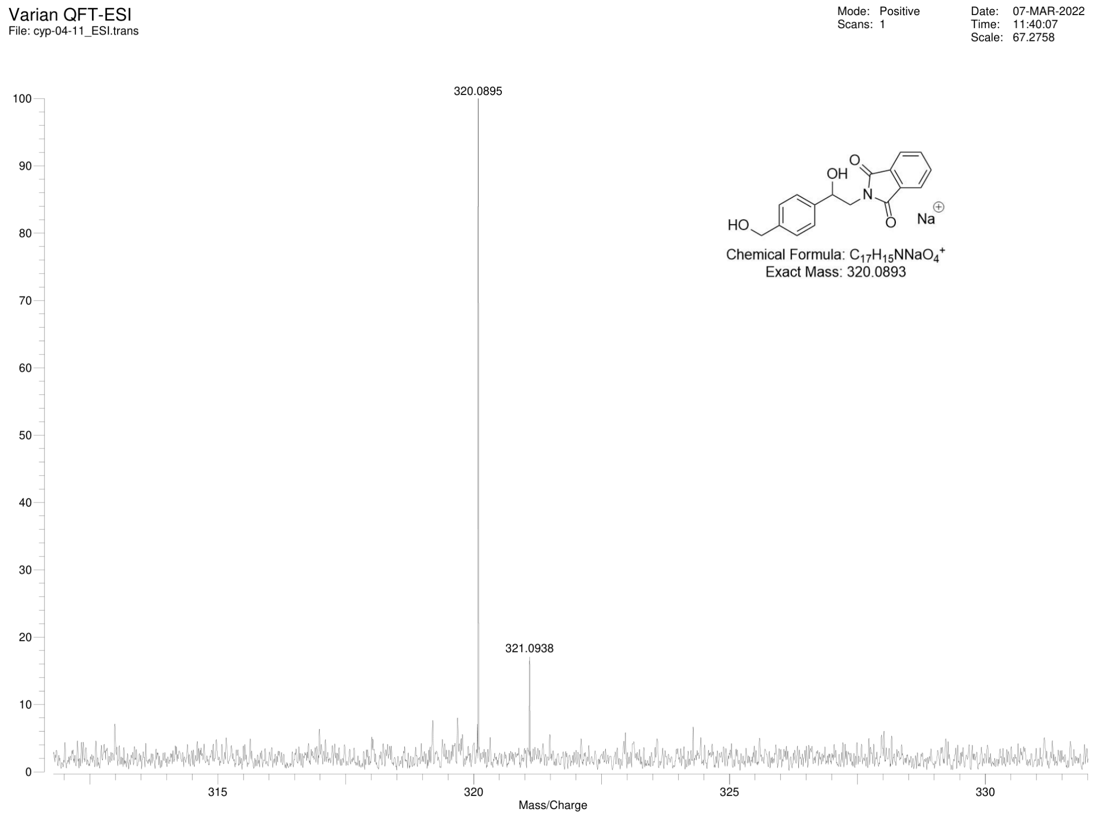

HRMS spectrum of intermediate **4**

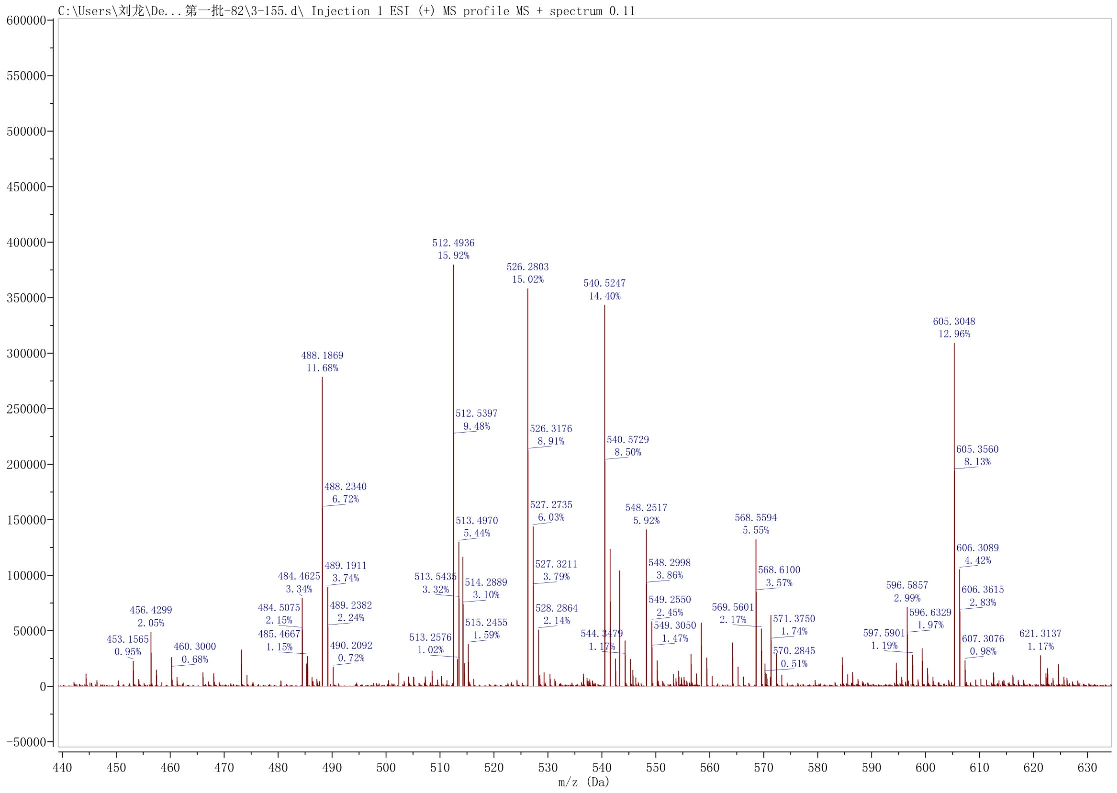

HRMS spectrum of intermediate **5**

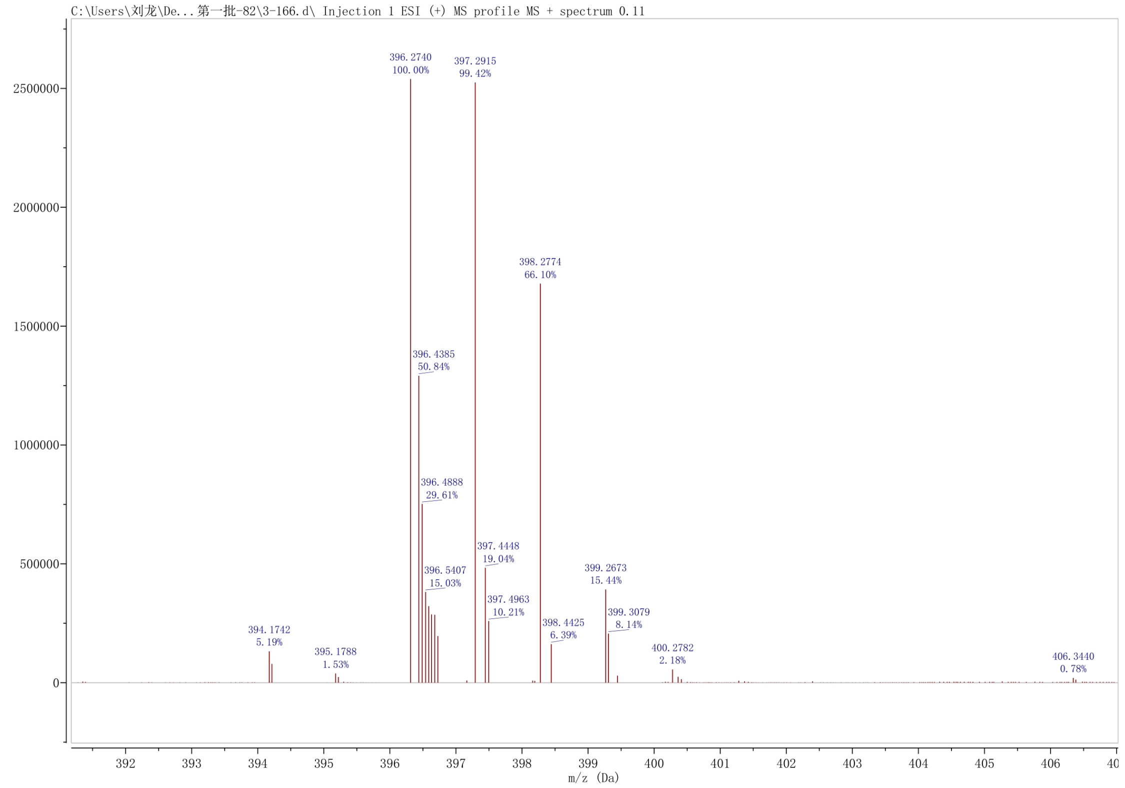

HRMS spectrum of intermediate **6**

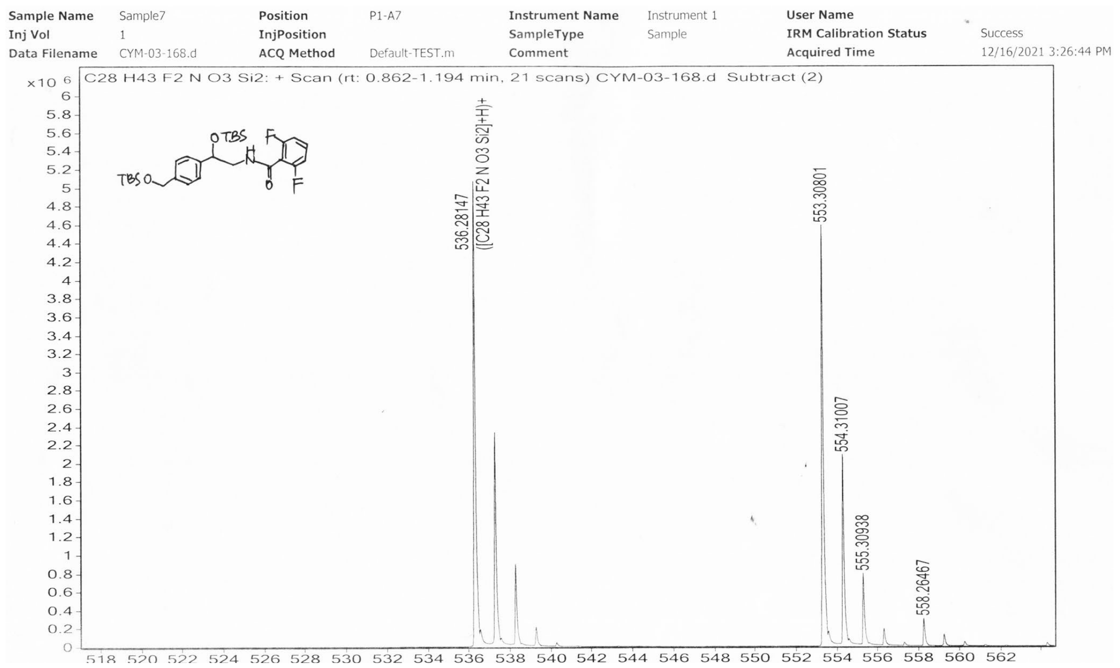

HRMS spectrum of intermediate **7**

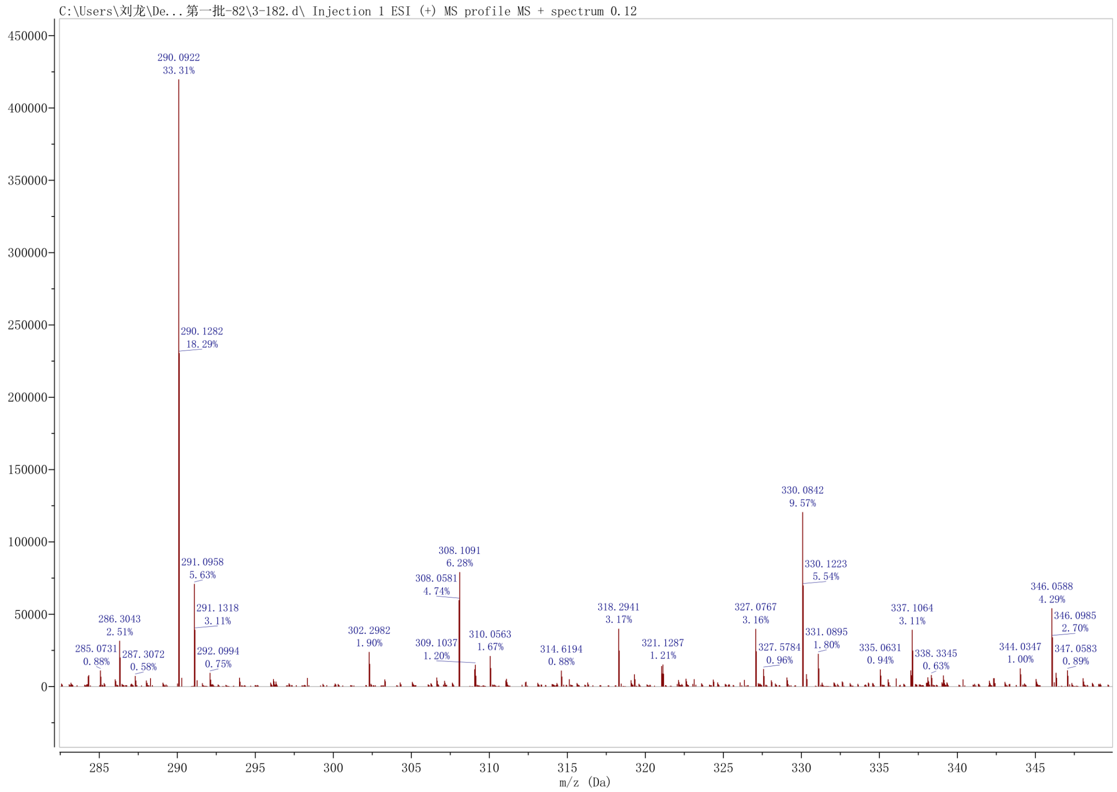

HRMS spectrum of intermediate **8**

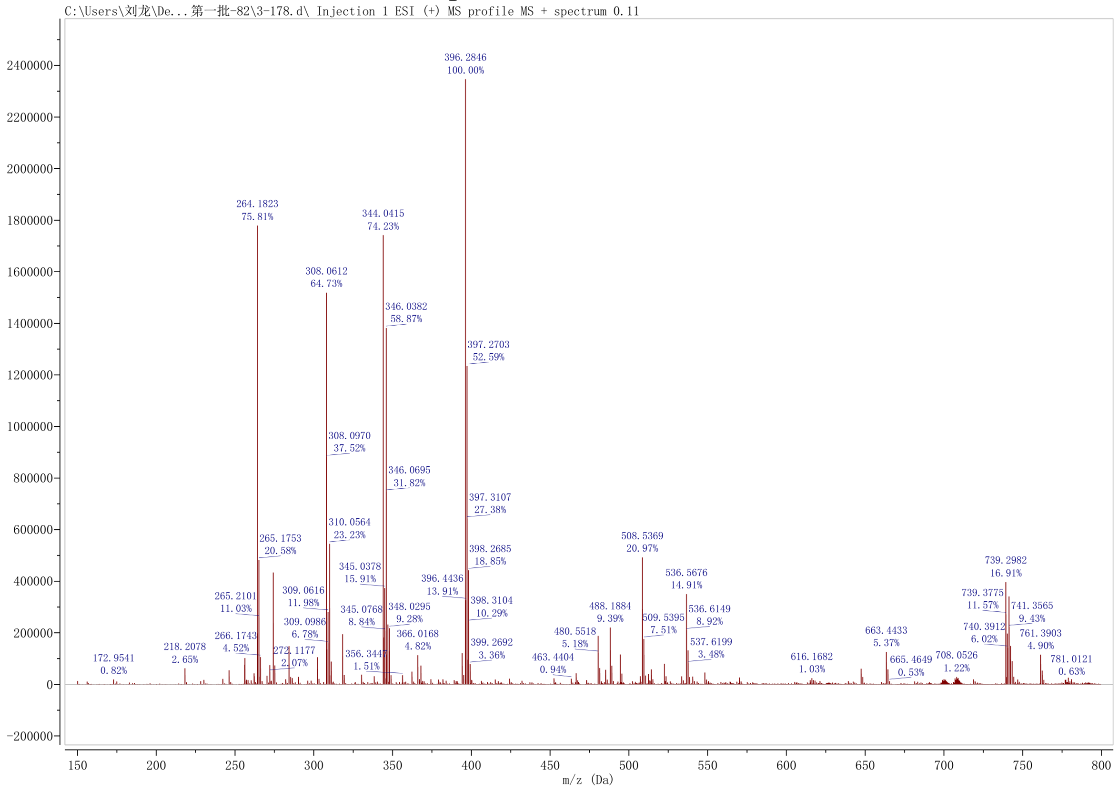

HRMS spectrum of intermediate **9**

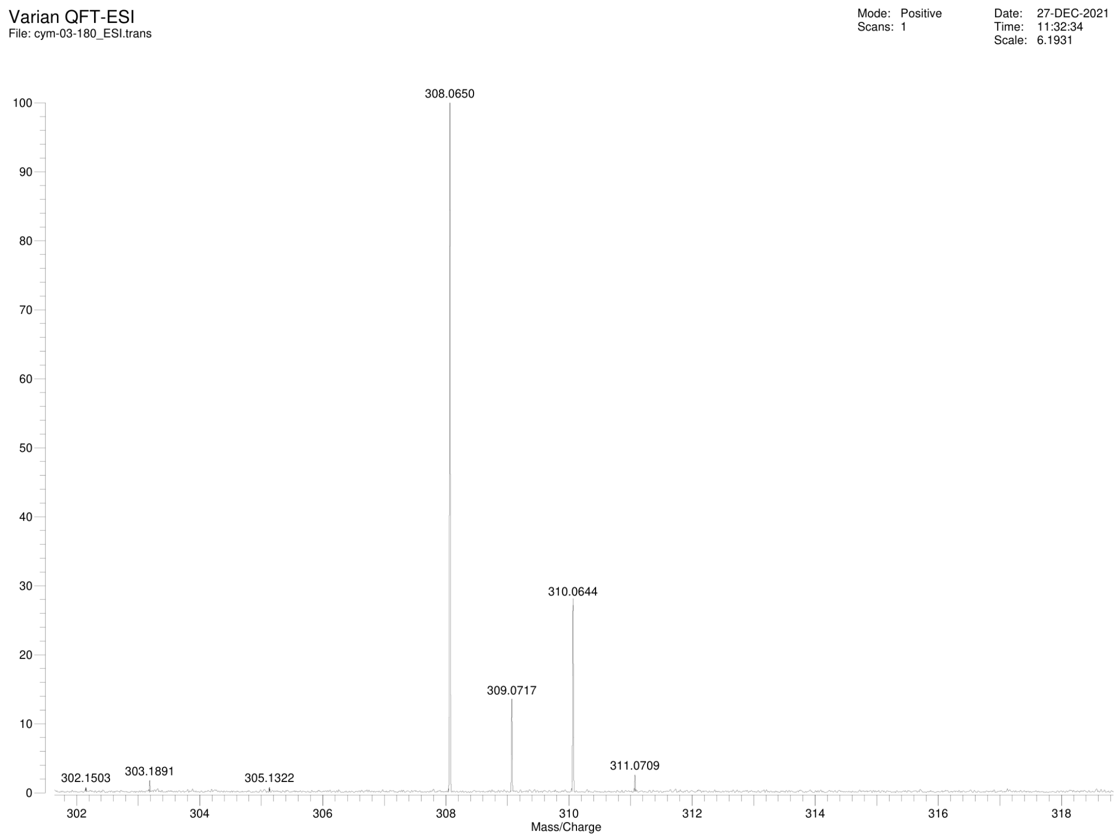

HRMS spectrum of intermediate **10**

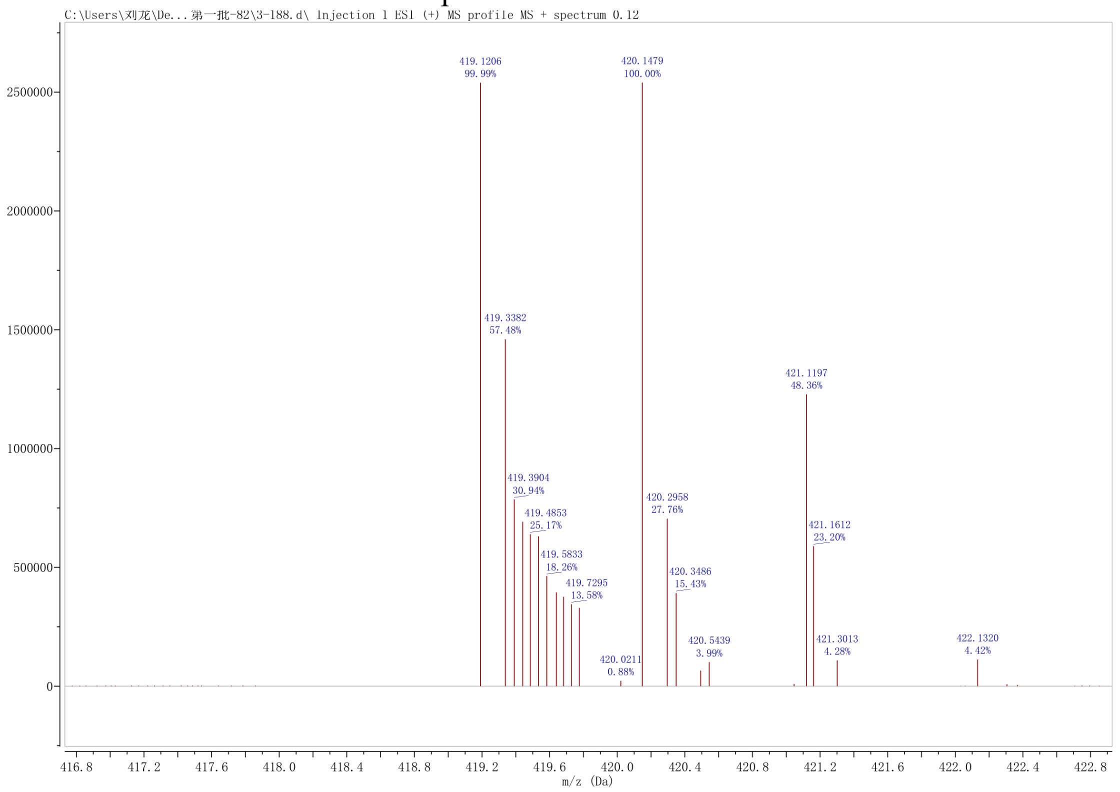

HRMS spectrum of target compound **11a**

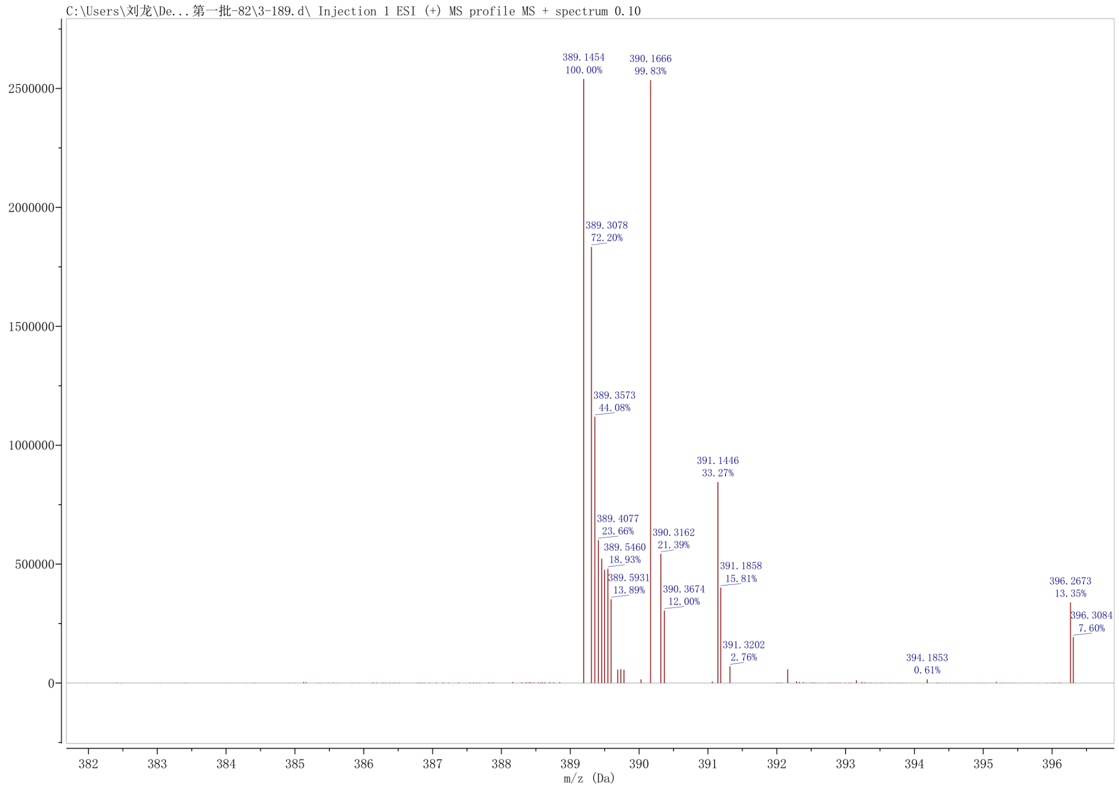

HRMS spectrum of target compound **11b**

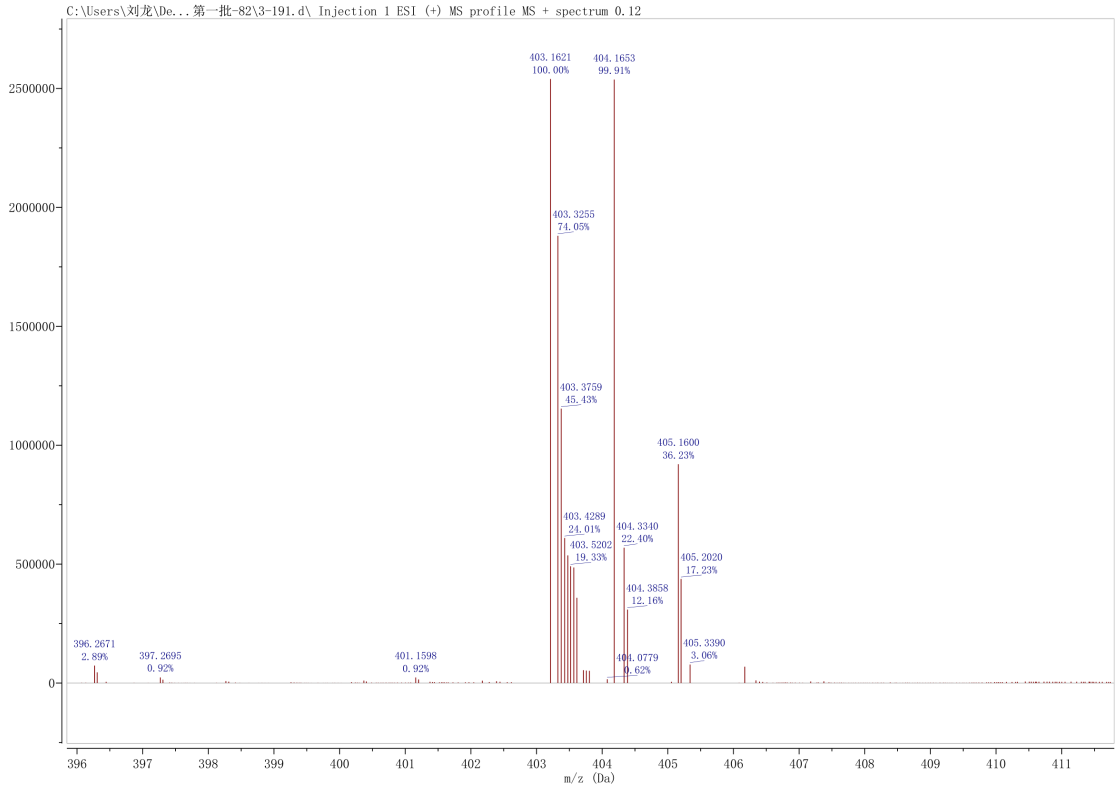

HRMS spectrum of target compound **11c**

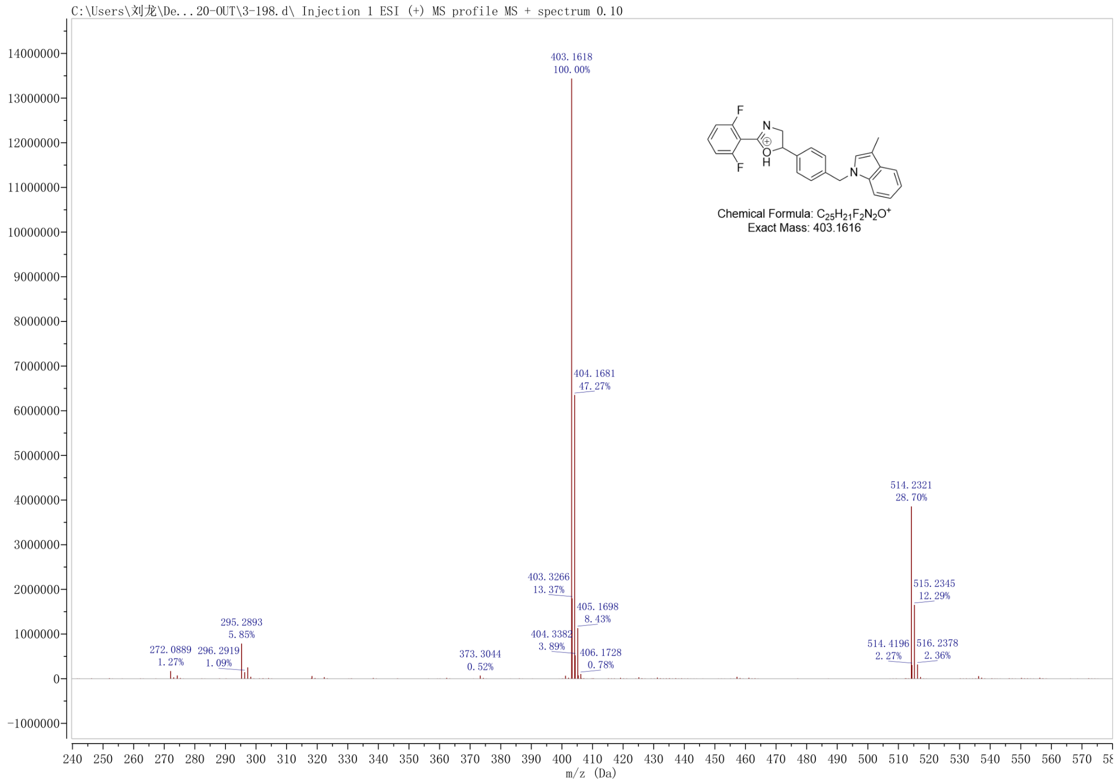

HRMS spectrum of target compound **11d**

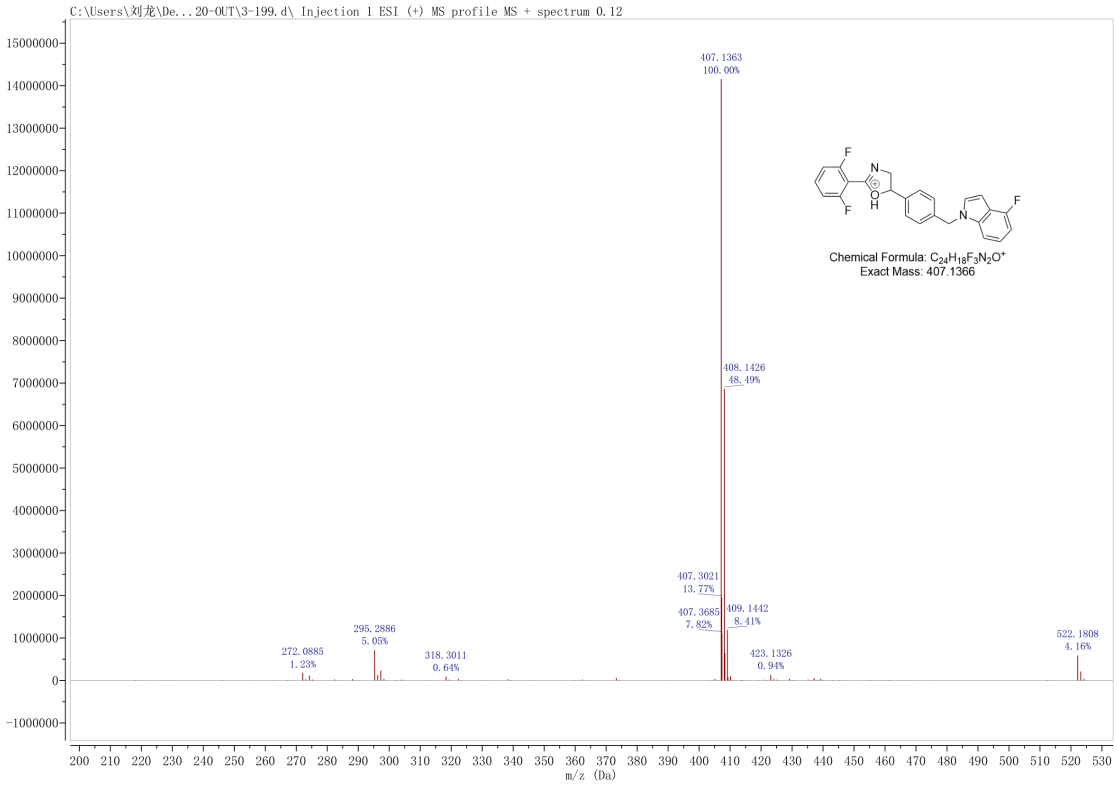

HRMS spectrum of target compound **11e**

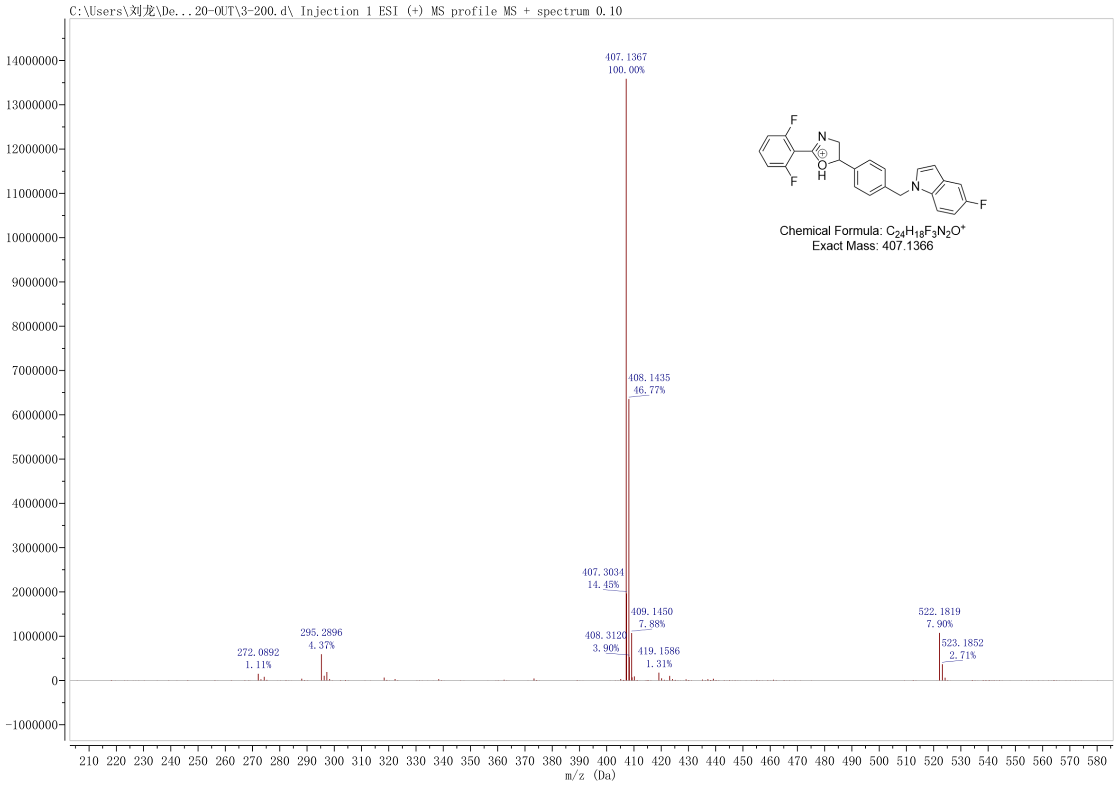

HRMS spectrum of target compound **11f**

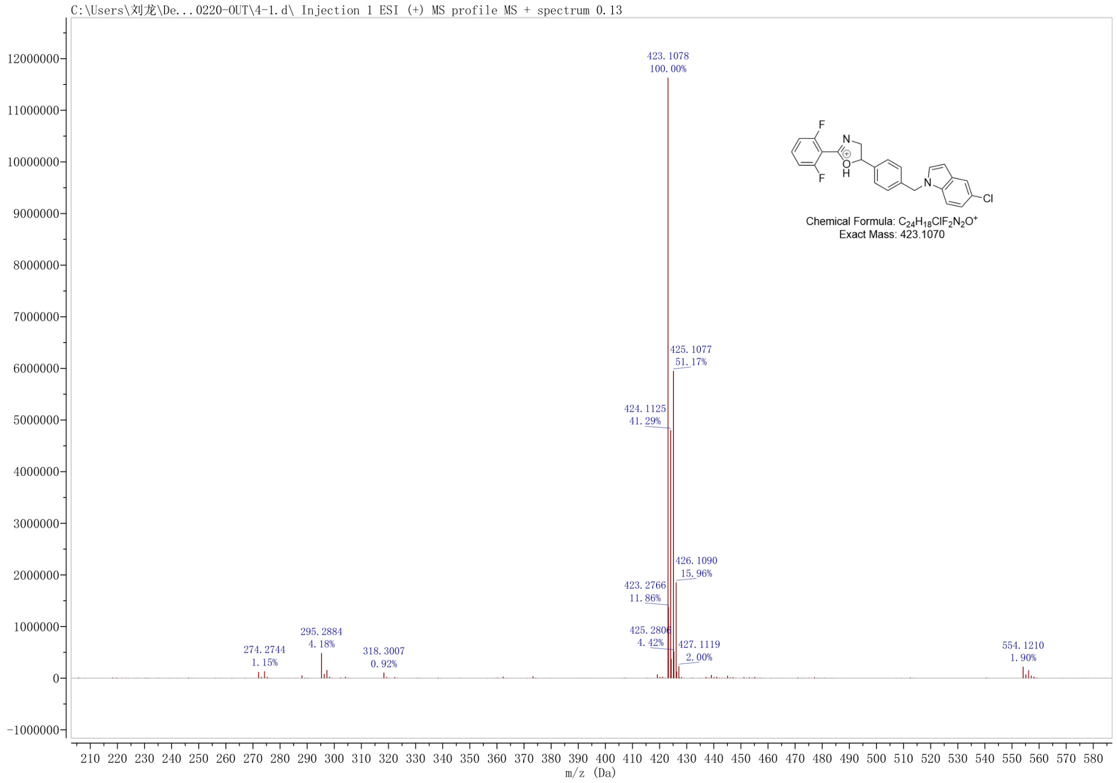

HRMS spectrum of target compound **11g**

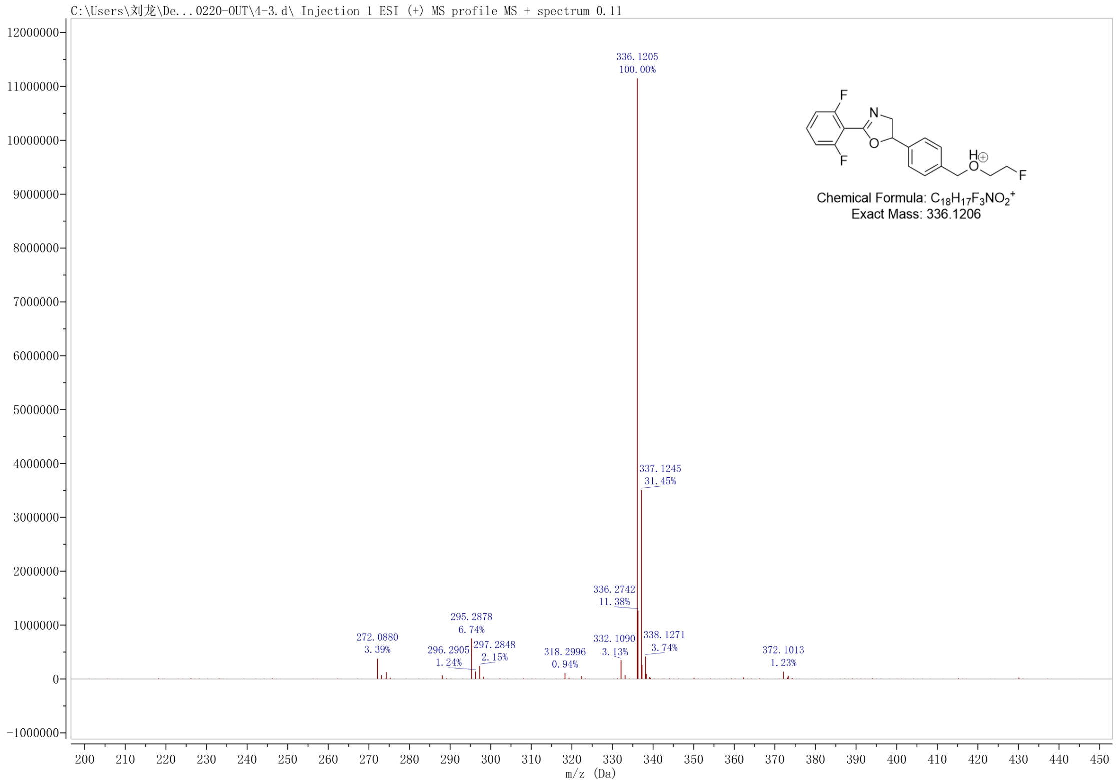

HRMS spectrum of target compound **11h**

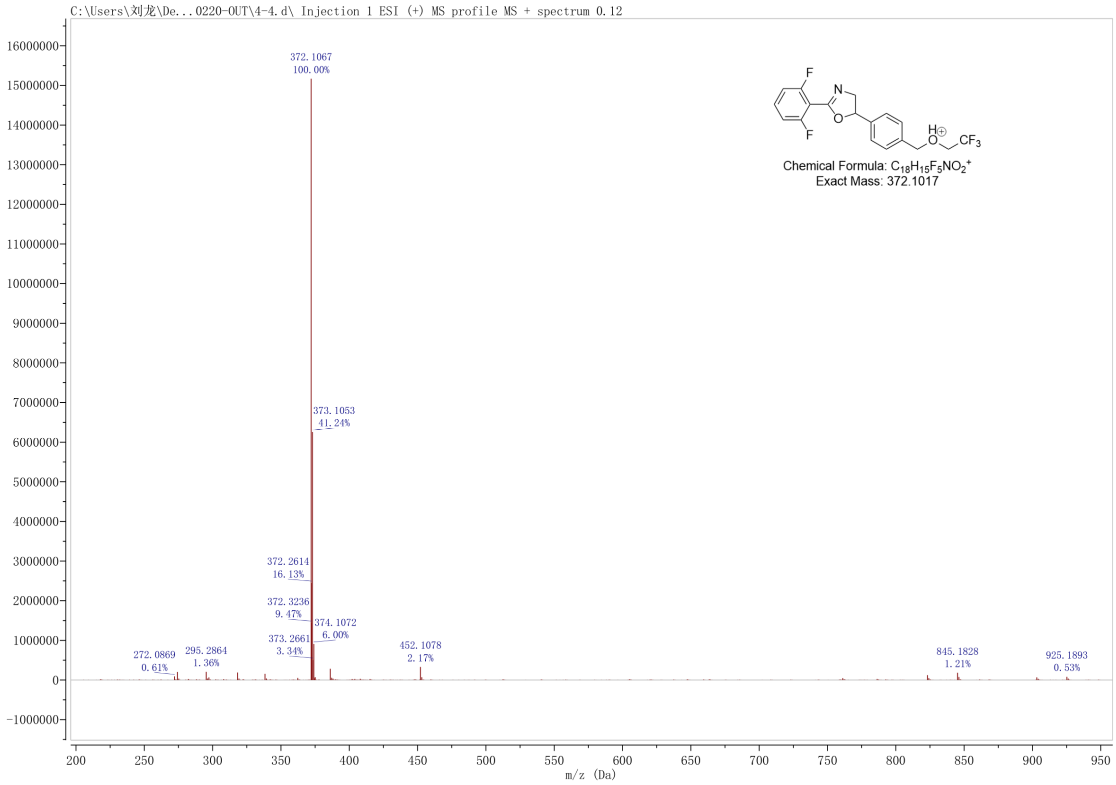

HRMS spectrum of target compound **11i**

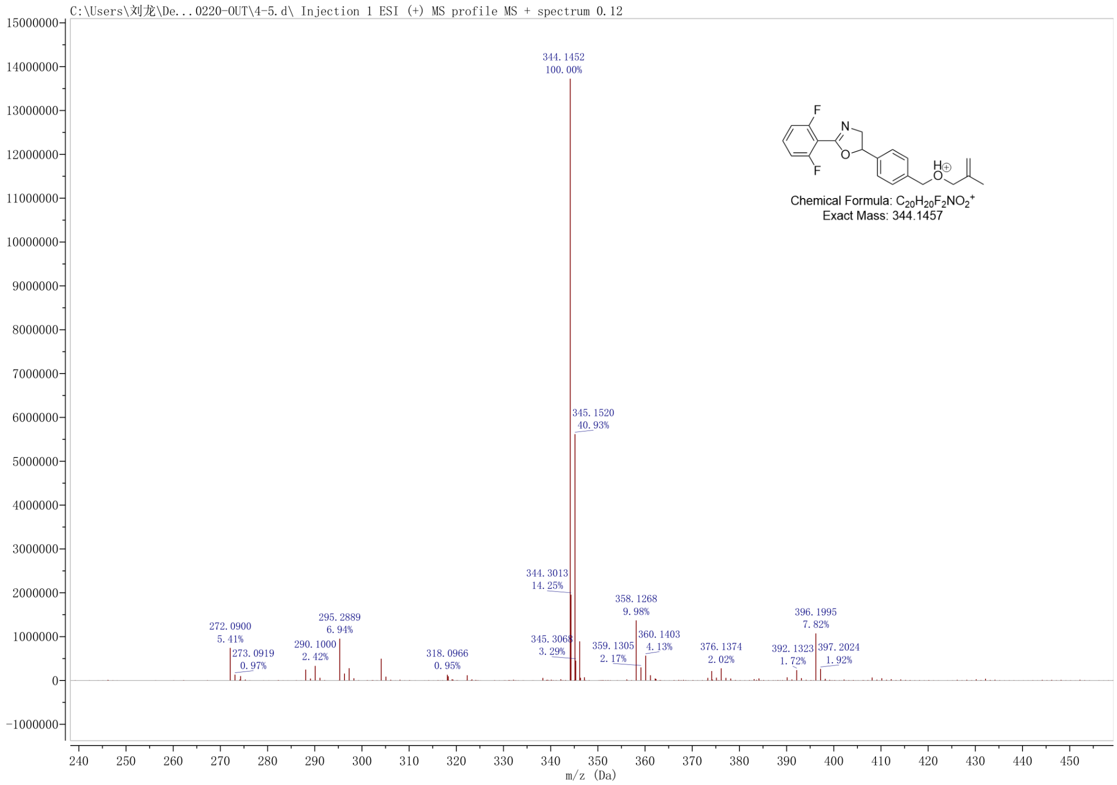

HRMS spectrum of target compound **11j**

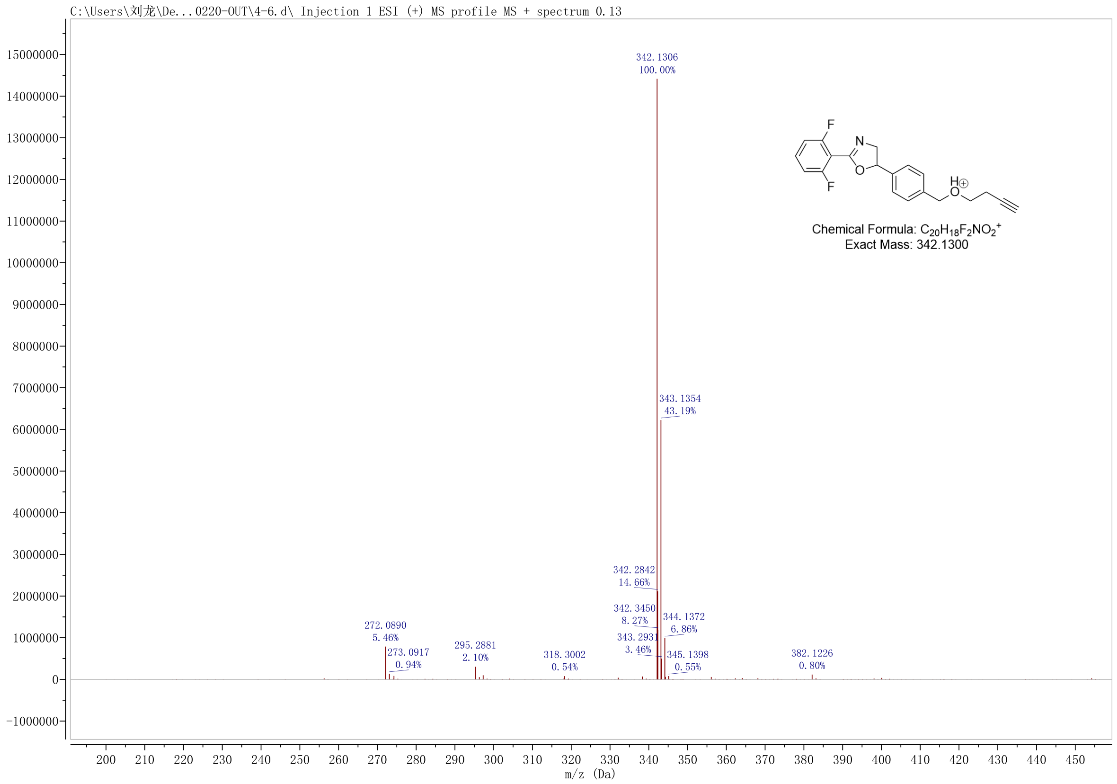

HRMS spectrum of target compound **11k**

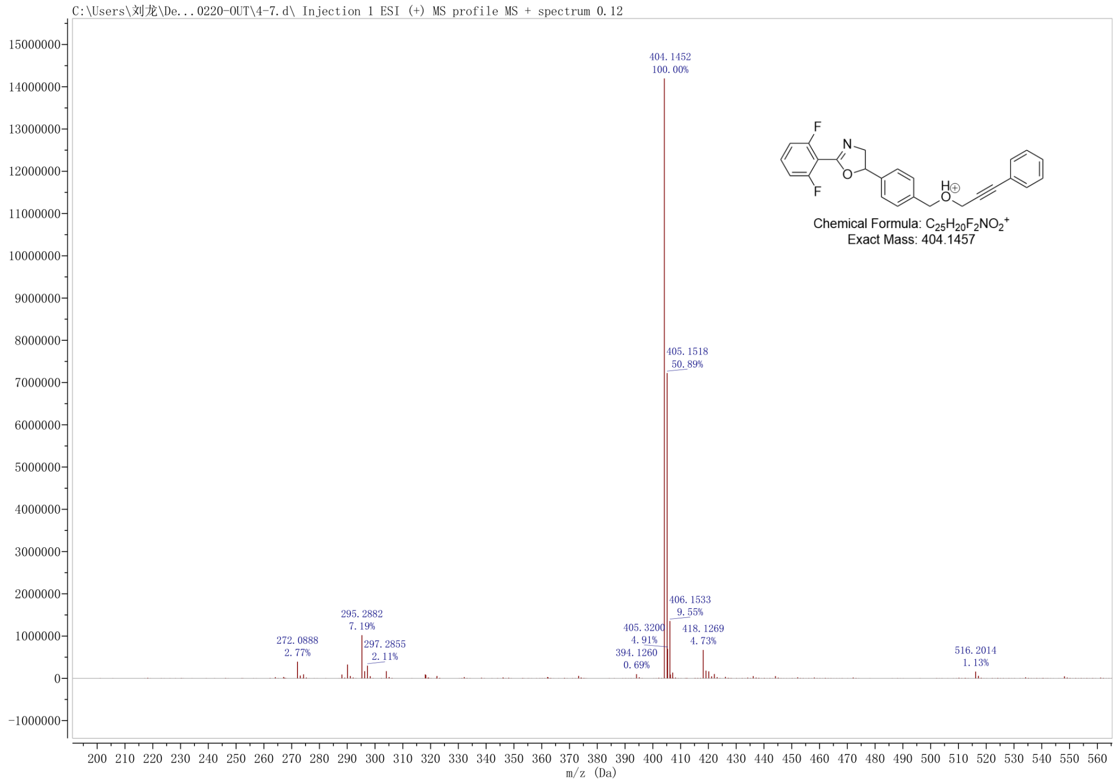

HRMS spectrum of target compound **11l**

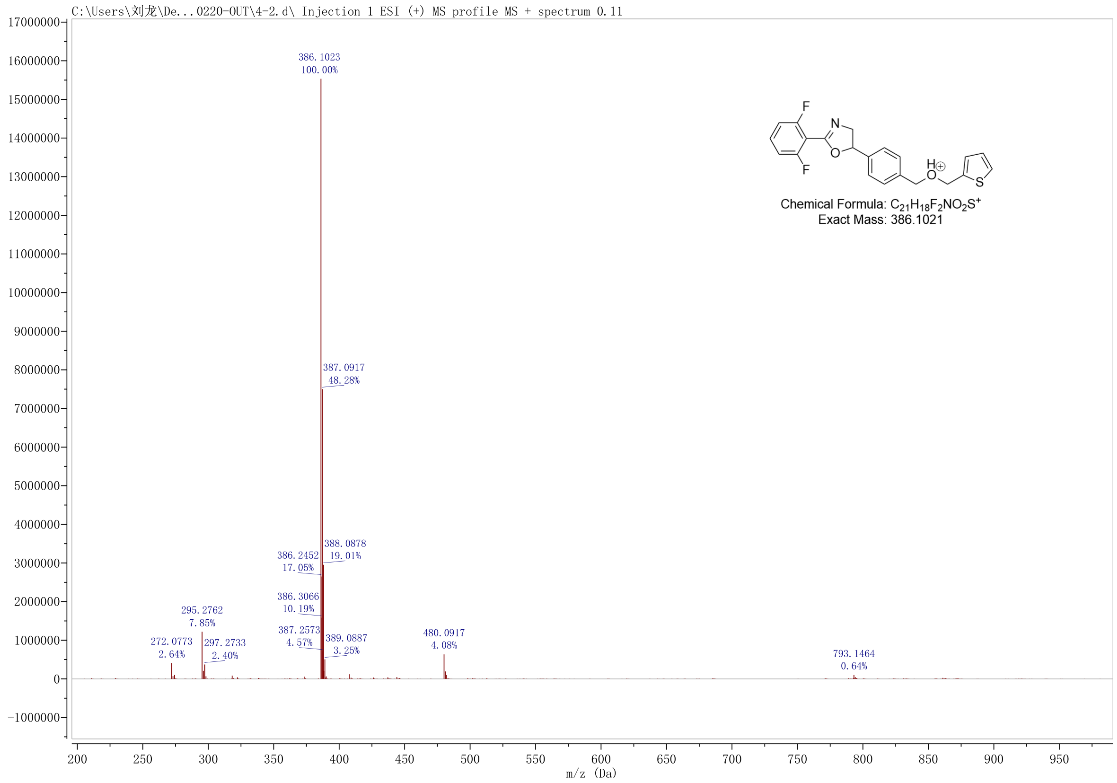

HRMS spectrum of target compound **11m**

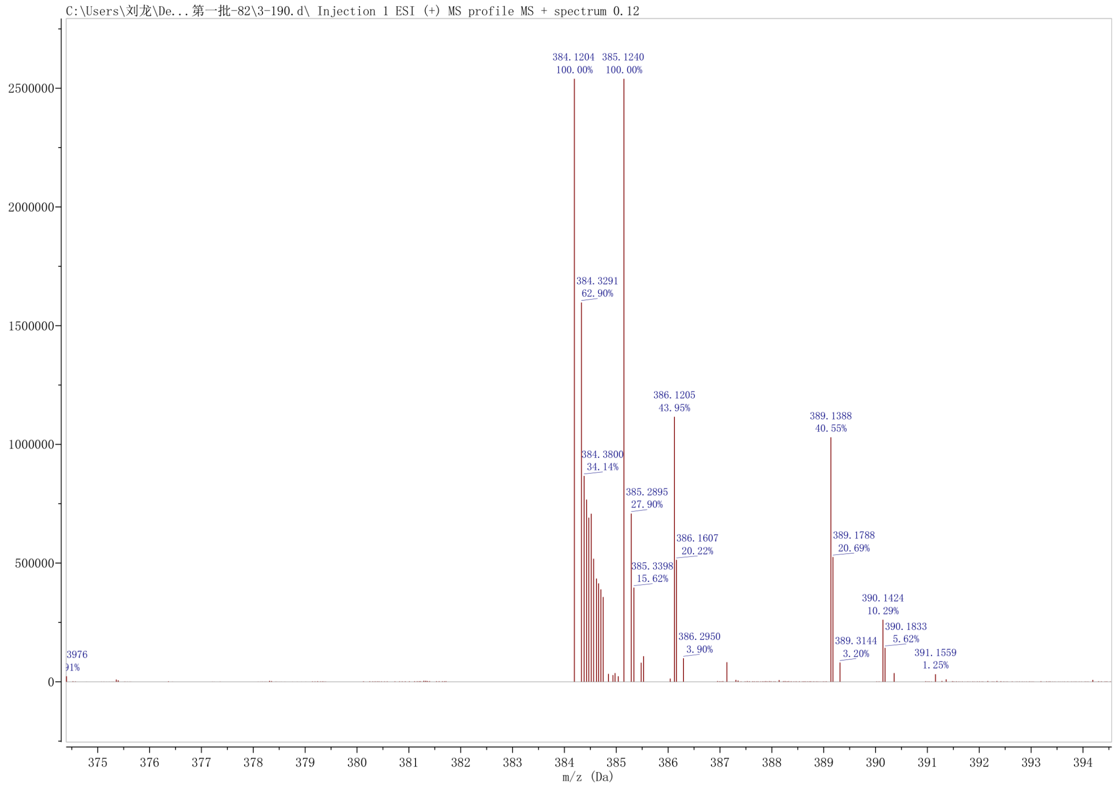

HRMS spectrum of target compound **11n**

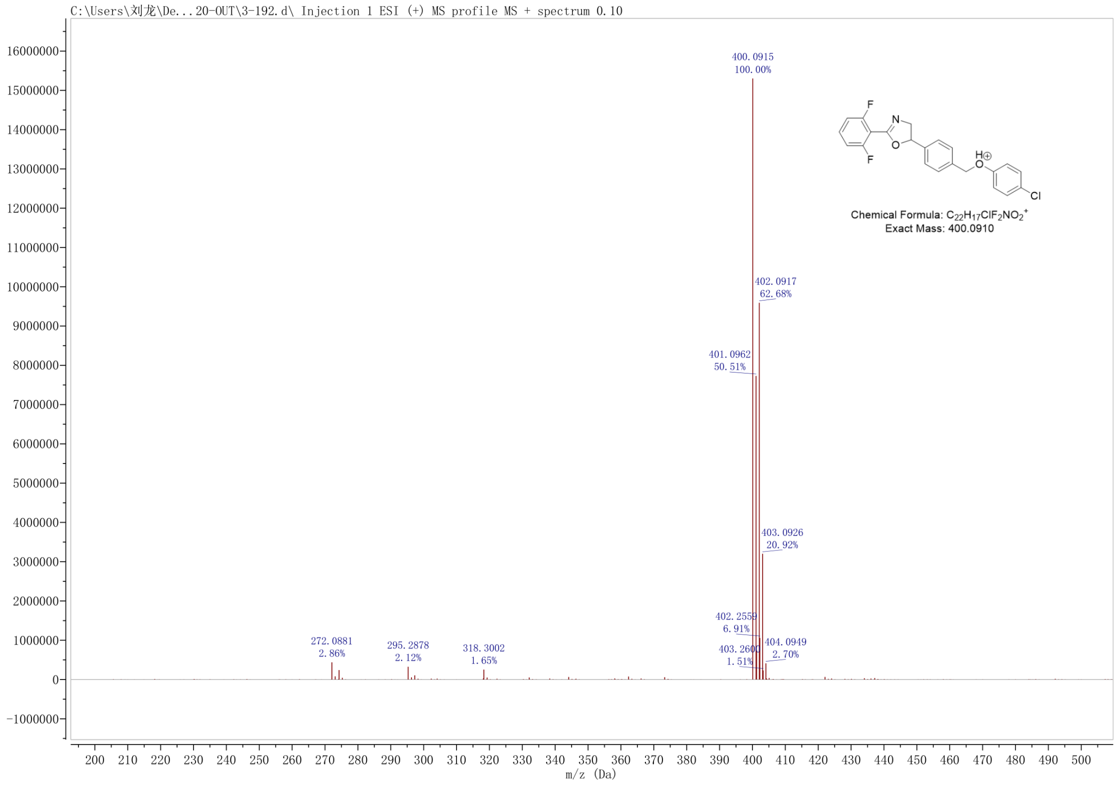

HRMS spectrum of target compound **11o**

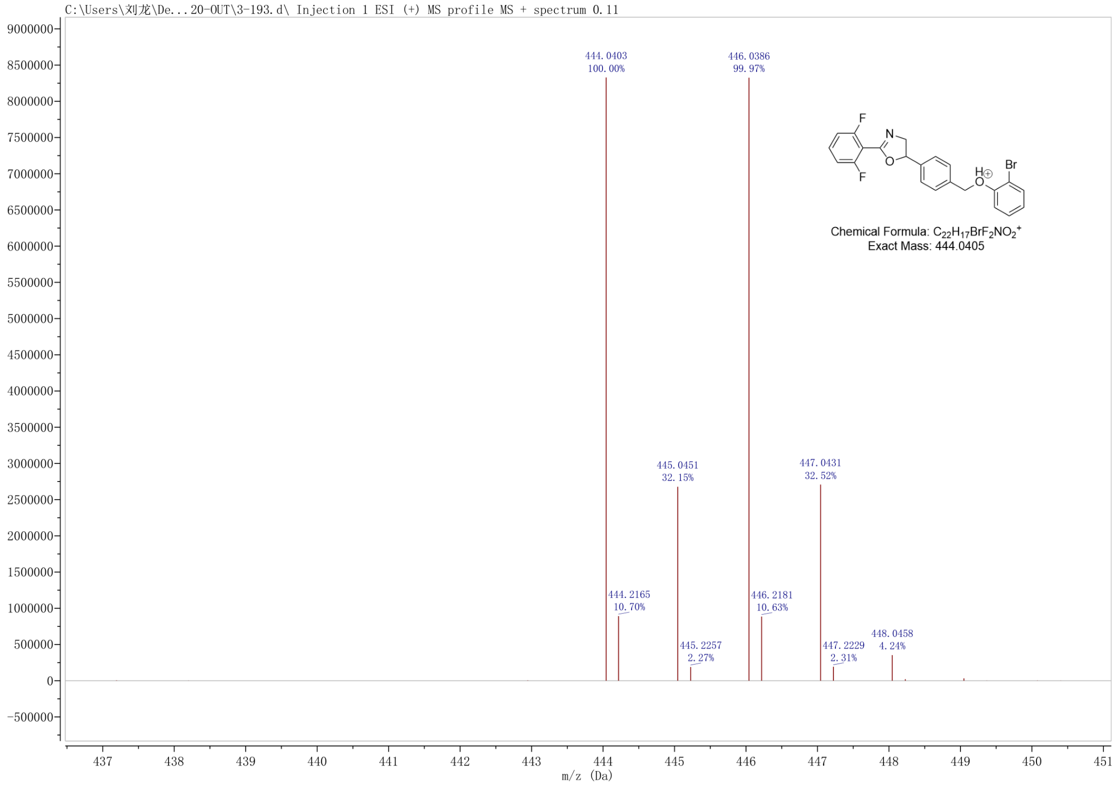

HRMS spectrum of target compound **11p**

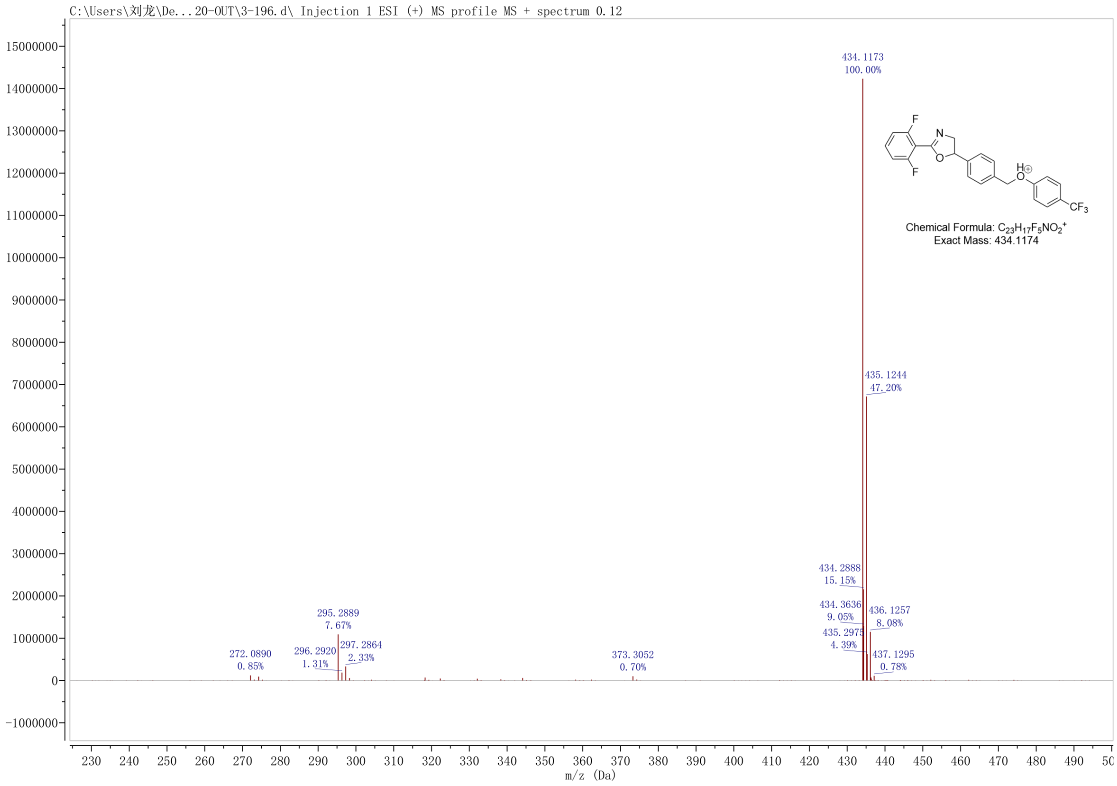

HRMS spectrum of target compound **11q**

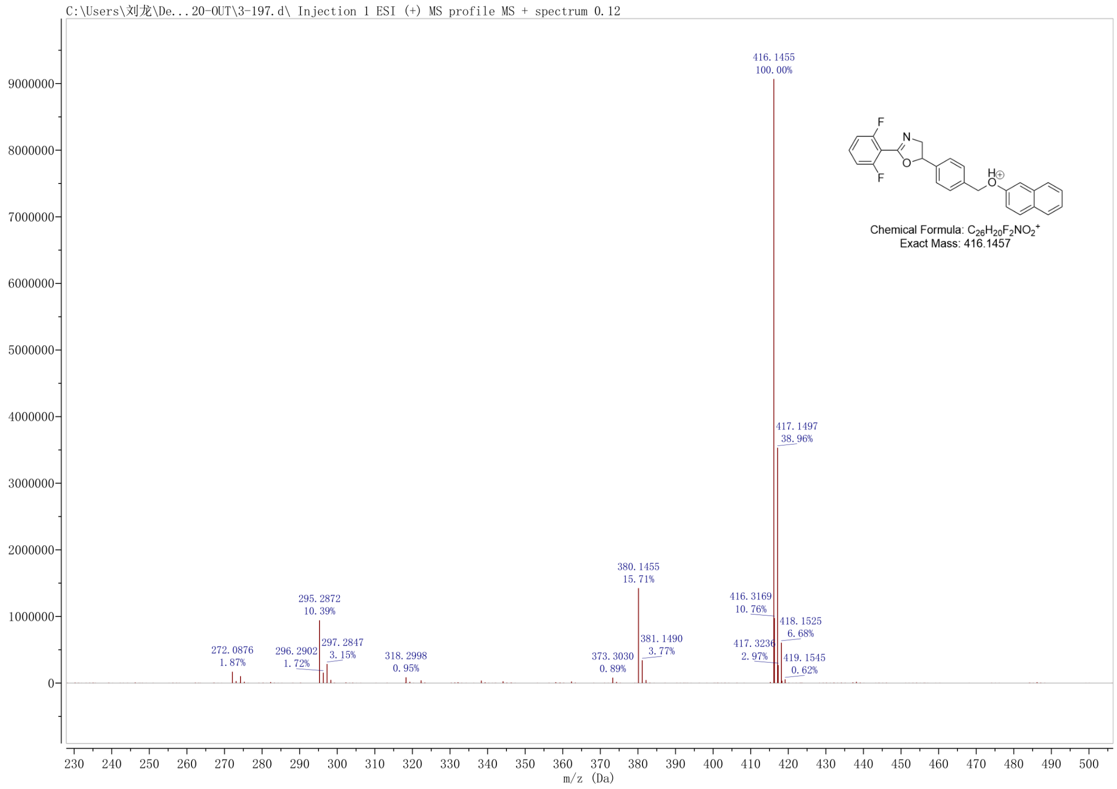

HRMS spectrum of target compound **11r**

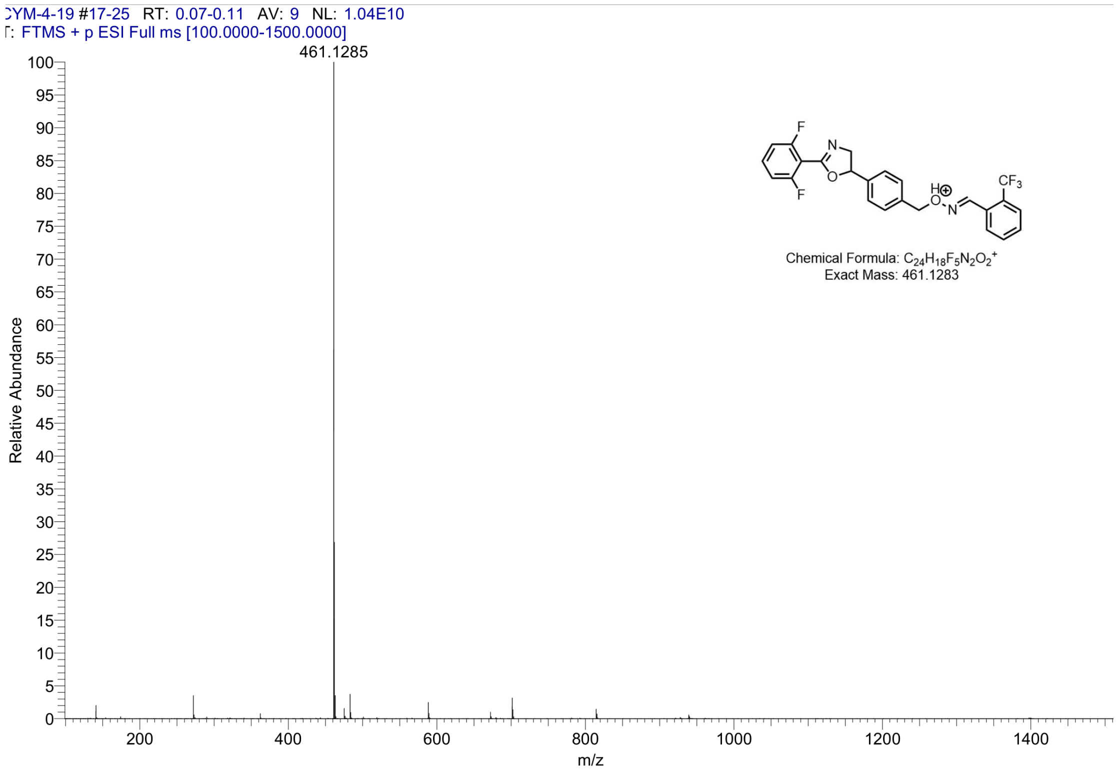

HRMS spectrum of target compound **11s**

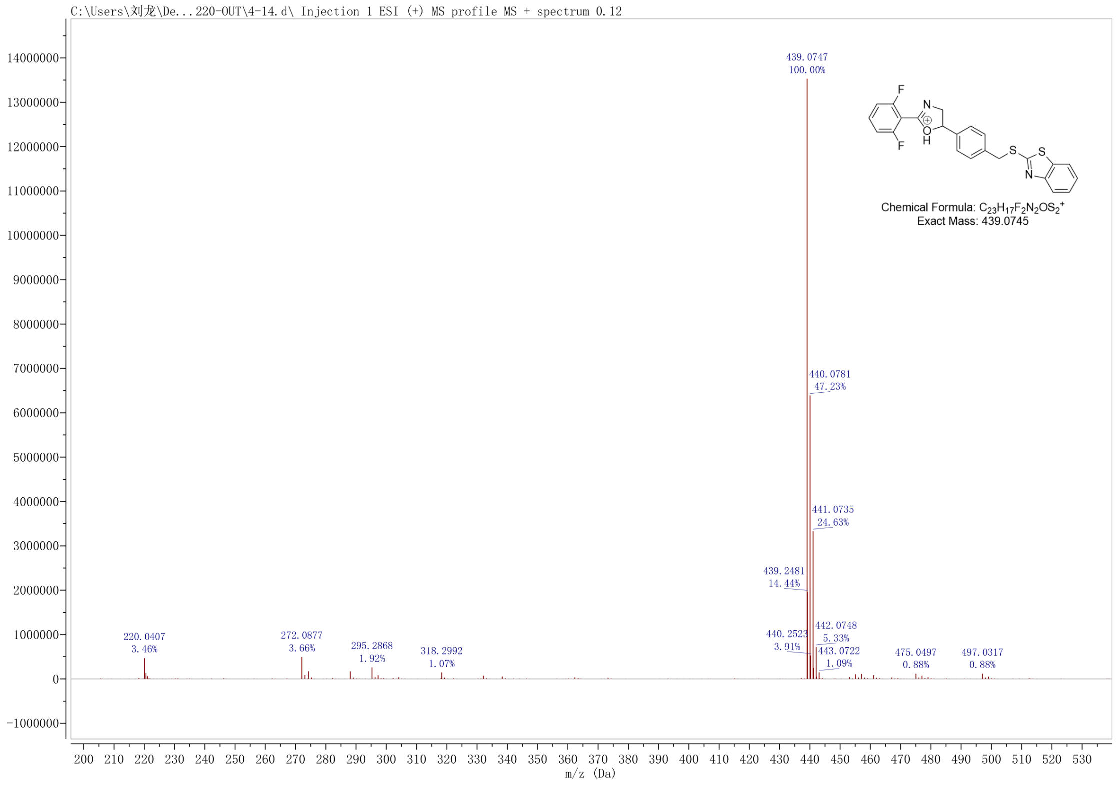

HRMS spectrum of target compound **11t**

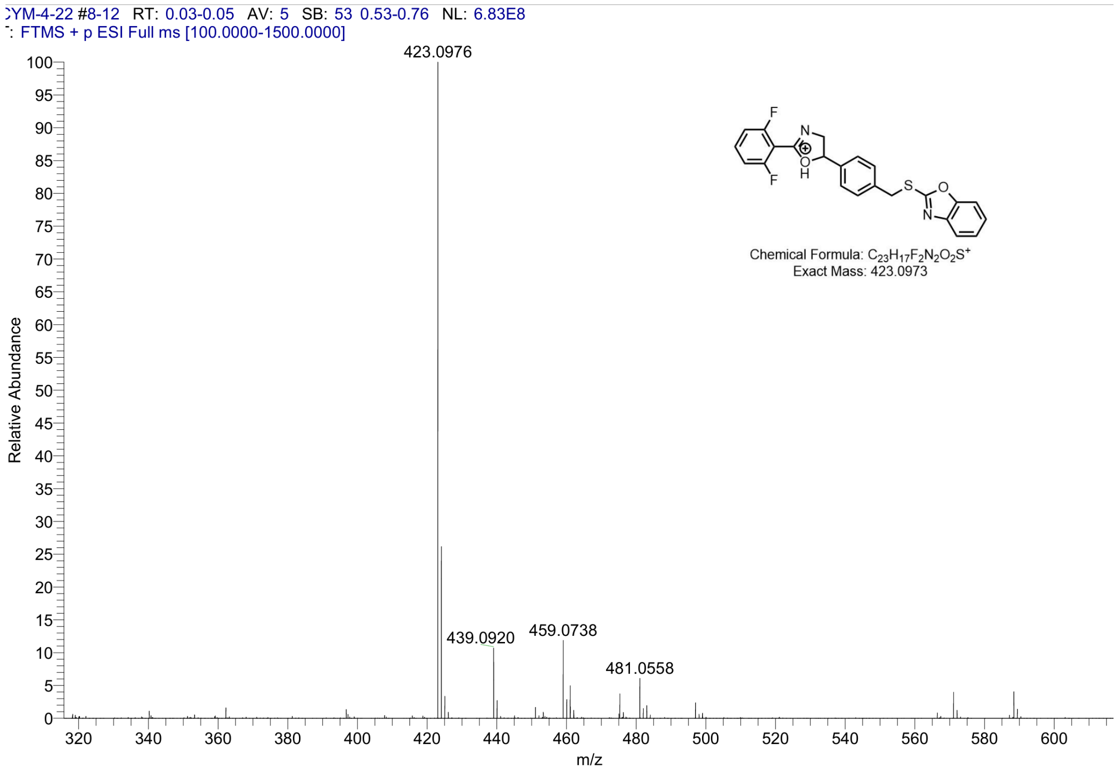

HRMS spectrum of target compound **11u**

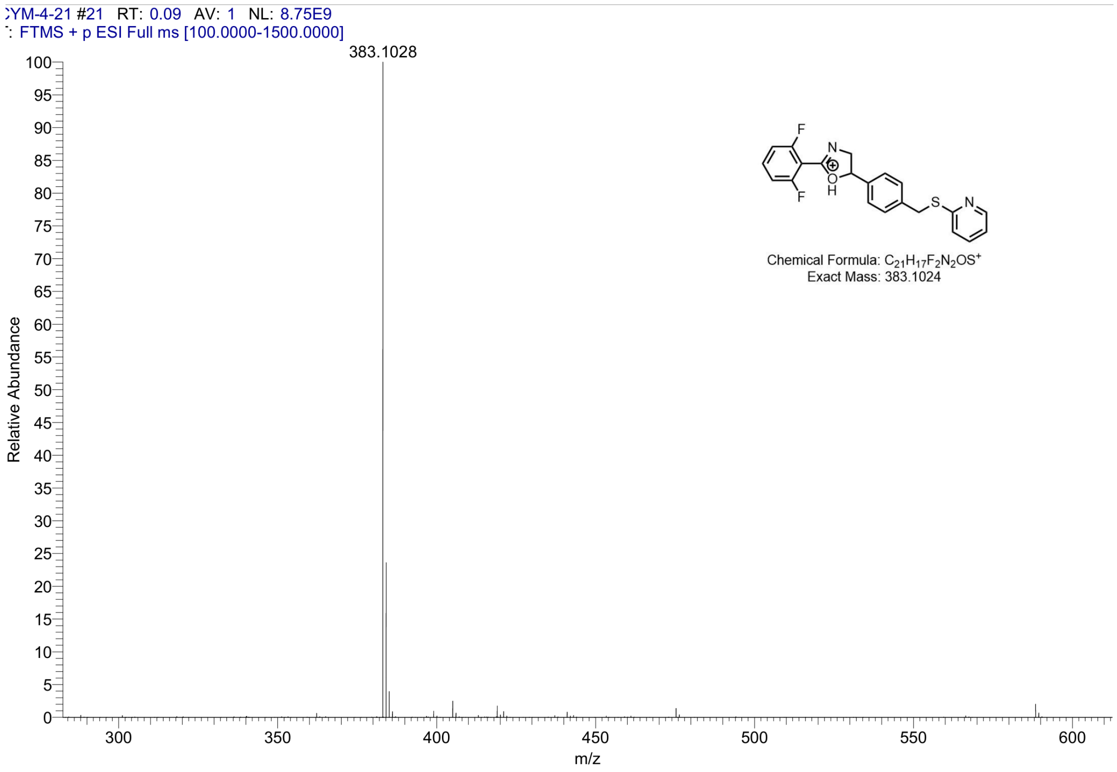

HRMS spectrum of target compound **11v**

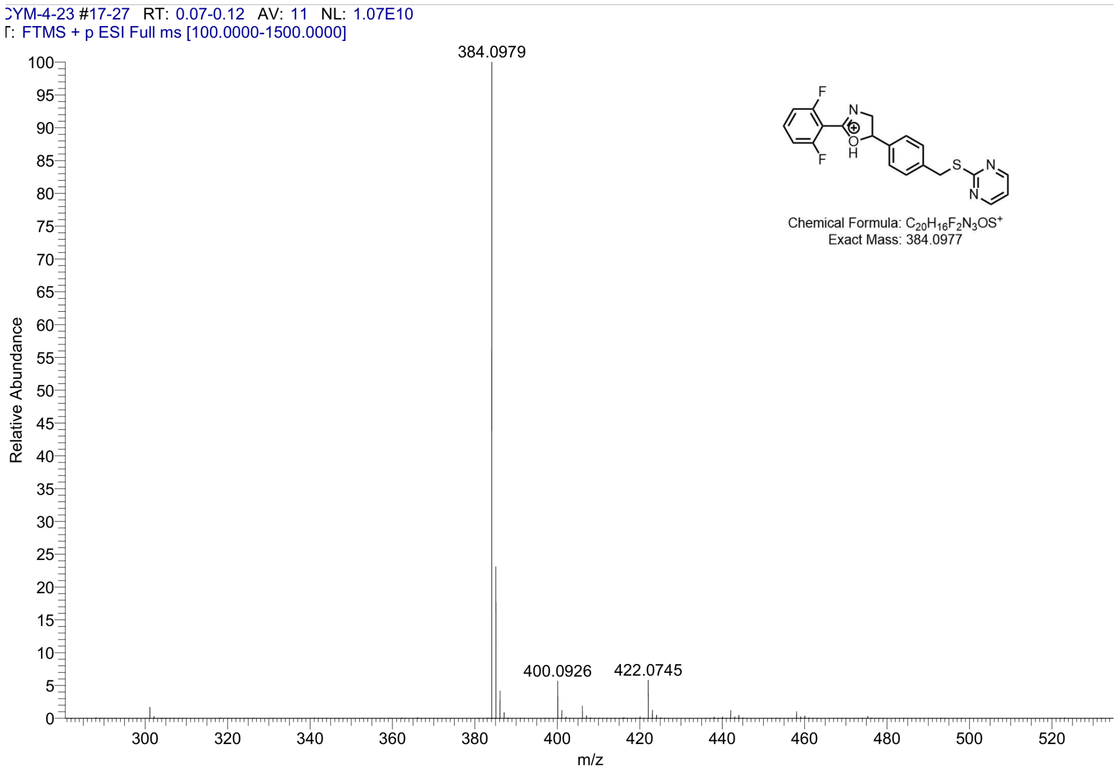

HRMS spectrum of target compound **11w**

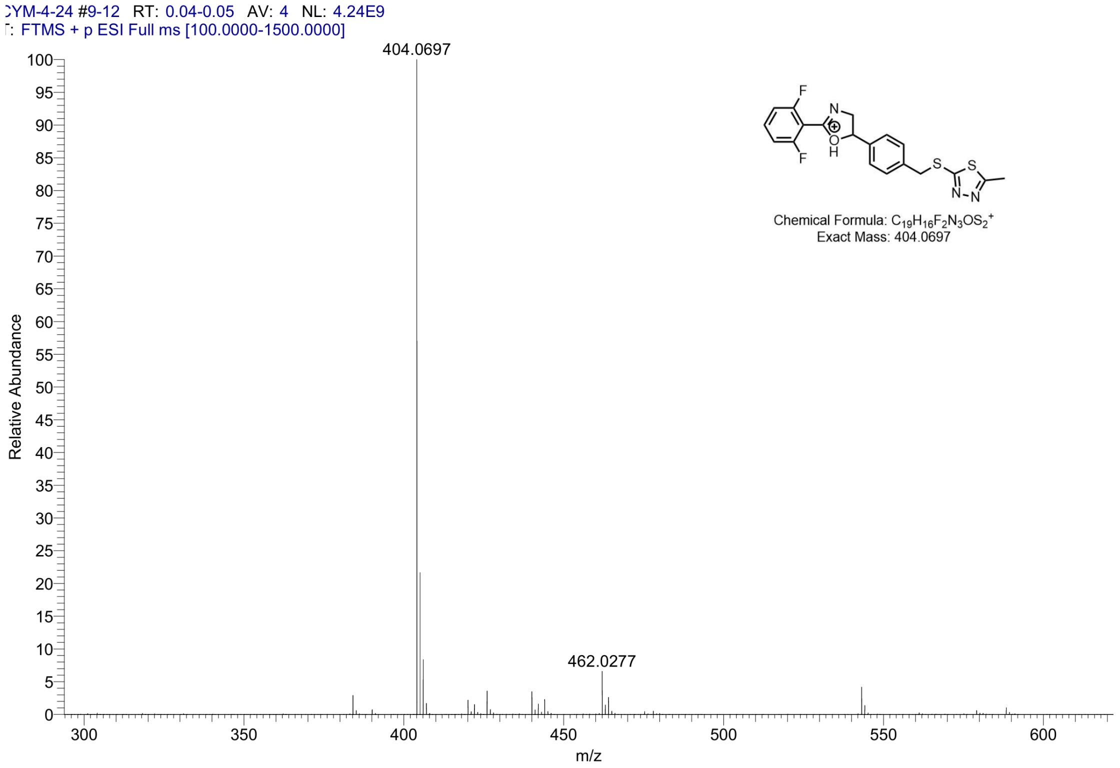

HRMS spectrum of target compound **11x**

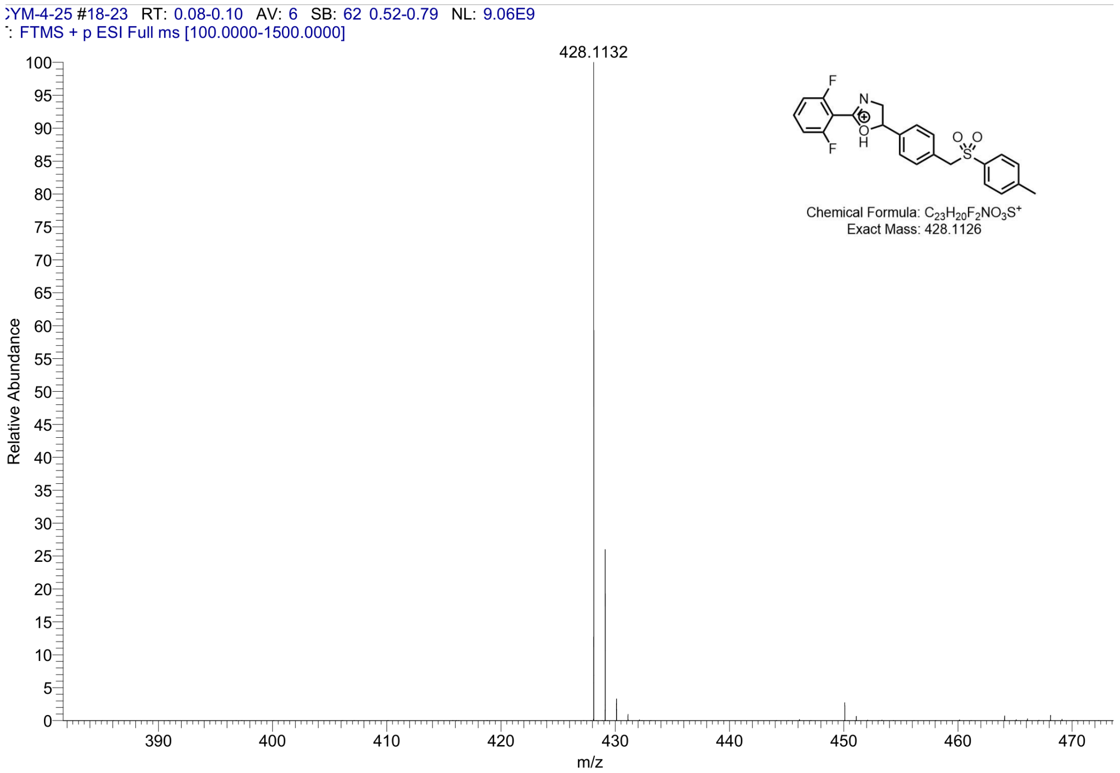

HRMS spectrum of target compound **11y**

6. Bioassay methods

Biological testing is entrusted to the Pesticide Biological Testing Center of Shenyang Sinochem Pesticide Chemical Research and Development Co., Ltd.

6.1 Test subjects

Carmine Spider Mite *(Tetranychus cinnabarinus)*

6.2 Pharmaceutical preparation

Accurately weigh the test sample, dissolve it completely with a small amount of acetone, and add 0.1% Tween-80 water solution to prepare a certain concentration of stock solution. Then, dilute it with 0.1% Tween-80 water solution according to the experimental design concentration to form a series of drug solutions.

6.3 Test methods

Refer to the Agricultural Industry Standard of the People's Republic of China - Guidelines for Laboratory Bioassay Test of Pesticides (Pesticides) Part 9: Pesticide spray Method (NY/T 1154.9-2008).

Determination of incubation inhibition rate of mite eggs: First select potted kidney bean seedlings with the same size, then select appropriate kidney bean leaves, make leaf discs with the same size, place them on the petri dish containing moisturizing filter paper, connect 7 female adult mites on each leaf disc to lay eggs, then remove the female adult mites, count the number of mite eggs with an electronic microscope, number them for standby. And then evenly spray using manual spray airbrush according to the experimental design from low dose to high dose, repeat each treatment for 3 times, and set a blank control. When the blank control mite eggs are fully hatched, investigate and count the number of incubated juvenile mites and the number of unincubated eggs on each leaf plate, and calculate the incubation inhibition rate.

Determination of acaricidal activity against mite larvae: Connect female adult mites onto newly grown intact bean seedlings with two leaves, cut the seedlings and place them in a greenhouse with light at 25 °C, and let lay eggs in the greenhouse. Then, remove the adult mites and retain the mite eggs. The mite eggs will continue to be cultured in the greenhouse for 5 days until they hatch into mite larvae, count the number of mite larval with an electronic microscope, number them for standby. Evenly spray using manual spray airbrush according to the experimental design from low dose to high dose, repeat each treatment for 3 times, and set a blank control. Continue to cultivate for 4 days, count the number of live mite larvae and the number of live mite

larvae on each leaf plate, and calculate the mortality rate.

6.4 Statistical analysis

Incubation inhibition rate or mortality was based on a percentage scale of 0−100, where 0 equals no activity and 100 equals total kill. All data were corrected with Abbott's formula. That is to say, if the percentage mortality of the control was less than 5%, the result was directly used; but if the percentage mortality was less than 20%, the result was corrected by V=((X-Y)/X)*100 (V = value of corrected mortality, X = livability of the control, Y = livability of the treat). Each treatment was carried out three times. The percentage mortality data given in Tables are means ± SDs of the three replicates.

7. References

[S1] Denmark, S. E.; Butler, C. R. Vinylation of Aryl Bromides Using an Inexpensive Vinylpolysiloxane. *Org. Lett.* **2006**, *8*, 63−66.

[S2] Song, S.; Huang, X.; Liang, Y.-F.; Tang, C.; Li, X.; Jiao, N., From simple organobromides or olefins to highly value-added bromohydrins: a versatile performance of dimethyl sulfoxide. *Green Chem.* **2015**, *17*, 2727-2731.
